# Grain zinc, iron and protein concentrations of contemporary wheat cultivars fall short of targets for human health

**DOI:** 10.1038/s43016-026-01314-3

**Published:** 2026-03-09

**Authors:** Mina Devkota, Gudeta W. Sileshi, Kalimuthu Senthilkumar, Martin R. Broadley, Dominic Mutambu, Andrew Sila, Krishna Devkota, Govinda Rizal, Job Kihara

**Affiliations:** 1 International Center for Agricultural Research in the Dry Areas (ICARDA), Rabat, Morocco; 2 Department of Plant Biology and Biodiversity Management, Addis Ababa University, Addis Ababa, Ethiopia; 3 Africa Rice Center (AfricaRice), Antananarivo, Madagascar; 4 Rothamsted Research, Harpenden, UK; 5 School of Biosciences, University of Nottingham, Loughborough, UK; 6 International Center for Tropical Agriculture (CIAT), ICIPE Duduville Complex, Nairobi, Kenya; 7 Department of Environmental Systems Science, ETH Zurich, Zurich, Switzerland

**Keywords:** Agriculture, Plant breeding

## Abstract

Grain zinc (Zn), iron (Fe) and protein concentrations have declined in wheat cultivars released since the 1960s. Here we conducted a meta-analysis of field studies to provide a global synthesis of how genetic, environmental and agronomic factors influence grain Zn, Fe and protein concentrations. The probability of achieving the Zn target (38 mg kg^−1^) was 38.9% across bread wheat and 42.7% of durum wheat grain samples, but only 28.5% of released bread wheat cultivars met this target. The probability of achieving the Zn target was 44.7% with Zn-biofortified cultivars but only 24% with non-fortified cultivars. The likelihood of achieving the Fe target (59 mg kg^−1^) was <8% across bread and durum wheat grain samples. Relative to nitrogen, phosphorus and potassium fertilizers, co-application of Zn and Fe increased grain Zn, Fe and protein concentrations by 27%, 41% and 25%, respectively. Combining agronomic and genetic biofortification is essential for improving grain nutrient concentrations and addressing micronutrient deficiencies.

## Abstract (web summary)

A synthesis of 243 field studies shows that grain zinc, iron and protein concentrations in modern wheat cultivars have declined since the 1960s, with most varieties falling short of recommended nutritional targets. While agronomic and genetic biofortification can improve these traits, the likelihood of reaching target nutrient levels remains low, highlighting the need for integrated strategies.

## Main

Hidden hunger, caused by the insufficient intake of vitamins and essential micronutrients, along with high concentrations of antinutritional factors, remains a pervasive global issue^1–3^. A recent global analysis estimates that 65% and 46% of the global population suffer from inadequate iron (Fe) and zinc (Zn) intake, respectively^2^. Epidemiological studies indicate that at least one in five individuals is at risk of Zn deficiency, partially attributed to diets with low Zn and high phytate concentrations^3,4^. Populations that rely heavily on cereals (wheat, rice and maize) as dietary staples are particularly impacted by Zn and Fe deficiencies^5,6^.

Over the past few decades, substantial investments have been directed towards the development of high-yielding cultivars with traits such as disease resistance, drought tolerance and enhanced grain quality^7^. Although these efforts have boosted wheat productivity, they may have concurrently led to a decline in nutritional quality^8–12^. As a key global staple, wheat plays a critical role in food security^13,14^, being cultivated in over 120 countries^13^ covering 218 Mha of land globally^15^. Given its global prominence, nutritionally enhanced wheat can deliver higher levels of micronutrients to populations that depend on wheat as a staple food^16^.

Cultivated wheat represents a diverse group of species, including bread (*Triticum aestivum* subsp. *aestivum*), durum (*Triticum turgidum* subsp. *durum*), dicoccum (*Triticum dicoccum*), einkorn (*Triticum monococcum*), emmer (*T. turgidum* subsp. *dicoccum*) and spelt (*T. aestivum* subsp. *spelta*). Spelt wheat, a cross between emmer wheat and goatgrass (*Aegilops tauschii*)^17^, was one of the earliest cultivated crops and served as the primary bread cereal in Europe until the beginning of the twentieth century^18,19^. Over time, spelt wheat gradually lost its importance, leading to a decline in its cultivated area^19^. Today, smaller quantities of spelt, emmer and einkorn are cultivated in parts of the European Union, the Balkans and the Indian subcontinent^17,19^. Bread wheat now accounts for approximately 95% of global wheat production, while durum wheat constitutes approximately 5% of the total output^20^. Wheat provides approximately 35–40% of the global population’s caloric intake; however, it is inherently deficient in key micronutrients such as Zn and Fe^1,11,12^. Wheat production on nutrient-deficient soils further decreases micronutrient concentrations in grains^11^. Excessive application of phosphorus (P) fertilizers in Zn-deficient soils exacerbates the problem by elevating grain phytate concentrations^21^. Phytate, an antinutritional factor, binds to Zn and Fe, thereby reducing their bioavailability in the human diet^21^. Addressing the dual challenge of improving both grain yield and nutritional quality is thus a critical global priority. Current breeding programmes have focused on incorporating genes that enhance Zn levels from sources along with other high-Zn and Fe donors into high-yielding cultivars^22^. Despite these efforts, it remains unclear to what extent these strategies have achieved the breeding targets set by HarvestPlus (38 mg kg^−1^ for Zn and 59 mg kg^−1^ for Fe), which is considered sufficient to meet 30% of human nutritional requirements^23^. Furthermore, there is a lack of comprehensive analysis on how these agronomic and genetic interventions have impacted concentrations of Zn, Fe and protein in wheat grains over time. Here we consolidate evidence on genetic and agronomic biofortification of wheat for Zn, Fe and protein to inform policies and guide research investment. The objectives are to assess global trends in grain Zn and Fe concentrations and yield; evaluate variation in Zn, Fe, protein and phytate across genotypes, management practices, soil types and climates; and examine their relationships with grain yield.

## Results

### Distributions of grain Zn, Fe, protein and phytate concentrations

**Fig. 1:**
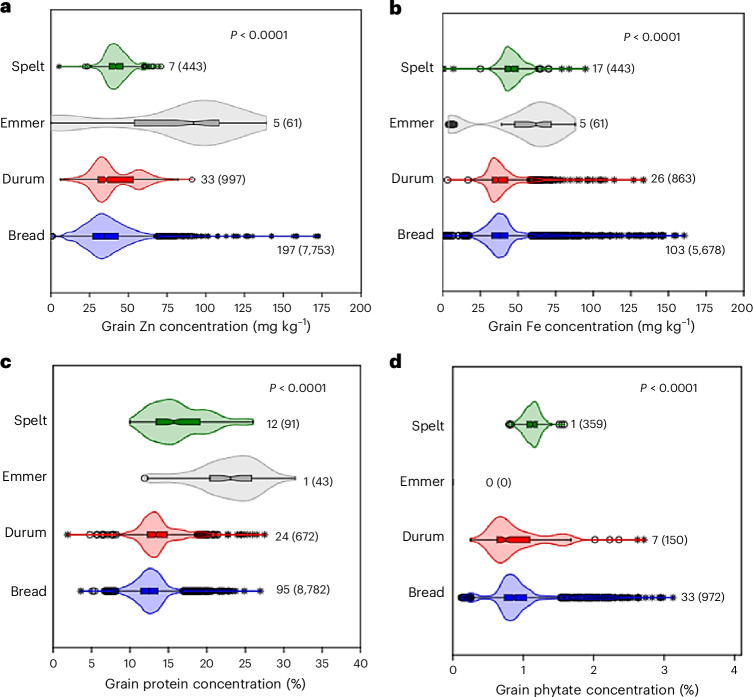
Variations in grain Zn, Fe, protein and phytate concentrations among different species of cultivated wheat on a dry weight basis. **a**–**d**, Grain Zn (**a**), Fe (**b**), protein (**c**) and phytate (**d**) concentrations. The values beside each violin represent the number of studies and the total number of observations (in parentheses) available. The *P* values correspond to the Kruskal–Wallis test assessing equality of medians, with *P* < 0.05 indicating a significant difference between sample medians. The whiskers represent the most extreme points (minima and maxima). The circles represent possible outliers, while the stars represent probable outliers. The notches in the box plots represent an approximate 95% CI of the median. Source data

**Fig. 2:**
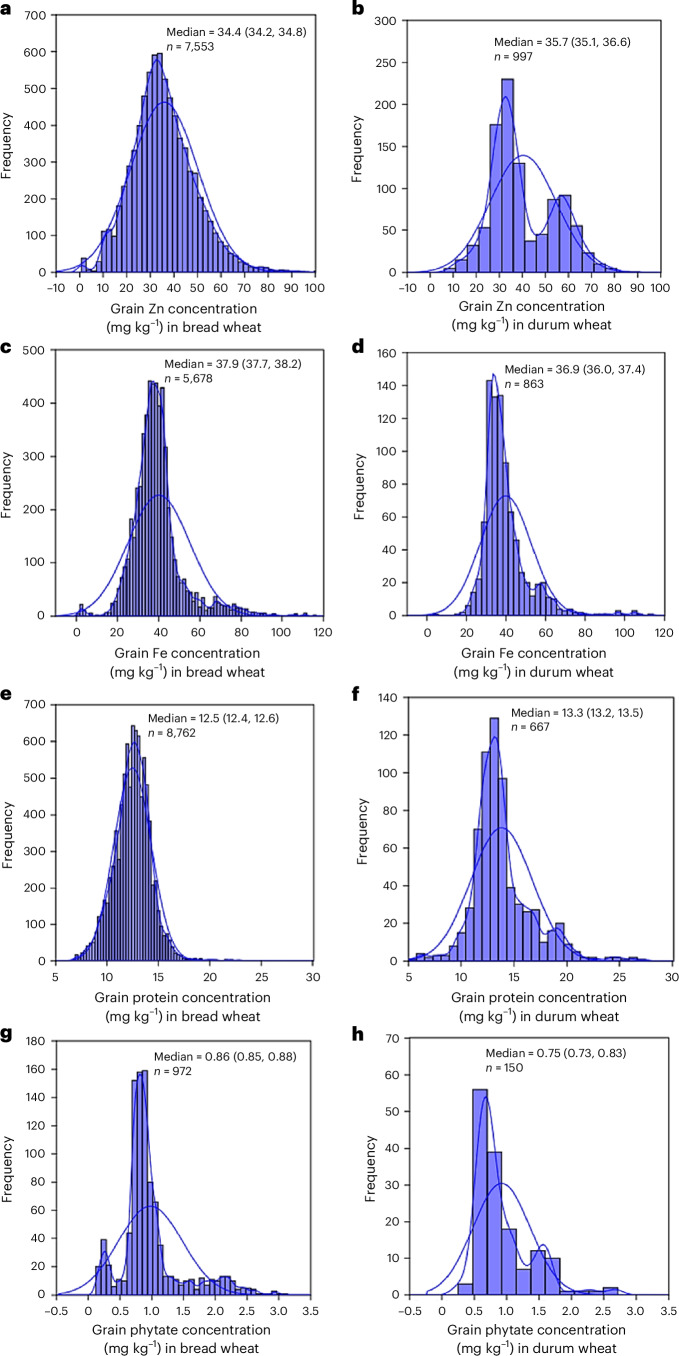
Empirical distributions of grain Zn, Fe, protein and phytate concentrations on a dry weight basis in bread and durum wheat. **a**–**h**, Grain Zn concentration in bread (**a**) and durum (**b**) wheat, grain Fe concentration in bread (**c**) and durum (**d**) wheat, protein concentration in bread (**e**) and durum (**f**) wheat, and phytate concentration in bread (**g**) and durum (**h**) wheat. The jagged and smooth lines represent the kernel density and normal probability distributions, respectively, of each variable. Values in parentheses following the median values represent the 95% CI of medians. The *n* represents the total number of observations. Source data

The empirical distributions of grain Zn, Fe, protein and phytate concentrations were derived from data collected in 243 field studies conducted across 41 countries (Supplementary Figs. 1 and 2 and Supplementary Table 1). For bread wheat, the data included concentrations of Zn, Fe, protein and phytate, consisting of (1) wheat landraces, (2) released varieties, (3) non-biofortified cultivars and (4) Zn-biofortified cultivars as separate categories. All reported cultivars of durum, emmer and spelt wheat are non-Zn-biofortified. A large variability was found among countries in the total number of observations available for analysis (Supplementary Fig. 3). The median grain Zn, Fe, protein and phytate concentrations in bread and durum wheat were significantly lower than those observed in spelt and emmer wheat (Fig. 1 and Supplementary Table 2). The median grain Zn concentration in bread wheat (34.4 mg kg^−1^) was lower than that in durum wheat (35.7 mg kg^−1^) (Fig. 2 and Supplementary Table 2). The median grain Fe concentration in bread wheat (37.9 mg kg^−1^) was higher than that in durum wheat (36.9 mg kg^−1^) (Fig. 2 and Supplementary Table 2). Emmer wheat had significantly higher concentrations of grain Zn, Fe and protein compared with bread and durum wheat (Fig. 1).

The probability of achieving the Zn concentration target of 38 mg kg^−1^ was 38.9% across bread wheat and 42.7% of durum wheat grain samples, but significantly higher in emmer wheat (80.3%) and spelt (72.0%) grain samples (Supplementary Table 2). The probability of achieving the target Fe concentration of 59 mg kg^−1^ was only 7.7% in bread wheat, 6.6% in durum wheat and 4.7% in emmer wheat grain samples. By contrast, the corresponding probability of exceeding the Fe target in spelt wheat grain samples was 59% (Supplementary Table 2a). When comparing treatments with and without Zn application, the probability of exceeding the Zn target was 56.4% in bread wheat where Zn was applied, but only 33.5% in the absence of Zn application (Supplementary Table 2b). Grain Zn concentrations exceeded the target of 38 mg kg^−1^ in 82% of the landraces in the absence of Zn fertilization, but this target was met in 100% of the landraces where Zn fertilizers were applied (Supplementary Table 2c). For released bread wheat varieties, grain Zn concentrations exceed 38 mg kg^−1^ in only 28.5% of cases without Zn fertilization but in 66.4% with Zn fertilization (Supplementary Table 2c). However, the probability of grain Fe concentrations surpassing 59 mg kg^−1^ remained low either with Zn fertilization (7.7%) or without Zn application (7.1%) (Supplementary Table 2).

The median grain protein concentration in bread wheat (12.5%) was significantly lower than that in durum wheat (13.3%), spelt (15.7%) and emmer (23.1%). The probability of exceeding the grain protein concentration of 12% was 63.8% in bread wheat but substantially higher (94–98%) in emmer and spelt wheat (Supplementary Table 2). However, the median phytate concentration in bread wheat (8.5 mg g^−1^) was significantly higher than that in durum wheat (7.1 mg g^−1^), while no estimates were available for emmer wheat (Fig. 1d).

### Genotype-by-environment interactions and heritability of traits

In almost all studies, environment and the genotype-by-environment (G × E) interaction accounted for a larger percentage of the variance in grain yield and grain Zn, Fe and protein concentrations than the genotype alone (Supplementary Table 3). The G × E interaction was statistically significant in most studies. The broad-sense heritability of grain Zn and Fe concentrations varied between 0.15 and 0.99 (Supplementary Table 4). By contrast, broad-sense heritability was generally high (0.56–0.91) for grain protein concentrations (Supplementary Table 4). None of the studies have reported broad-sense heritability or G × E interactions for grain phytate concentrations.

### Associations between grain yield and Zn, Fe, protein and phytate concentrations

Significant negative correlations were found between grain yield and grain Zn concentrations in 31.6% and 28.8% of the studies evaluating wheat germplasm and fertilization effects, respectively. However, significant negative correlations were found between grain yield and grain protein concentrations in 61.5% of the studies on germplasm and 25.8% of the fertilization trials. Grain Zn concentrations were significantly and positively correlated with grain Fe concentrations across all three wheat species (Supplementary Table 5). In bread wheat, grain Zn and Fe concentrations were significantly positively correlated in 73.3% and 50% of the studies on germplasm and fertilization, respectively. Significantly negative correlations between grain Zn and Fe concentrations were rare. Regarding phytate, the overall correlations with grain yield were positive, while the correlations between grain phytate, Zn and Fe concentrations were negative in both bread and durum wheat (Supplementary Table 5). In bread wheat, grain Zn and phytate concentrations were negatively correlated in 17% and 25% of the studies that evaluated germplasm and fertilization, respectively. In four out of the five studies that reported grain phytate and Fe concentrations, the correlations were non-significant between the two variables (Supplementary Table 5).

### Trends in grain yield and Zn, Fe and protein concentrations with release dates of varieties

**Fig. 3:**
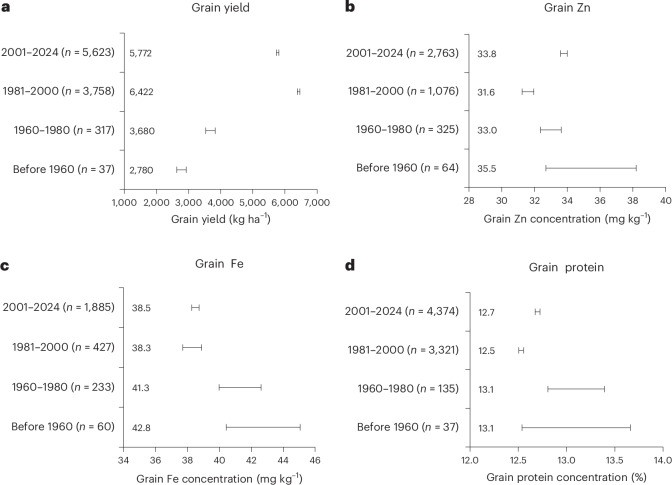
Trends in grain yield and Zn, Fe and protein concentrations on a dry weight basis in bread wheat cultivars over the years. **a**–**d**, Grain yield (**a**) and grain Zn (**b**), Fe (**c**) and protein (**d**) concentrations. Horizontal bars represent the 95% CIs of medians generated using a BCa bootstrapping method with 9,999 replicates. The *n* and values after the years represent the total number of observations and median values, respectively. Source data

Grain yields have significantly increased in bread wheat varieties released between 1960 and 2024 (Fig. 3a). However, grain Zn, Fe and protein concentrations show a significant downward trend in varieties released over the same period (Fig. 3b–d). A steeper decline was observed in grain Fe concentrations than in Zn concentrations.

### Effect of genetic biofortification

Analysis of studies comparing genetically biofortified cultivars with non-biofortified varieties focused only on Zn-biofortified wheat because the necessary data were not available for Fe-biofortified cultivars. Zn-biofortified cultivars achieved a modest increase in grain yield (~7%) compared with non-biofortified cultivars (Table 1). Zn biofortification also resulted in 18.5%, 20% and 19.7% higher grain Zn, Fe and protein concentrations, respectively, relative to non-biofortified cultivars. Moreover, the probability of grain Zn concentrations exceeding the target of 38 mg kg^−1^ was substantially higher in Zn-biofortified cultivars (44.7%) than in non-biofortified cultivars (24.0%) under identical agronomic management. The probability of exceeding the grain Fe concentration target of 59 mg kg^−1^ was only 2% in Zn-biofortified bread wheat cultivars (Table 1). The response ratios (RRs) of grain yield and Zn, Fe, protein and phytate concentrations also did not show significant differences between Zn-biofortified and non-biofortified bread wheat cultivars (Table 1).

**Table 1:** Comparison of non-biofortified and Zn-biofortified bread wheat cultivars

Variable	Status (studies, *n*)^a^	Median (95% CI)	*ϕ* > target	RR (95% CI)
Grain yield (kg ha^−1^)	Non-biofortified (14, 438)	3,130 (3,020, 3,210)	NA	1.06 (1.05, 1.12)
	Zn-biofortified (15, 279)	3,352 (3,321, 3,500)	NA	1.06 (0.99, 1.17)
Zn (mg kg^−1^)	Non-biofortified (20, 463)	29.8 (28.7, 31.0)	24.0	1.64 (1.47, 1.76)
	Zn-biofortified (22, 389)	35.3 (34.5, 38.1)	44.7	1.30 (1.15, 1.68)
Fe (mg kg^−1^)	Non-biofortified (15, 292)	34.0 (32.9, 35.0)	1.4	1.07 (0.99, 1.23)
	Zn-biofortified (17, 357)	40.8 (39.9, 41.7)	2.0	1.05 (1.04, 1.29)
Protein (%)	Non-biofortified (6, 79)	11.7 (10.6, 12.2)	NA	1.04 (1.03, 1.14)
	Zn-biofortified (8, 73)	14.0 (13.7, 15.7)	NA	1.03 (0.96, 1.09)
Phytate (%)	Non-biofortified (5, 142)	0.76 (0.74, 0.78)	NA	0.99 (0.98, 1.01)
	Zn-biofortified (5, 20)	0.82 (0.74, 0.91)	NA	1.01 (1.00, 1.13)

There are no released varieties of Fe-biofortified wheat. Dry-weight grain Zn and Fe concentrations are shown. Data are presented as mean values and 95% CIs. Values in parentheses are 95% CIs of medians. Non-biofortified and genetically biofortified cultivars are deemed significantly different if their 95% CIs do not overlap.

NA, not applicable.

^a^Studies and *n* represent the total number of studies and number of observations, respectively.

### Effect of agronomic variables

The agronomic variables analysed in this study included nitrogen (N), phosphorus (P) and potassium (K) fertilizer application rates, the methods and rates of Zn and Fe application, and irrigation practices in bread wheat. Therefore, the subsequent discussion primarily focuses on the response of bread wheat to N, P, K, Zn and Fe fertilizers and irrigation. Meta-analysis could not be performed on other agronomic variables such as organic inputs and tillage practices because most studies were conducted under conventional tillage without organic fertilizers. This, along with the normal quantile–quantile plots, indicates the presence of some publication bias (Supplementary Fig. 5).

#### Response to N, P, K, Zn and Fe fertilizers

In both bread and durum wheat, treatments without Zn had significantly lower response ratios for grain yield and Zn, Fe and protein concentrations than treatments that included Zn (Supplementary Fig. 6). The other treatments without Zn (‘others’) did not enhance grain yield or Zn, Fe and protein concentrations by more than 10% over the NPK control (Supplementary Table 7). These results indicate that nitrogen, phosphorus and potassium (NPK) fertilizers alone, or in combination with other treatments excluding Zn, do not lead to substantial improvements in grain Zn, Fe or protein concentrations. The response ratios of grain yield and Zn, Fe and protein concentrations in bread wheat were positively correlated with application rates of N and P (Supplementary Table 8). Locally estimated scatter plot smoothing (LOESS) regression also revealed an increasing trend in grain yield in response to N fertilizer rates (Supplementary Fig. 7a). Grain Zn, Fe and protein concentrations also increased with N application rates up to 100 kg ha^−1^, after which no further increase was observed (Supplementary Fig. 7b–d). The LOESS regression of grain yield and nutrient concentrations against P fertilizer rates revealed a contrasting trend (Supplementary Fig. 8a–e). Increasing P application tended to reduce grain Zn concentrations when P rates exceed 45 kg ha^−1^, relative to NPK fertilizers (Supplementary Fig. 8b), but a sharp increase in grain phytate concentrations results when P rates exceeded 90 kg ha^−1^ (Supplementary Fig. 8e).

Treatments involving Zn with or without Fe (hereafter Zn ± Fe) fertilizers significantly increased grain yield, as well as grain Zn, Fe and protein concentrations in bread wheat, relative to the NPK control (Supplementary Table 7). This increase was consistent across spring, winter and facultative bread wheat cultivars (Supplementary Fig. 6). The largest increase in grain Zn concentrations was achieved with a combination of soil and foliar Zn application (65%), followed by foliar Zn alone (54%) relative to NPK fertilizers (Supplementary Table 7). The largest increase in grain yield (26%) was achieved with soil application of Zn and Fe together (Supplementary Table 7). Soil application of Zn and Fe fertilizers also significantly improved grain Zn by 32%, while foliar application of only Zn resulted in a 54% increase in grain Zn concentrations (Supplementary Table 7). Soil application of Zn combined with Fe was the most effective approach for simultaneously increasing grain Zn, Fe and protein concentrations in bread wheat (Supplementary Table 7). Grain phytate concentrations were significantly reduced with both soil and foliar Zn applications relative to NPK fertilizers (Supplementary Table 7).

#### Irrigation

Zn (±Fe) fertilization significantly increased grain yield and Zn, Fe and protein concentrations relative to NPK fertilizers under both irrigated and rain-fed conditions (Supplementary Table 9). However, greater increases in grain Zn and protein concentrations were achieved under irrigated conditions than under rain-fed conditions with Zn fertilization. With all treatments, greater grain protein concentrations were achieved under irrigated conditions. With Fe fertilization, greater increases in grain Zn concentrations were achieved in rain-fed conditions (Supplementary Table 9). Greater increases in grain Fe concentrations were also achieved with Zn + Fe fertilization under rain-fed conditions.

### Effects of soil variables

#### Soil texture, parent material and soil type

**Fig. 4:**
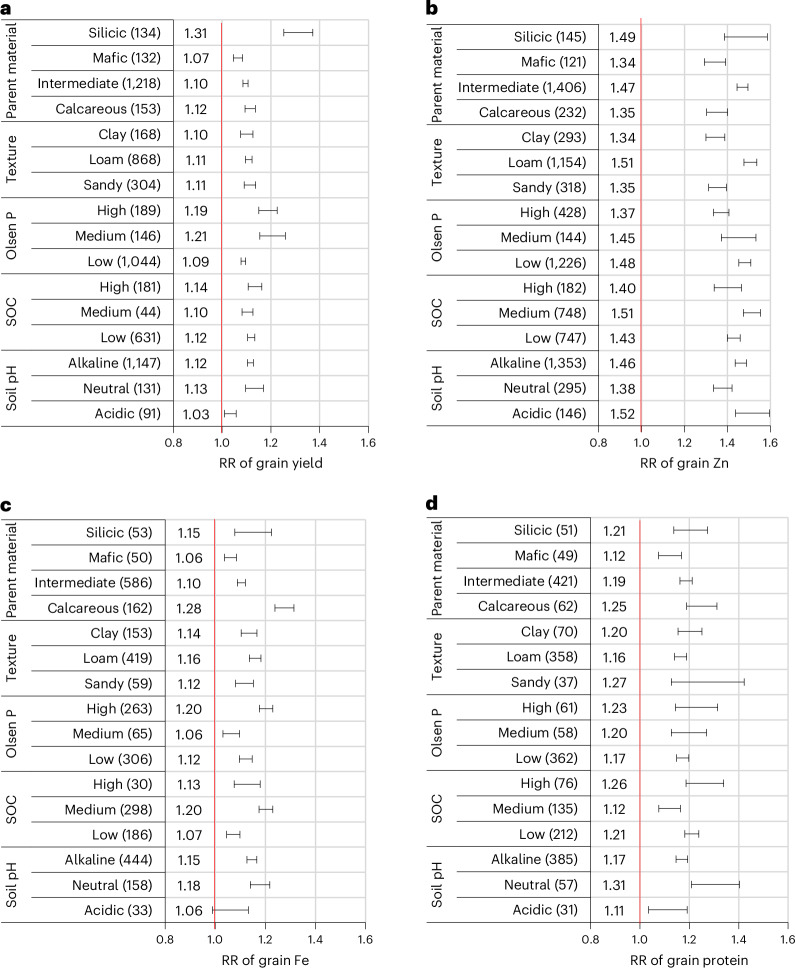
Variations in response ratios of grain yield and grain Zn, Fe and protein concentrations of bread wheat with soil variables in treatments involving application of Zn and Fe. **a**–**d**, RR of grain yield (**a**) and grain Zn (**b**), Fe (**c**) and protein (**d**) concentrations. The horizontal bars represent the 95% CIs. When the CIs encompass RR = 1, the Zn (±) treatment is not significantly different from the NPK control. Values in parentheses after the soil type represent the total sample sizes, while those on the left side of the horizontal bars represent the marginal means. Source data

Compared with NPK fertilizer, the application of Zn (±Fe) fertilizers significantly increased grain yields and Zn, Fe and protein concentrations across all parent materials, soil texture classes (Fig. 4) and soil types (Supplementary Fig. 10). The greatest increases in grain yield (31%) and Zn concentration (49%) were observed on soils derived from silicic parent material, while higher grain Fe (28%) and protein (25%) concentrations were achieved on calcareous soils (Fig. 4c,d).

Although Zn (±Fe) fertilization significantly increased grain yield and grain Zn, Fe and protein concentrations across most soil types, the magnitude of increases over NPK fertilizers varied with soil type (Supplementary Fig. 10). Relative to NPK fertilizers, Zn (±Fe) fertilization resulted in the highest grain yield increases (30–33%) on Leptosols, Regosols and Solonetz soils. Zn (±Fe) fertilization also led to an increase in grain Zn concentrations on Solonchaks, which are saline soils typically found in arid and semi-arid regions. The largest increase in grain Fe concentration (52%) was observed on Solonetz, which is characterized by high levels of exchangeable sodium and magnesium ions. The sample sizes were too small on certain soil types, so the results should be interpreted with caution.

#### Soil pH

Regardless of soil pH levels, Zn (±Fe) fertilization significantly increased grain yield and grain Zn, Fe and protein concentrations relative to NPK fertilizers (Fig. 4). However, greater increases in grain yield and protein concentrations were observed on neutral soils than acidic or alkaline soils (Fig. 4). The highest increase in grain Fe concentrations was achieved on alkaline soils. By contrast, significantly higher increases in grain Zn concentrations were recorded on acidic soils than on neutral or alkaline soils.

#### Soil organic carbon concentrations

Regardless of soil organic carbon (SOC) levels, Zn (±Fe) fertilization significantly increased grain yield and grain Zn, Fe and protein concentrations relative to NPK fertilizers (Fig. 4). However, the greatest increases in grain Fe concentrations were observed on soils with SOC > 1% (Fig. 4c).

#### Soil total N, available P (Olsen), K, Zn and Fe concentrations

The response ratio of grain yield was positively correlated with total N and Olsen P in the soil (Supplementary Table 8). Similarly, the response ratio of grain Zn concentrations was positively correlated with soil total N, available K and Zn (Supplementary Table 8). Grain Zn concentration was positively correlated with soil Zn concentration (Supplementary Table 5).

### Effects of climate variables

Relative to NPK fertilizers, Zn ± Fe fertilizers combined with the different inputs consistently increased grain yield and Zn concentrations across various climate zones (Supplementary Fig. 12a,b) and all continents (Supplementary Fig. 11a,b). The largest increase in grain Zn concentrations (66%) was observed in boreal zones (Supplementary Fig. 12b), while the greatest increase in grain Fe concentrations was observed in subtropical climates (Supplementary Fig. 12c). The results provide strong evidence that Zn ± Fe fertilization can enhance grain yield and grain Zn, Fe and protein concentrations regardless of the climate zone. Owing to limited data for wheat, we could not perform a meta-analysis of the effects of climate change, such as elevated CO_2_ and temperature, on grain yields and grain Zn, Fe and protein concentrations.

### Relative importance of variables

We performed random forest (RF) models combining genotypic, environmental and management variables for which data were available to identify the most influential variables. The variables included in the RF models explained 75%, 72%, 93% and 54% of the variation in grain yield and grain Zn, Fe and protein concentrations, respectively (Supplementary Fig. 13). Because of sparse data, the RF model did not provide the variable importance plot for phytate concentration. We were also unable to produce the partial dependency plots of the various factors owing to sparsity of the data.

**Fig. 5:**
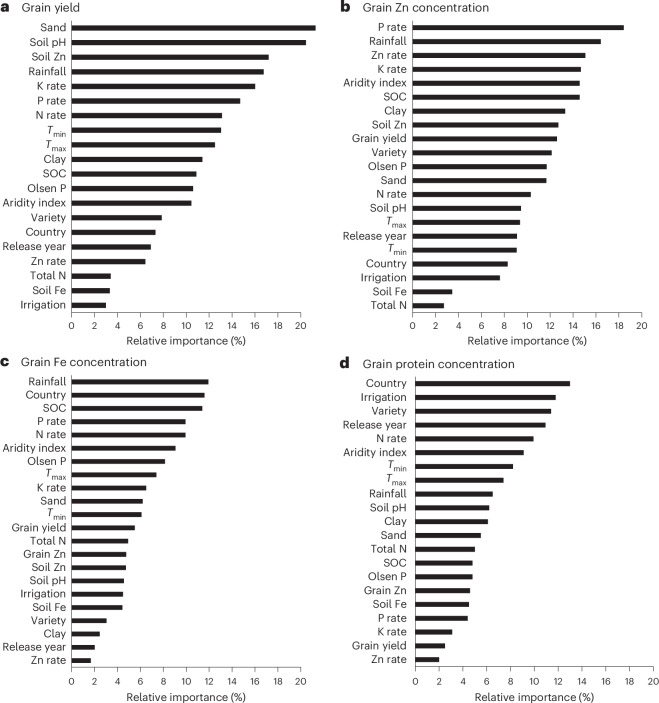
Relative importance of different predictors used in RF analysis for variation in grain yield and grain Zn and Fe concentrations. **a**–**d**, Grain yield (**a**) and grain Zn (**b**), Fe (**c**) and protein (**d**) concentrations. Rainfall, in-season rainfall; *T*_max_, maximum temperature in the growing season; *T*_min_, minimum temperature in the growing season; Release year, variety release year; Total N, soil total nitrogen; Soil Zn, soil-available Zn. Source data

The relative importance of variables influencing grain yield was different from those influencing grain Zn, Fe and protein concentrations (Fig. 5). For grain yield, sand content, soil pH, soil-available Zn, in-season rainfall and the K application rate were the top five influential variables (Fig. 5a). For grain Zn concentrations, P application rates, in-season rainfall, Zn and K application rates, and aridity index were the top five influential variables (Fig. 5b). In-season rainfall, country of study, SOC and P and N application rates were the five most influential variables for grain Fe concentrations (Fig. 5c), while the country of study, irrigation, variety, age of variety and N rate were the five most influential variables for grain protein concentration (Fig. 5d).

## Discussion

This analysis has provided insights into the variation in grain yield and grain Zn, Fe, protein and phytate concentrations in wheat species and modern cultivars. The findings indicate that grain Zn, Fe and protein concentrations of modern wheat cultivars fall short of their targets for human health. Although genetic and agronomic biofortification may greatly aid in enhancing progress towards the target Zn levels, they seem to have limited impact on reaching the Fe target concentration. However, the grain Zn and Fe concentrations in bread wheat are significantly higher than the baseline values proposed by HarvestPlus^23^ (25 mg kg^−1^ and 30 mg kg^−1^) and the standard reference values in the United State Department of Agriculture-Agricultural Research Service (USDA-ARS) database (26 mg kg^−1^ and 36 mg kg^−1^). HarvestPlus derived its global baseline for wheat based on data available from the Consultative Group on International Agricultural Research (CGIAR) between 2003 and 2008. Given the evidence provided by the present analysis, we propose revision of the baseline Zn and Fe values upwards to 34 mg kg^−1^ for Zn and 37 mg kg^−1^ for Fe for bread and durum wheat. The protein concentration is also lower than the desirable values for bread making. For optimal bread making, a whole-grain protein concentration exceeding 12% is required because 0.9–1.9% of wheat protein is typically lost during milling at an ~70% flour extraction rate^24^.

The preponderance of significant G × E interactions suggests a strong influence of the environment on the expression of grain Zn and Fe concentrations. This may pose challenges for direct selection of genotypes with high Zn and Fe concentrations. This also probably explains the slow progress made so far in breeding Fe-biofortified bread wheat cultivars. While phenotypic variation is largely attributable to genetic differences, heritability can vary from low to high depending on the trait and study. The low broad-sense heritability of grain Zn and Fe concentrations in some studies indicates higher environmental influences. However, heritability appears to be high enough in most cases to support genetic biofortification through conventional breeding techniques.

The analysis indicates that the magnitude and direction of the correlations between grain yield and Zn, Fe, protein and phytate concentrations can vary with the type of study, for example, between germplasm evaluation (breeding) and fertilization (agronomic) trials. In the past, some studies had reported non-significant correlations between grain yield and Zn and Fe concentrations^25^, while others have reported significant negative associations, commonly referred to as the ‘dilution’ effect^10,26,27^. The positive associations between grain yield and Zn concentrations in this analysis and the evidence from larger surveys^25^ suggest that it is possible to increase grain yield without significantly reducing grain Zn concentrations in bread wheat. The predominance of positive correlations between grain Zn and Fe concentrations is consistent with earlier studies in China^27^, Nepal^28^ and Mexico^29^. According to Liu et al.^27^, for each 1 mg kg^−1^ increase in grain Zn concentration, the Fe concentration increased by 0.3–0.6 mg kg^−1^ in bread wheat.

Grain protein concentration was negatively correlated with grain yield in bread wheat, a result that aligns with previous reports^11,30^. Significant positive correlations were observed between grain Zn, Fe and protein concentrations in bread wheat, consistent with earlier studies^10,31,32^. This may be attributed to the co-localization of Zn and Fe with protein in wheat seeds, and multiple lines of evidence suggest that protein serves as a sink for both Zn and Fe^33^. Taken together, these findings suggest that simultaneous improvement of grain Zn, Fe and protein concentrations is possible through both breeding and agronomic techniques. However, traditional breeding techniques may be inadequate to achieve the target Zn and Fe concentrations. Therefore, we recommend investments in modern breeding techniques, such as mutation breeding, transgenics, gene editing and gene overexpression to increase grain Zn and Fe in bread wheat; this is particularly important for Fe, but also relevant for Zn.

While grain yield increased over the past 60 years, grain Zn, Fe and protein concentrations have declined in the same period. These global trends are consistent with findings from country-specific studies^8,9,11,28,32,34^. For instance, Fan et al.^9^ observed evidence of declining mineral density in wheat grain over time in the UK. In India, Debnath et al.^8^ reported an ~30% reduction in Zn concentration and an ~19% reduction in Fe concentration in high-yielding wheat cultivars released between 1960 and 2010. In China’s wheat-growing regions, Liu et al.^28^ reported a 1.3–2.1 mg kg^−1^ decrease in grain Fe and a 0.9–1.3 mg kg^−1^ decrease in Zn concentration per 1,000 kg ha^−1^ increase in yield. In the Great Plains of the USA, grain Zn and Fe concentrations decreased by ~0.8 mg kg^−1^ and 2.3 mg kg^−1^, respectively, for every 1,000 kg ha^−1^ yield increase^11^. Since the introduction of dwarf wheat cultivars in the 1960s, the concentrations of micronutrients, including Zn and Fe, have declined steadily^8–12^. This trend has been observed across various regions of the world^9,12,32,33^. It is believed that improvements aimed at higher yields and stress resistance have unintentionally reduced nutritional quality traits in modern bread wheat cultivars^34^. Although some progress has been made in increasing grain Zn concentration in more recent bread wheat cultivars^25,35^, the findings from this analysis indicate that improvements have been slow.

The analysis of studies comparing genetically biofortified cultivars with non-biofortified varieties suggests that the target Zn concentrations can be achieved through the complementary use of genetic and agronomic interventions. These findings also highlight the need for Zn biofortification to augment grain Fe and protein concentrations without a yield penalty. Zn-biofortified bread wheat cultivars were equally responsive to Zn fertilization as non-biofortified cultivars. The results of this analysis also provide evidence that the nutritional traits of wheat can be simultaneously improved through the complementary use of agronomic and genetic biofortification. Among the agronomic options, soil application of Zn + Fe has emerged as the most effective strategy for improving grain Zn, Fe and protein concentrations, particularly because it is more practical and feasible than foliar applications for broad-acre crops. In regions with limited access to fertilizers, genetic biofortification may offer a sustainable and long-term solution.

The trends found in this analysis suggest that grain Zn, Fe and protein concentrations can be concurrently increased with N application rates up to 100 kg ha^−1^. These trends are consistent with findings from long-term studies, which suggest synergistic increases in grain Zn and Fe concentrations with N fertilization up to a certain threshold^11,35,36^. According to Chen et al.^35^, an N application rate of 155 kg ha^−1^, which is considered optimal for grain yield, also resulted in higher grain Zn concentrations. However, increasing the N application rate from 130 kg ha^−1^ to 300 kg ha^−1^ did not yield further improvements in grain Zn and Fe concentrations^35,36^. These findings suggest that applying N fertilizers with the goal of enhancing grain yield can also lead to increases in grain protein, Zn and Fe concentrations in wheat.

Unlike N fertilizer, high P application rates appear to reduce grain Zn concentrations while increasing phytate concentrations. These results are consistent with findings from country-level studies^35,37,38^. For instance, Zhang et al.^38^ reported decreases in grain Zn and protein concentrations, and increases in phytic acid concentrations, with P application rates exceeding 50 kg ha^−1^. Similarly, in a survey of 304 fields across China’s Loess Plateau, Li et al.^27^ found that the target grain Zn concentration in wheat could not be achieved when P fertilizer rates exceeded 111 kg P ha⁻^1^. The decrease in grain Zn concentration due to elevated Olsen P has also been observed in bread wheat following continuous P application on calcareous soils over four cropping seasons^35^. The P-induced Zn deficiency is attributed to several mechanisms^39^. In regions where high rates of P fertilizers are commonly used or where soils are naturally rich in available P, current P fertilizer recommendations should be re-evaluated.

The increases in grain yield and grain Zn, Fe and protein concentrations in bread wheat achieved with Zn fertilization are in agreement with a recent meta-analysis^40^. These results, along with the positive correlation between grain Zn and Fe concentrations (Supplementary Table 5), suggest that Zn fertilization leads to Zn- and Fe-dense grains. However, increasing Zn rates beyond 40 kg ha^−1^ tended to depress grain yields relative to NPK fertilizers, consistent with country-level reports^35^. For instance, in a study involving 320 paired-plot field experiments across China^35^, an average increase of 10.5 mg kg^−1^ in grain Zn concentration was recorded with foliar Zn application in bread wheat. Foliar Zn application tends to be more effective than soil application, particularly under moisture-limited conditions, as Zn transport to plant roots occurs primarily via diffusion in water^41^. Significant advances have been made in formulating Zn fertilizers for foliar application^42^, offering promising opportunities for agronomic biofortification of wheat with Zn. However, soil application of Zn combined with Fe appears to be the most effective approach for simultaneously increasing grain Zn, Fe and protein concentrations in bread wheat. This suggests that Zn fertilization achieves greater increases in Zn concentrations under rain-fed conditions than irrigated rice. This is consistent with the notion that plants become more sensitive to Zn deficiency under rain-fed conditions^43^.

The greatest increases in grain Fe and protein concentration were achieved with Zn ± Fe fertilization on calcareous soils. The high bicarbonate concentrations typically present in calcareous soils often induce Fe deficiency^44^. Under such conditions, Zn ± Fe fertilization can enhance the availability of these micronutrients.

The analysis further revealed positive correlations between improvements in grain Zn concentrations and soil total N, available K and Zn concentrations. These findings are in agreement with earlier studies on the Loess Plateau of China, where a positive correlation was found between grain Zn concentrations and mineral N across 120 fields^25^. However, the correlation between the response ratio of grain Zn concentrations and available P was not statistically significant, despite previous studies^25^ reporting a strong negative correlation between grain Zn and available P. This was linked to reduced Zn availability in the rhizosphere, lower Zn uptake, decreased mycorrhizal colonization, reduced root-to-shoot Zn transport and yield-induced dilution effects^37,39^. Given the synergistic relationship between soil N and Zn, and the antagonistic effect of P on Zn^45^, optimizing N and P application rates to simultaneously achieve high grain yields and enhanced grain Zn bioavailability remains challenging under field conditions^25^. The positive correlation between grain Zn concentration and soil Zn concentration observed in this analysis is consistent with observations across the 120 sites in the Loess Plateau of China^25^. When wheat is grown on Zn-deficient soils, grain Zn levels may fall below the thresholds required to meet human nutritional needs^1^. Together, these findings highlight the need for appropriately targeted Zn and Fe fertilization based on soil conditions.

The gains in nutrient and protein concentrations that can be achieved by agronomic or genetic fortification may be offset by the anticipated climate-change-related problems such as elevated atmospheric carbon dioxide (CO_2_)^4,46–48^. Owing to the limited data for wheat, we could not perform a meta-analyse of the effects of elevated CO_2_ and temperature on grain yields and grain Zn, Fe and protein concentrations. Instead, we reviewed existing meta-analyses^46,49–51^ and primary studies on wheat grain yield and grain nutrient concentrations^47,52^ to provide a brief synthesis of the existing evidence on climate change effects. According to one meta-analysis^49^, elevated CO_2_ (450–800 ppm) increased grain yield by 24%, which was consistent with another meta-analysis^50^ showing 30% increase in the grain yield of cereals including wheat with elevated CO_2_. In the latter meta-analysis, grain yield also increased by 24.8% with increased temperature and by 70.5% with drought stress^50^. Grain Zn and Fe concentrations significantly decreased with elevated CO_2_ but did no significantly change with elevated temperature and drought stress^50^. According to two meta-analyses^47,51^, elevated CO_2_ resulted in 3–12% reductions in grain Zn and Fe concentrations in wheat relative to ambient CO_2_ levels.

Existing evidence also suggests a decrease in grain protein concentrations with elevated CO_2_ but increases with increased temperature and drought stress^46–48,50,52^. According to two separate meta-analyses^46,50^, elevated CO_2_ resulted in a 5–9% reduction in protein concentrations, a phenomenon known as the dilution effect^47^. Generally, CO_2_ enrichment has been shown to slow shoot amino acid production, leading to lower wheat grain protein concentrations^48^. By contrast, the same meta-analysis^50^ has shown a 10.4% increase in grain protein concentrations with increased temperature and a 12.4% increase with drought stress. A study in Mexico also found a 32% increase in grain protein concentrations with increased heat stress and a 37% increase with water stress in wheat^52^. Taken together, these findings provide sufficient evidence for decreases in grain Zn, Fe and protein concentrations with elevated CO_2_ but increases with elevated temperature and drought stress. This highlights uncertainties about the overall effect of future climate scenarios. This indicates the need for investment in research and climate modelling to fill the knowledge gap.

The results of the random effects model indicated that grain yield and grain Zn, Fe and protein concentrations are controlled by complex interactions among variables. The contemporary literature does not provide clear explanations for the differences in the relative importance of explanatory variables or the interactions between variables. In the case of individual variables, our results in the meta-analysis section provide some explanation. For example, the role of applied P and availability of P in the soil in determining grain Zn concentrations has been shown in our meta-analysis and in other studies^37,39^. Such explanations do not exist for the other explanatory variables. This indicates a gap in our understanding of interactions between grain nutrient concentrations and explanatory variables, and hence the need for future agronomic research to tease out these interactions. Overall, the results above indicate the role of climatic, geographical and soil variables and fertilizer management in determining grain yield and Zn, Fe and protein concentrations in wheat.

## Methods

### Scope of this analysis

The scope of this work is global, encompassing all wheat-growing regions, species and cultivars, but limited to studies published before May 2024, when this paper was prepared. Wheat was selected for this analysis owing to its pivotal role in the emergence and development of agriculture^20^, and its status as the second most consumed cereal after rice and the most widely cultivated crop globally^13^. However, wheat is more susceptible to Zn deficiency compared with other major cereals. Zn was chosen as the focus of this analysis because approximately 50% of global soils used for cereal cultivation are deficient in plant-available Zn^6^, a condition strongly linked with the severity of Zn deficiency in humans^5^. Grain protein concentration was included in this analysis because it is a key determinant of wheat flour properties and end-use quality^25^. Grain phytate levels were also analysed because phytate reduces the bioavailability of both Zn and Fe in food products. Phytate is an indigestible compound that inhibits Zn and Fe absorption in humans^3^, making it a critical factor in evaluating the overall nutritional quality of wheat.

### Literature search and inclusion and exclusion criteria

The literature search (Supplementary Fig. 1) followed the Preferred Reporting Items for Systematic Reviews and Meta-Analyses for Reporting Literature Searches (PRISMA-S) guidelines. A comprehensive search was conducted using Web of Science, CAB Direct, Scopus, PubMed and Google Scholar from September 2023 to February 2024. The search terms for Web of Science, CAB Direct, Scopus and PubMed included (“Micronutrient” OR “Zinc Application” OR “Iron Application” OR “Ferti*”) AND (“Wheat” AND “Grain Zinc” OR “Grain Iron” AND “Grain Yield” OR “Produ*”). In Google Scholar, the search string used was “Bread Wheat” OR “Durum Wheat” AND “Grain Zn” OR “Grain Fe” OR “Mineral” AND “Grain Protein”. Boolean operators “AND” and “OR” were used to combine the keywords, and asterisks (*) were included to account for multiple keyword variants. An additional literature search was also conducted on G × E interactions and heritability to understand the genetic and environmental influence on grain Zn, Fe and protein concentrations in wheat. Details of the search and compilation are summarized in Supplementary Methods. Retrieved publications were catalogued following the PRISMA-S checklist.

Publications were included in the analysis if they met the following inclusion criteria: (1) the study reported results from randomized and controlled field experiment or well-designed surveys in which grain mineral concentrations were expressed on a dry matter basis; (2) the study involved wheat genotypes or cultivars managed under uniform field management practices; (3) data on whole-grain Zn, Fe, protein and phytate concentrations and yield could be extracted based on specific agronomic management practices; (4) the study was published in English; and (5) treatments were replicated. Studies were excluded if they (1) lacked search string keywords in the title or abstract, (2) were conducted in greenhouse or pot experiments, (3) were review articles, (4) were published in non-peer-reviewed journals or (5) presented results inconsistent with the treatment design. On the basis of these criteria, 243 studies (Supplementary Fig. 1 and Supplementary Table 1) from 41 countries were selected for analysis (Supplementary Fig. 2). The entire dataset was used to define the global distributions of grain Zn, Fe, protein and phytate concentrations on a dry matter basis. A subset of studies that included specific treatments with their corresponding controls was selected for the meta-analysis (Supplementary Fig. 1). The following steps ensured the quality of publications included in the analysis: (1) only publications from peer-reviewed journals were considered; (2) each selected publication underwent full-text review before data extraction; (3) the final list of selected studies was independently evaluated by three reviewers, who provided a final decision on inclusion; and (4) any disagreements among reviewers were resolved through mutual consensus.

### Data extraction

Data from tables were recorded directly, while data from figures, such as bar charts and graphs, were extracted using WebPlotDigitizer (https://automeris.io/WebPlotDigitizer/). Site-specific characteristics, including site name; geographic coordinates; study year; seasonal rainfall; soil clay, sand and silt content; soil pH; SOC; total nitrogen, available P and K, and soil Zn and Fe concentrations, were extracted from each study. Information on crop management practices, including irrigation, inoculation and application rates of N, P, K, Zn and Fe, along with grain yield and grain concentrations of Zn, Fe, protein and phytate, was also recorded. Details on wheat variety, release date and growth habit were retrieved from Wheat Atlas (https://archive.wheat.org/wheat-useful-links/) and cross-verified with national documentation. Missing soil data were obtained from Soil Grids (https://soilgrids.org/) using the GPS coordinates of experimental sites. Soil type data, based on World Reference Base, were extracted from SoilGrids. All data were organized in Microsoft Excel for subsequent visualization and statistical analysis. The complete dataset used in this analysis is publicly available via the CGIAR MEL data repository at https://hdl.handle.net/20.500.11766/70237 (A global dataset on agronomic and genetic biofortification of wheat for zinc, iron and protein concentration).

### Data processing for analysis

To facilitate the meta-analyses, continuous explanatory variables were converted into categories, and dummy variables were created. For instance, soil texture classes were generated based on clay, sand and silt concentrations, resulting in three categories: sandy (<20% clay), loam (20–32% clay) and clayey (> 32% clay). Soils were also classified according to their parent materials as calcareous, silicic or mafic, following Gray et al.^53^. Calcareous parent materials include limestone, dolomite, shale and sands containing more than 50% CaCO₃ or MgCO₃. Silicic (acidic) parent materials are rocks with >68% silica (Si), while intermediate parent materials contain 52–68% Si^53^. Mafic (basic) parent materials are those with low silica content (45–52% Si)^53^.

Soil organic matter data were converted to SOC by dividing soil organic matter values by 1.75. The resulting SOC values were categorized into two groups: low (SOC < 1%) and medium to high (SOC > 1%). In cases in which studies used different soil extraction methods for soil pH, available P and cations, and reported results in various units, these were converted to consistent units before meta-analysis. For example, all soil pH values were standardized to pH in H_2_O. Soil pH data were grouped into three categories: acidic (pH < 6.5), neutral (pH 6.5–7.5) and alkaline (pH > 7.5). Similarly, *P* values extracted by different methods were converted to Olsen P equivalents using regression equations. Olsen P (bicarbonate extraction) was chosen as the preferred indicator of soil P status owing to its widespread use^54^. Olsen P values were categorized into three groups: low (<15 mg kg^−1^), medium (15–22 mg kg^−1^) and high (>22 mg kg^−1^). Climate zones were classified using GPS coordinates of study sites: tropical (within 23.5° N and S), subtropical (23.5–35° N and S), temperate (35–50° N and S) and boreal (>50° N).

### Statistical analysis

#### Empirical distributions of wheat grain Zn, Fe, protein and phytate

Empirical distributions were derived from all datasets of field experiments and surveys. Survey data were not handled differently from experimental data as they reported sample means. Of the 243 studies, 218, 124, 112 and 37 reported grain Zn, Fe, protein and phytate concentrations, respectively (Supplementary Fig. 1). These data were used to establish the empirical distribution of nutrient concentrations in whole grain. Before the distributions were analysed, outliers were identified using the extreme studentized deviate test using the Paleontological Statistics (PAST) software. Histograms of wheat grain Zn, Fe and protein concentrations were generated, with the number of bins optimized using the zero-stage rule. Gaussian kernel density and normal distribution curves were added to the histograms. The probability (*ϕ*) of exceeding the target values for Zn (38 mg kg^−1^) and Fe (59 mg kg^−1^) in wheat grain, as set by HarvestPlus^24^, was estimated using the cumulative frequency distributions. The expected values of grain Zn and Fe concentrations were determined using the medians and the lower and upper quantiles (Q1 and Q3). Uncertainty around the median values was represented by 95% confidence intervals (CIs), which were estimated using bias-corrected and accelerated (BCa) bootstrapping with 9,999 replicates in the PAST software.

#### G × E interactions and heritability of traits

The effects of G × E interactions on grain yield and grain Zn, Fe and protein concentrations in wheat are not well understood owing to limited studies. Understanding heritability is essential to determine whether Zn, Fe and protein concentrations can be improved in bread wheat through traditional breeding methods. To address this, studies quantifying G × E interactions and heritability of Zn, Fe and protein concentrations were reviewed, and the variance explained by the genotype, environment and G × E interactions along with broad-sense heritability index (*H*^2^) values was compiled (Supplementary Table 4).

#### Correlation and regression analyses

Correlations between grain yield and grain Zn, Fe, protein and phytate concentrations were examined to assess associations among these variables. Correlation analysis was performed for each study and then across all studies. The studies were grouped into two sets: (1) those examining variations in grain yield and grain Zn, Fe and protein concentrations with germplasm and (2) those assessing variations in grain yield and nutrient concentrations with fertilization. Data were available for correlation analysis from 42 and 65 studies in the first and second sets, respectively. The first set should inform whether simultaneous improvement of grain Zn, Fe or protein concentrations with grain yield is possible through traditional breeding. The second set should inform whether simultaneous improvement of grain Zn, Fe or protein concentrations with grain yield is possible with Zn and/or Fe fertilization. In all cases, correlation analysis was limited to studies reporting at least 10 means to accurately estimate the Pearson correlation coefficient. Furthermore, correlation analysis was performed between application rates of N, P, K, Zn, soil total N, Olsen P, available K, soil Zn, soil Fe and grain Zn and Fe concentrations, as well as response ratios, to explore relationships. For response ratios, analysis was limited to sites where Zn was applied. All correlation analyses were performed using data transformed to their natural logarithms.

LOESS, a non-parametric regression method, was used to identify trends in response ratios with N, P and Zn application rates. LOESS was chosen over linear or nonlinear regression because parametric relationships could not be established in exploratory analyses. LOESS regression was performed using the PAST software.

#### Trends in grain yield and grain Zn, Fe and protein concentrations

For this analysis, bread and durum wheat varieties were classified by year of release, and distributions and average values were determined. A total of 1,218 bread wheat and 64 durum wheat, released between 1908 and 2022, were included. To simplify analysis, release years were grouped into four 20-year periods: pre-1960, 1960–1980, 1981–2000 and 2001–2022. This grouping was applied only to varieties with verified release years. The trends in distribution were visualized using box and violin plots. Statistical inferences were based on medians and their 95% CIs, estimated using BCa bootstrapping with 9,999 replicates.

#### Effect of genetic biofortification

The analysis of grain yield and nutrient concentrations was performed focusing on studies comparing genetically biofortified cultivars with non-biofortified varieties. This analysis was limited to Zn-biofortified cultivars owing to insufficient data for Fe-biofortified wheat cultivars.

#### Meta-analysis

The meta-analysis was performed on a subset of field experiment data in which a reliable ‘control’ could be identified. For all target variables, the ‘control’ was defined as the treatment receiving the recommended inorganic NPK fertilizer, or NP where K was not recommended. NPK fertilizer was chosen as the control because Zn, Fe and other micronutrients are applied with NPK, making it a suitable baseline for comparing gains in grain yield and nutrient concentrations from micronutrient inputs. Meta-analysis of the tillage and organic inputs was excluded owing to insufficient sample sizes. Similarly, emmer and spelt wheat were excluded from the meta-analyses for the same reason. The meta-analysis primarily focused on effect sizes for agronomic, soil and climate variables in bread wheat, with less emphasis on durum wheat. A similar analysis could not be conducted for spelt and emmer wheat owing to limited sample sizes. Supplementary Table 6 summarizes the total sample sizes available for the meta-analysis of target variables. The RR—calculated as the ratio of the treatment value to the corresponding control—was used as the effect size metric for the meta-analysis of target variables, including grain yield and grain Zn, Fe, protein and phytate concentrations. To normalize the RR, its natural logarithm (ln(RR)) was calculated as follows:

$$\mathrm{ln}(\mathrm{RR})=\mathrm{ln}\left(\frac{T}{C}\right)$$

where *T* and *C* represent the values of the target variable from the treatment and control, respectively. After analysis, ln(RR) and its 95% CIs were back-transformed into the arithmetic domain. Throughout the paper, effect sizes expressed as percentages were used for inference because percentages are more accessible to non-technical readers. For this purpose, ln(RR) or RR was re-expressed as percentage change as follows^45^:

$$\mathrm{Change}\,( \% )=100\times \left({e}^{\mathrm{ln}(\mathrm{RR})}-1\right)\,\mathrm{or}\,100\times \left(\mathrm{RR}-1\right)$$

Before the meta-analysis, publication bias was assessed by visualizing normal quantile–quantile (*Q*–*Q*) plots of the log-transformed response ratios, that is, ln(RR) (Supplementary Fig. 5). A linear mixed-effects modelling framework was used to estimate variation in effect sizes based on genotypic variables, agronomic practices, soil properties and climate variables, treated as categorical variables. Genotypic variables included wheat species (bread versus durum), seasonality (facultative, spring or winter) and genetic biofortification status (biofortified versus non-biofortified). Cultivars were stratified into biofortified and non-biofortified to assess changes in grain yield and grain Zn, Fe and protein concentrations with genetic biofortification. To ensure comparability, a subset of studies comparing non-biofortified and fortified cultivars grown under identical agronomic conditions was analysed.

Agronomic practices included irrigation (irrigated versus rain-fed), organic input application (applied versus none), Zn or Fe application (applied versus none) and the mode of Zn and Fe application (foliar, seed, soil) in combination with NP (±K) fertilizer (Supplementary Table 6). The analysis primarily focused on treatments with Zn alone or Zn combined with Fe, referred to as Zn (±Fe) fertilization. Meta-analysis was not conducted for tillage, as most studies used conventional tillage. The limited sample sizes also prevented analysis of organic inputs owing to fewer studies reporting grain yield (*n* = 18) and grain Zn concentration (*n* = 14) with organic inputs.

Soil variables included parent material (calcareous, mafic, intermediate, silicic), soil type (World Reference Base reference soil groups), soil texture (coarse, fine, medium), soil pH (acidic, alkaline, neutral), Olsen P levels (high, medium, low) and SOC levels (high, medium, low). Climate variables included climate zone and mean annual rainfall. Categorical variables were entered into the model as fixed effects, while study (each published paper) was treated as a random effect to account for clustering. Owing to limited data, interaction effects among variables were not explored.

The data were highly unbalanced because of varying studies and observations (sample sizes) for each categorical variable or treatment (Supplementary Table 6). To address this, certain treatments were grouped to form homogeneous categories, reducing data fragmentation and minimizing artifacts from small sample sizes. For instance, all treatments involving Zn, Fe or Zn + Fe (indicated by ±) were treated as homogeneous groups, distinct from other treatments (Supplementary Table 6). Subset analyses were performed on these grouped data. The Kenward–Roger method was used to approximate degrees of freedom and correct standard error estimates, addressing discrepancies in sample sizes. In all results, uncertainty around the estimated values is represented by 95% CIs, calculated using the mixed-effects models.

#### RF analysis

We applied RF ensemble modelling, a machine learning algorithm, to identify the environmental and management variables influencing grain yield and grain Zn, Fe and protein concentrations. We chose the RF algorithm because it outperforms other methods in handling high-dimensional data, outliers and missing values, common within our dataset. The ability of RF to generate feature importance scores enabled the identification of the most influential variables affecting yield and nutrient concentrations. The RF algorithm creates and merges multiple decision trees to form robust predictive models. A set of 21 predictor variables was used for all response variables, including country, variety release year, aridity index (AI), in-season rainfall, minimum temperature (*T*_min_) and maximum temperature (*T*_max_) during the growing season, production environment (irrigated versus rain-fed), SOC (%), clay content (%), sand content (%), soil pH (H_2_O), available P (Olsen), soil-available Zn (mg kg^−1^), Fe content in the soil (mg kg^−1^), exchangeable K, and N, P and K fertilizer rates. We used the coefficient of determination (*R*^2^) and the cross-validation root mean squared error to assess model accuracy. To estimate variable importance, we compared predictions from training data with observations not used in tree construction, that is, the out-of-bag samples. We used the ‘feature_importances’ function of the RF model to assess the relative importance of each variable. To estimate the importance of each variable, we compared predictions based on the data used for the training of the model with observations not used in the creation of the trees (that is, the out-of-bag samples). We performed all analyses using R software.

## Supplementary information

Supplementary Information Supplementary Figs. 1–13, Tables 1–9, methodology and code availability.

## Source data

Source Data Fig. 1 Source data for Fig. 1a–d.

Source Data Fig. 2 Source data for Fig. 2a–h.

Source Data Fig. 3 Source data for Fig. 3a–d.

Source Data Fig. 4 Source data for Fig. 4a–d.

Source Data Fig. 5 Source data for Fig. 5a–d.

## Data Availability

The complete data used in this analysis are publicly available via the CGIAR MEL data repository at https://hdl.handle.net/20.500.11766/70237 (A Global Dataset on Agronomic and Genetic Biofortification of Wheat for Zinc, Iron and Protein Concentration). Summary data not in the main text have been provided in Supplementary Information. Source data are provided with this paper.

## Code availability

We have uploaded the custom codes we used as supplementary information. The R code used for RF modelling and evaluation and SAS code for running linear mixed-effects models are provided in Supplementary Code.
