# Supplementary material for: Grain zinc, iron and protein concentrations of contemporary wheat cultivars fall short of targets for human health
Source: Nat Food. 2026 Mar 9;7(3):283–95. doi: 10.1038/s43016-026-01314-3 (PMC13021525; doi:10.1038/s43016-026-01314-3)
Supplement: Supplementary file 1 — Supplementary Figs. 1–13, Tables 1–9, methodology and code availability. [file 43016_2026_1314_MOESM1_ESM.pdf]

nature food

Supplementary information https://doi.org/10.1038/s43016-026-01314-3

Grain zinc, iron and protein concentrations of contemporary wheat cultivars fall short of targets for human health

In the format provided by the authors and unedited

Supplementary Figures

Figure S1

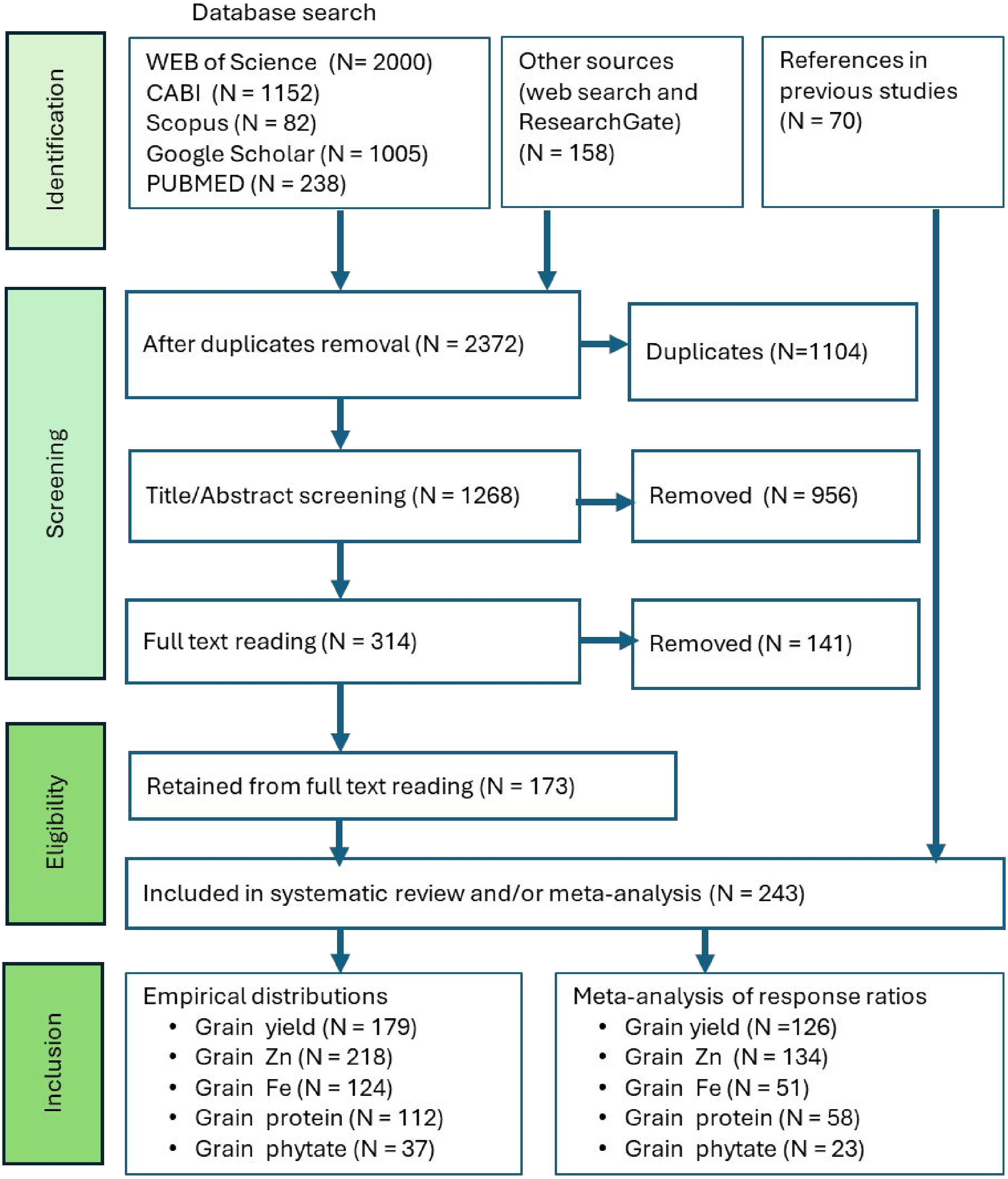

Figure S1. Flow diagram of the literature search, the number of studies (N) found during the search, number of studies excluded at each stage and those retained for review and meta-analysis.

Figure S2

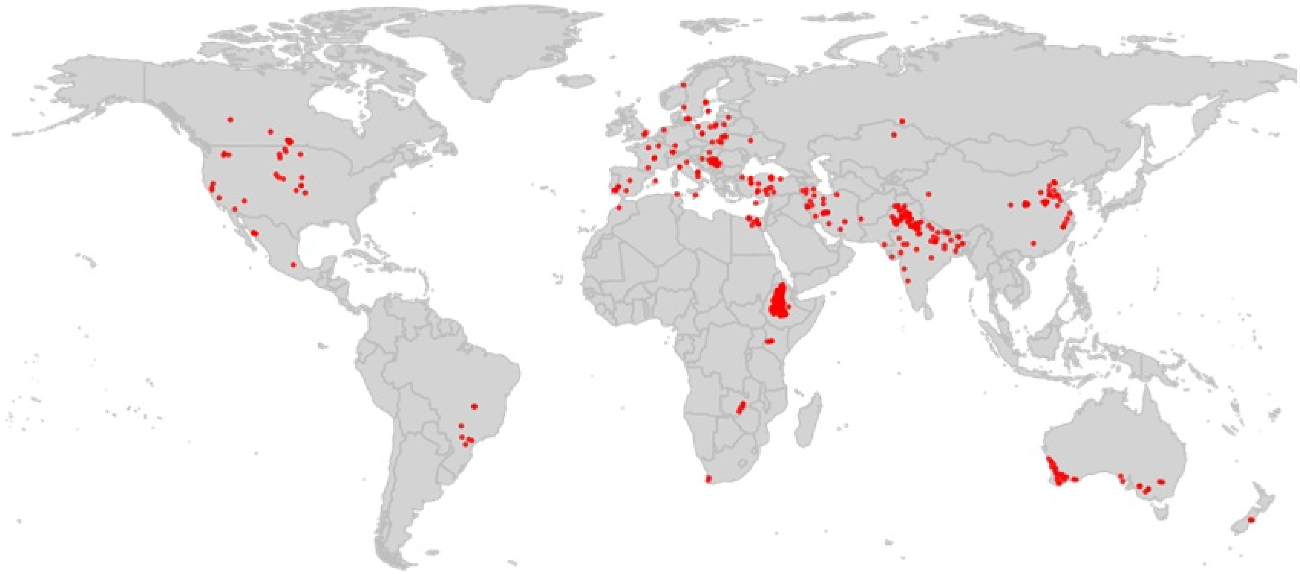

Figure S2. Maps of the studies included in this meta-analysis. This map was created by the authors in R (version 4.4.0, 2024-04-24) using the following packages: ggplot2 (v3.5.2) for visualization, maps (v3.4.1) for geographic boundaries, and dplyr (v1.1.4) for data merging. Country polygons from the world dataset were joined with biofortification crop data via country name matching. The map projection was truncated at 60°S to exclude Antarctica, preserving all major agricultural zones.

Figure S3

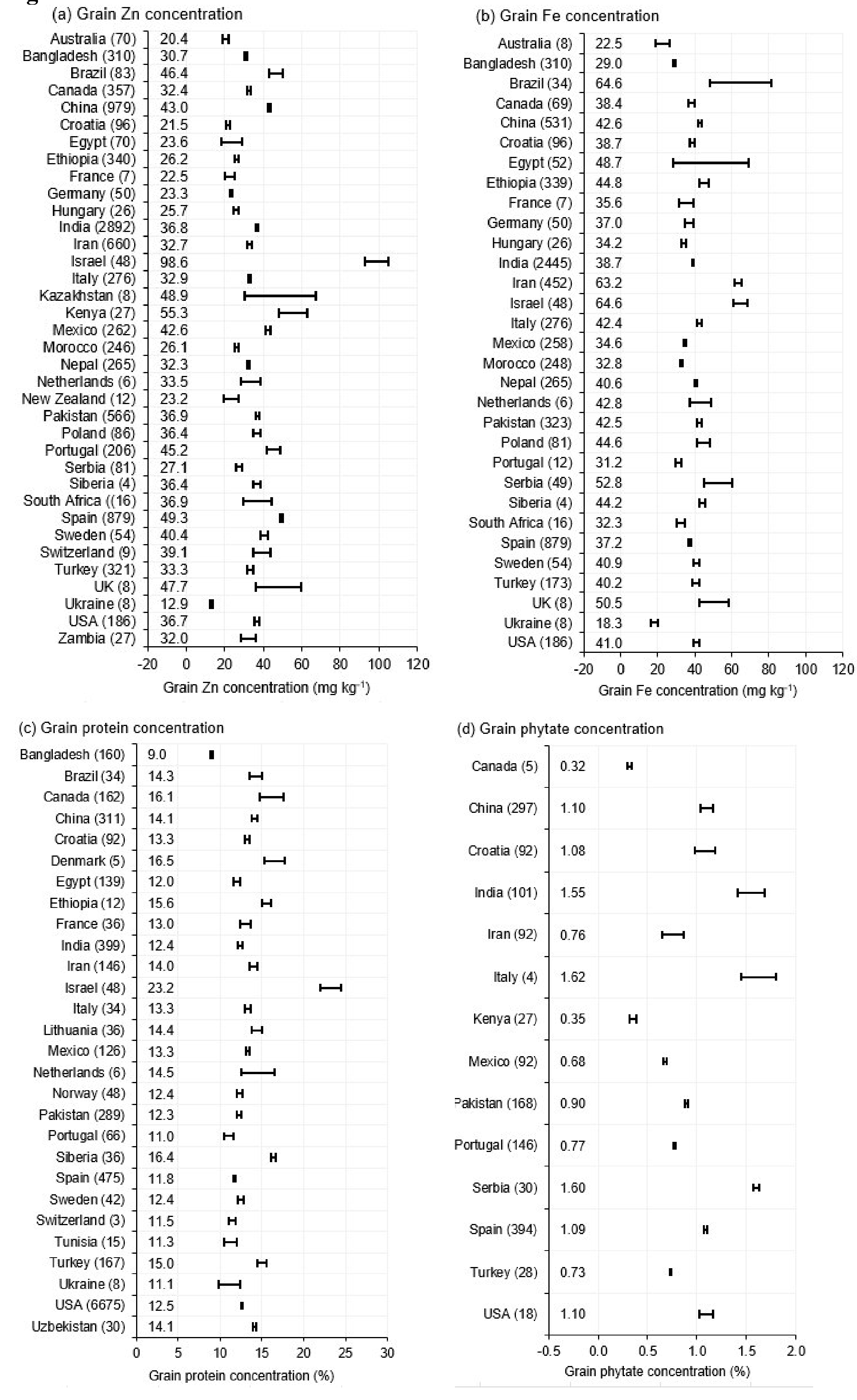

Figure S3. The country-wise distribution of grain Zn, Fe, protein and phytate concentrations across wheat cultivars. Figures in parentheses after country names represent the total sample size available for each country. Values of Zn, Fe and protein were larger than in all other countries because they were entirely from durum and emmer wheat.

Figure S4

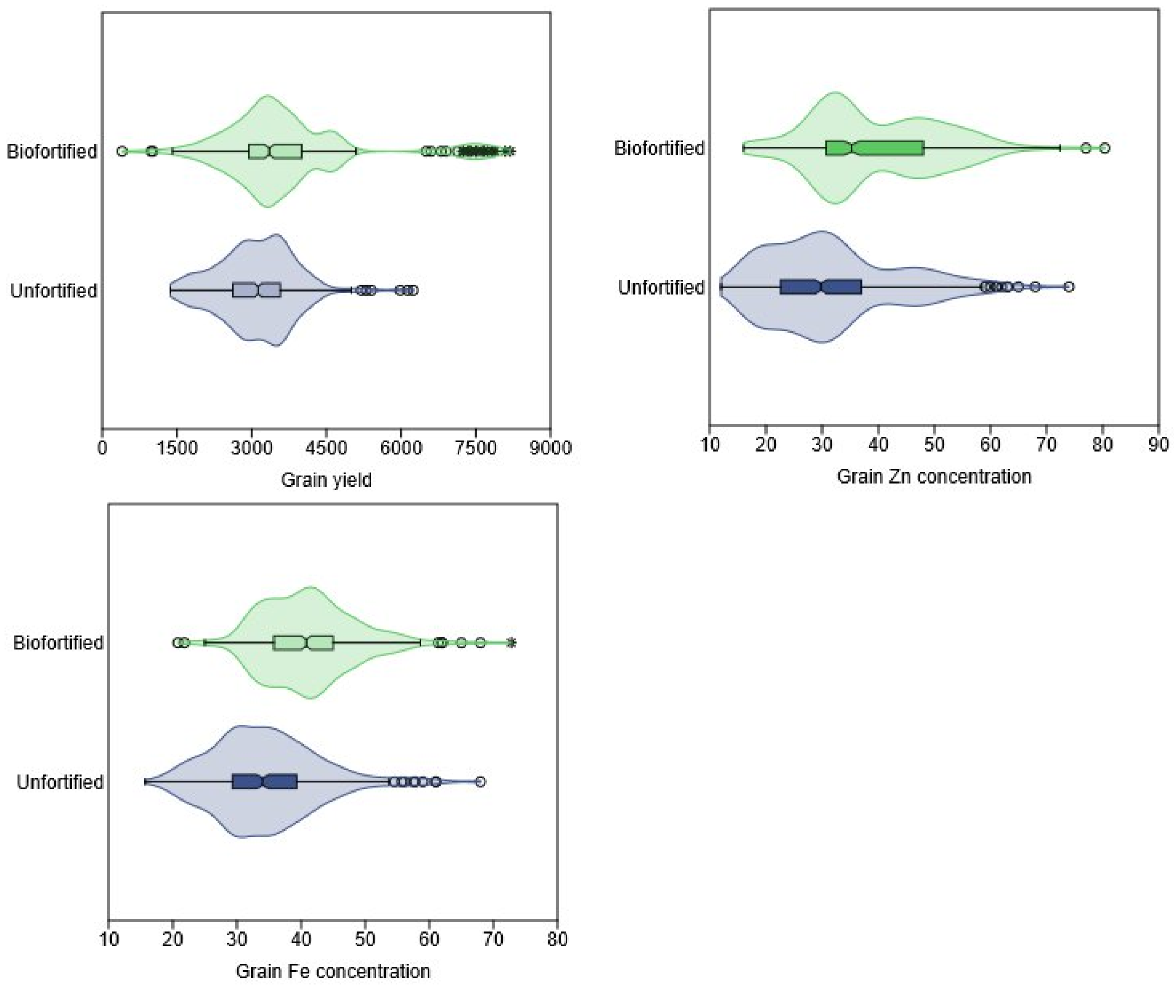

	Mann-Whitney test of equality of medians	Kolmogorov-Smirnov test for equality of distributions	Anderson-Darling test for equality of distributions
Grain yield (kg ha^{-1})	P< 0.0001	P< 0.001	P< 0.001
Gran Zn (mg kg^{-1})	P< 0.0001	P< 0.001	P< 0.001
Grain Fe (mg kg^{-1})	P< 0.0001	P< 0.001	P< 0.001

P values were generated using Monte Carlo permutation

Figure S4. Comparison of unfortified and genetically fortified bread wheat cultivars in terms of median values and the distribution of grain yield, grain Zn and Fe concentrations (mg kg^{-1})

Figure S5

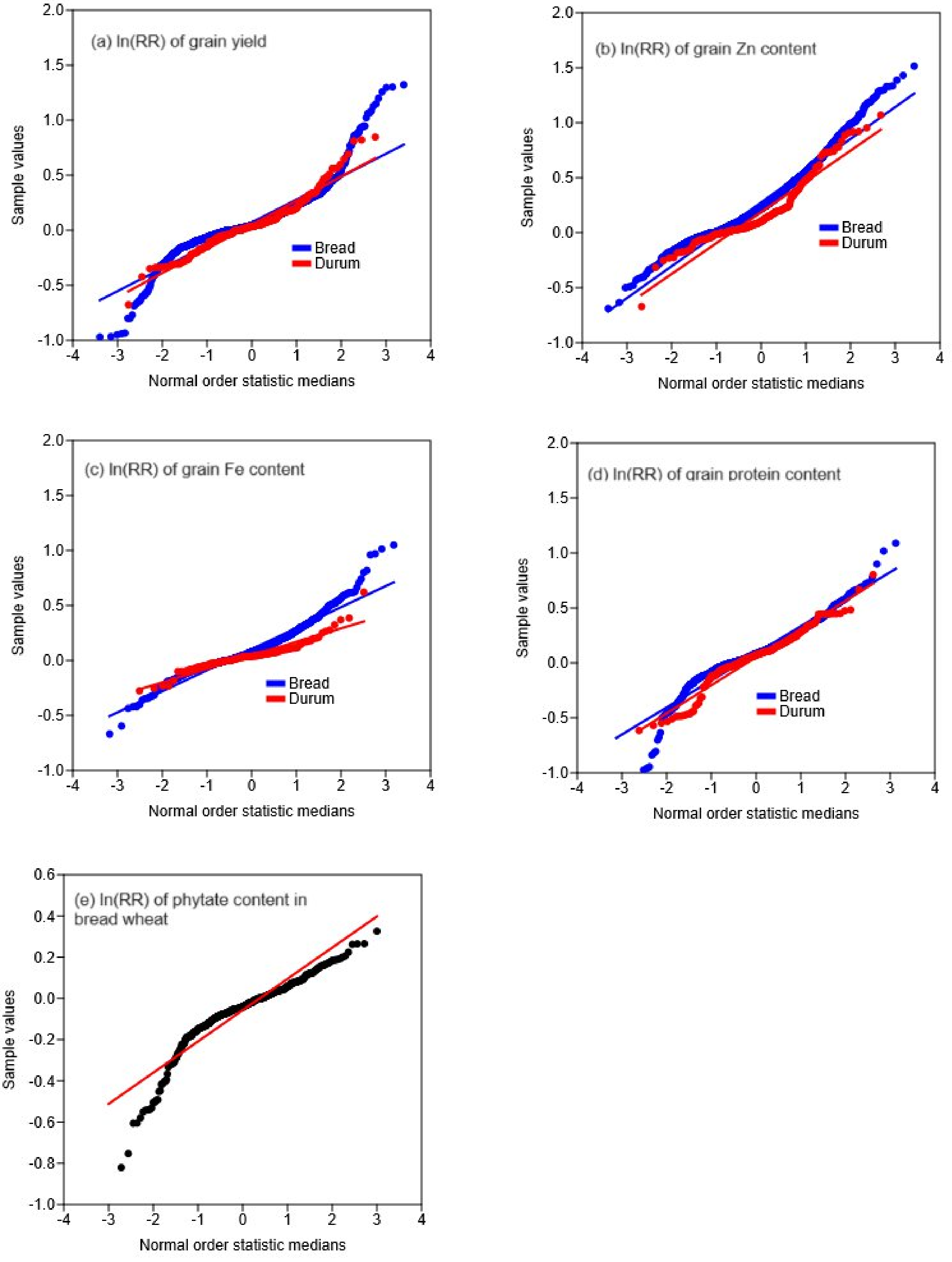

Figure S5. Normal Q-Q plots of ln(RR) of response ratios of grain yield (a), grain Zn (b), grain Fe (c), protein (d) and phytate concentrations in studies chosen for meta-analysis.

Figure S6

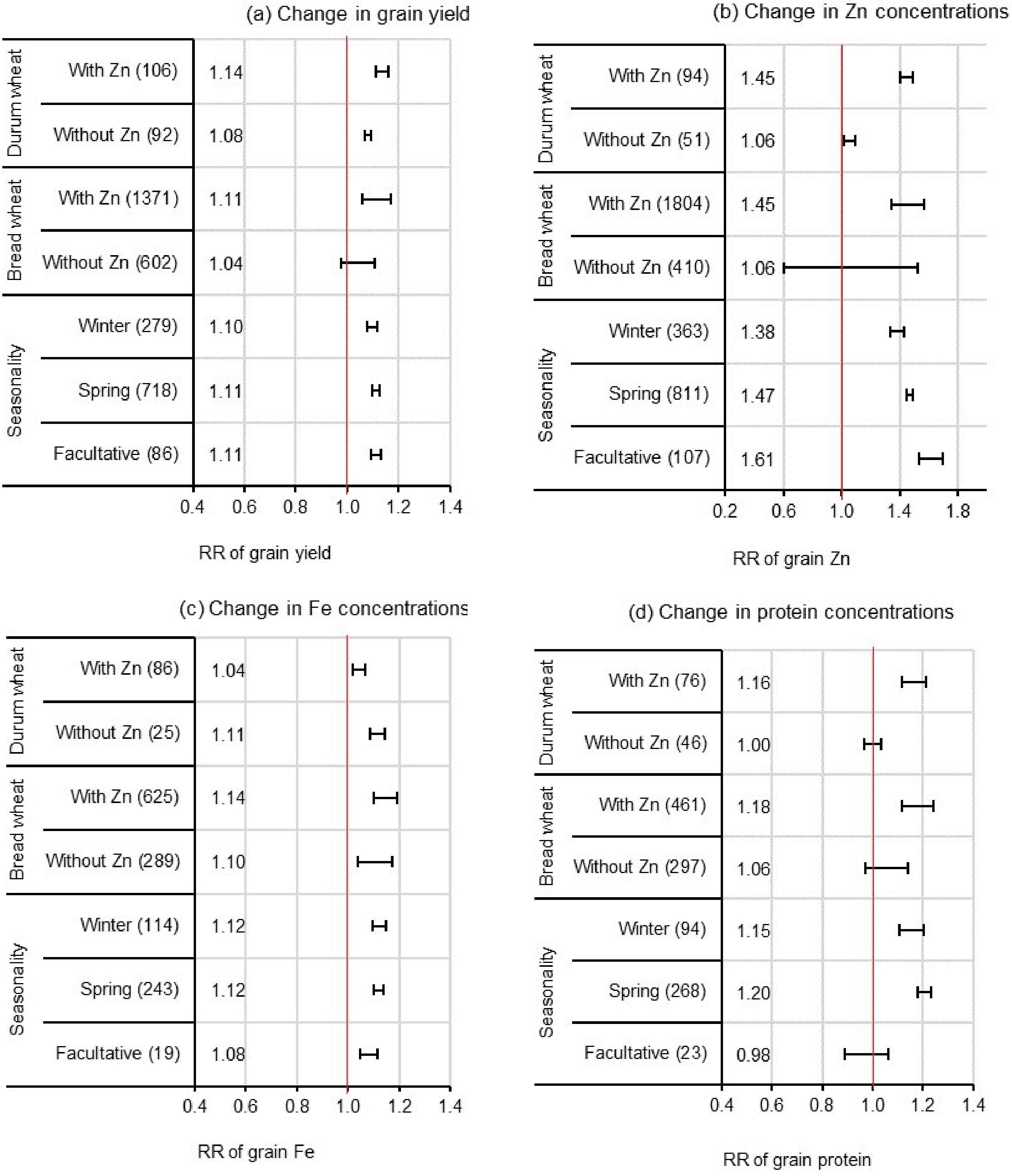

Figure S6. Variations in response ratios of grain yield, grain Zn, Fe and protein concentrations of durum and bread wheat with (With Z) and without (Without) Zn application and with seasonality in bread wheat cultivars. When the CIs encompasses RR = 1, the Zn (+/-Fe) treatment is not significantly different from the NPK control. Figures in in parentheses in front of treatments represent the total sample sizes, while those on the left sides of the horizontal bars represent the marginal means.

Figure S7

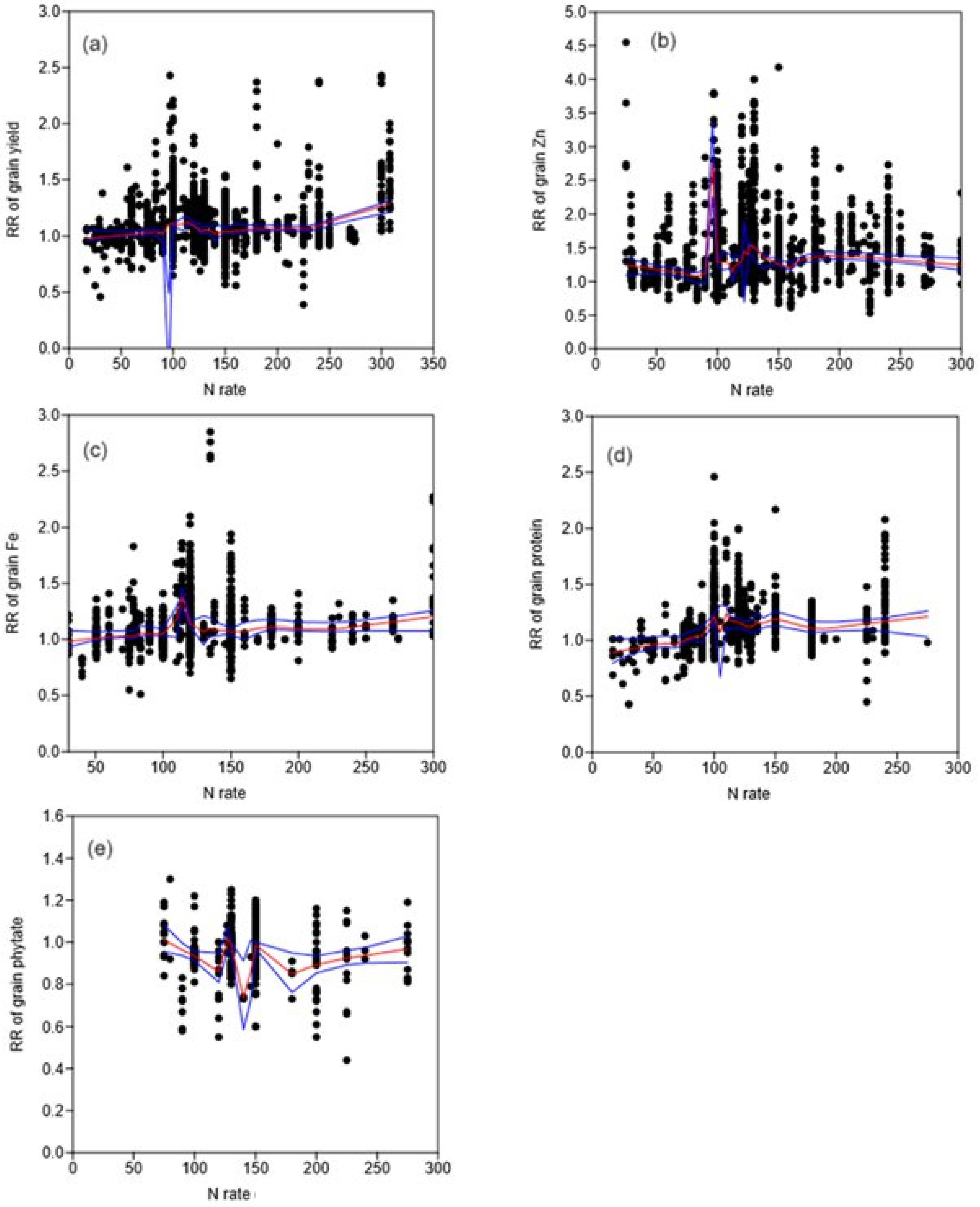

Figure S7. LOESS regression showing trends in response ratios of grain yield (a), grain Zn (b), grain Fe (c), protein (d) and phytate (e) concentrations of bread wheat with increasing rates (kg ha^{-1}) of soil applied N. The blue lines represent the 95% confidence limits of trend lines.

Figure S8

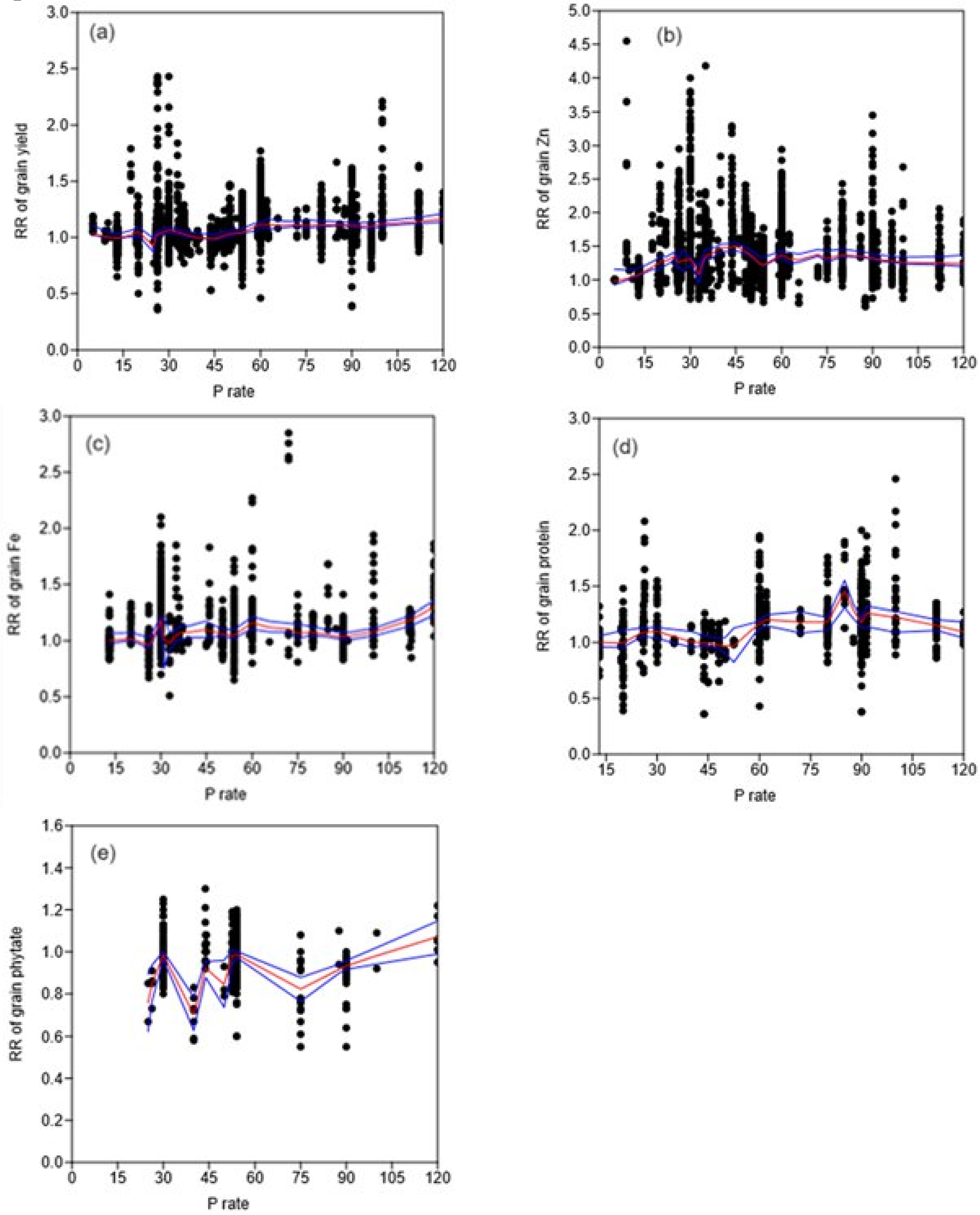

Figure S8. LOESS regression showing trends in response ratios of grain yield (a), grain Zn (b), grain Fe (c), protein (d) and phytate (e) concentrations of bread wheat with increasing rates (kg ha^{-1}) of soil applied P. The blue lines represent the 95% confidence limits of trend lines.

Figure S9

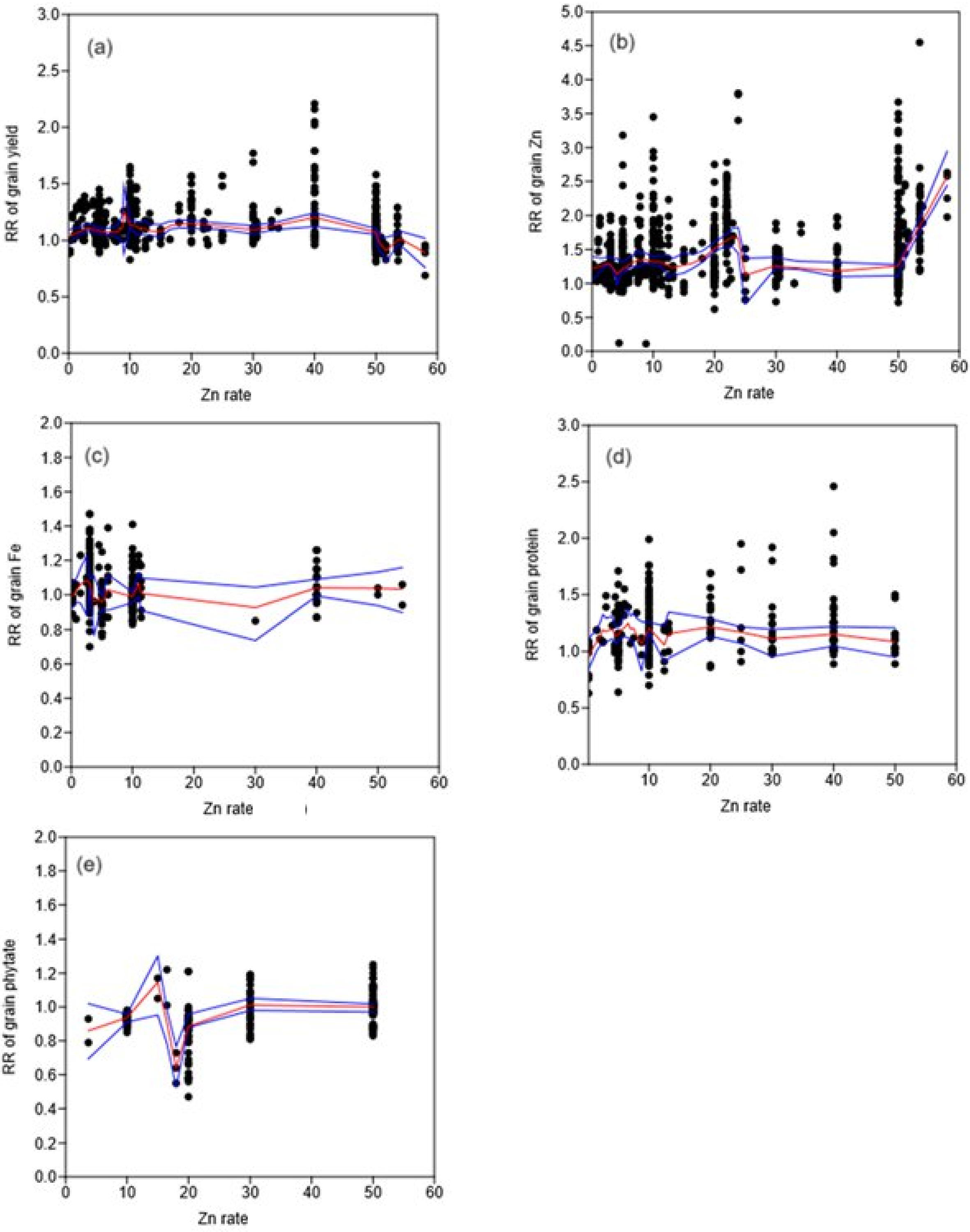

Figure S9. LOESS plots showing trends in response ratios of grain yield (a), grain Zn (b), grain Fe (c), protein (d) and phytate (e) concentrations of bread wheat with increasing rates (kg ha^{-1}) of soil applied zinc. The blue lines represent the 95% confidence limits of trend lines.

Figure S10

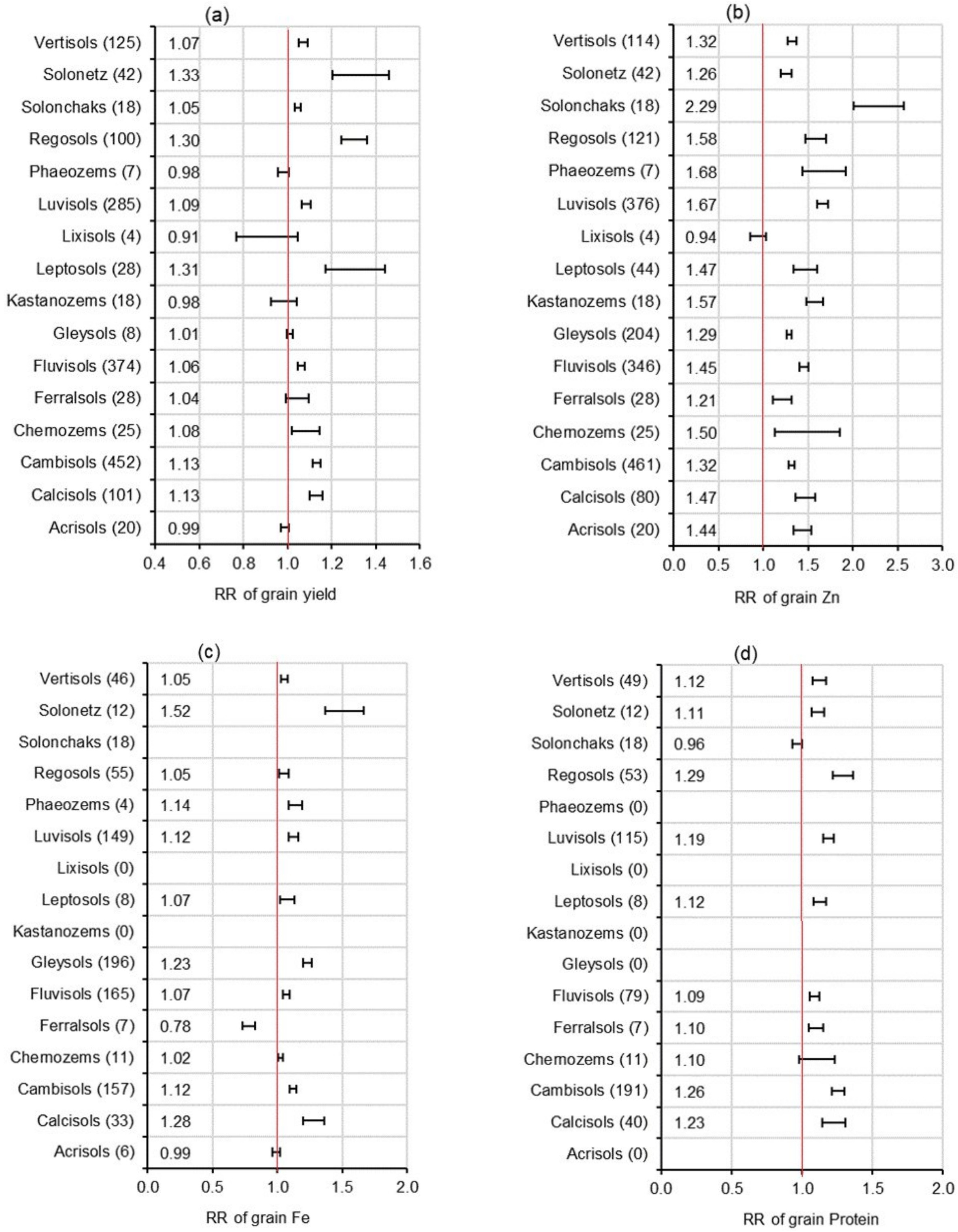

Figure S10. Variations in response ratios of grain yield, grain Zn, Fe and protein concentrations of bread wheat with soil type in treatments involving application of Zn (+/-Fe). The horizontal bars represent the 95% confidence intervals (CIs). When the CIs encompasses RR = 1, the Zn (+/-Fe) treatment is not significantly different from the NPK control. Figures in in parentheses in front of the soil type represent the total sample sizes, while those on the left sides of the horizontal bars represent the marginal means.

Figure S11

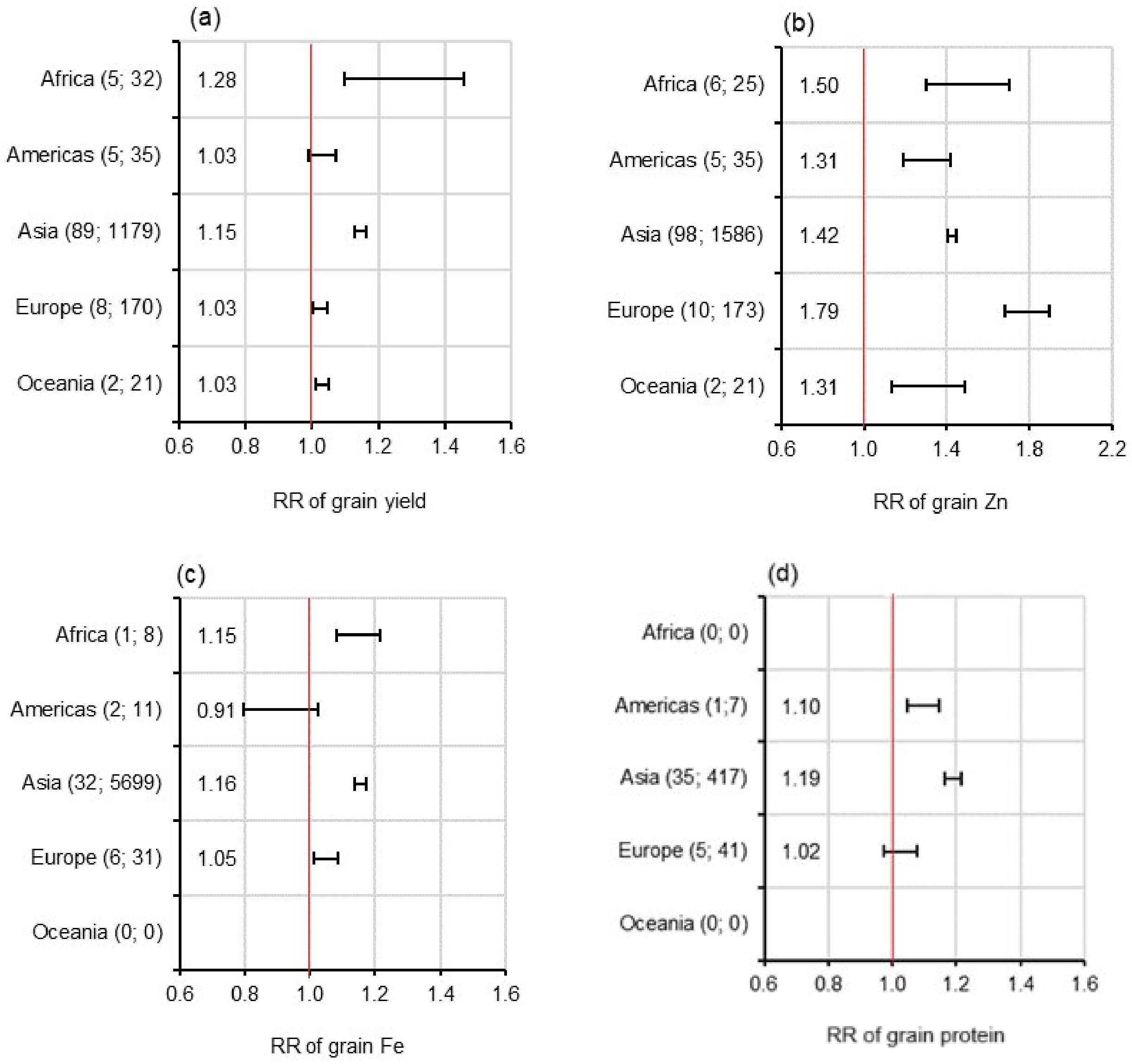

Figure S11. Variations in response ratios of grain yield, grain Zn, Fe and protein concentrations across continents with Zn (+/-Fe) fertilization of bread wheat cultivars. When the CIs encompasses RR = 1, the Zn (+/-Fe) treatment is not significantly different from the NPK control. Figures in in parentheses in front of continents represent the number of studies followed by the total sample sizes, while those on the left sides of the horizontal bars represent the marginal means.

Figure S12

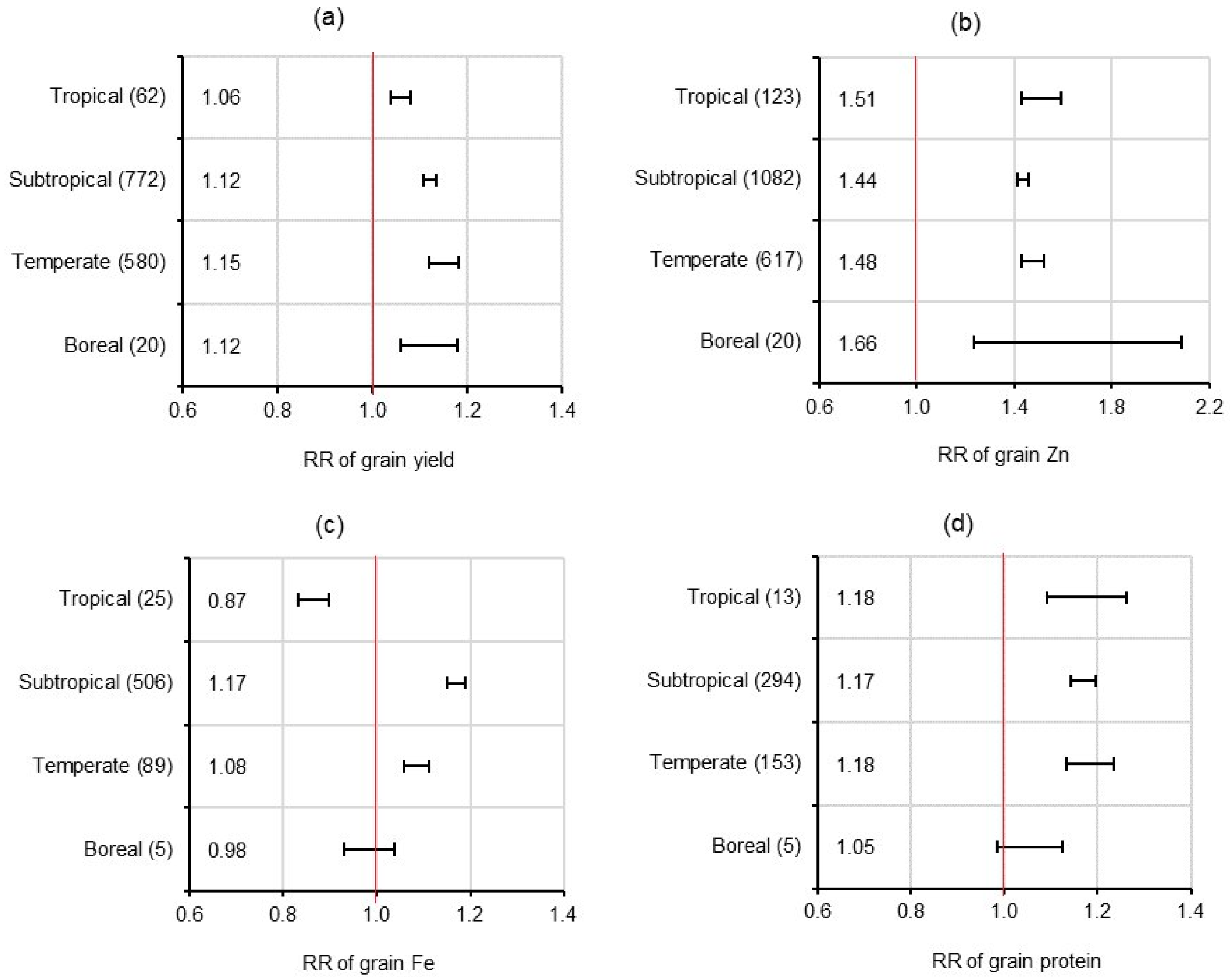

Figure S12. Variations in response ratios of grain yield (a), grain Zn (b), Fe (c) and protein (d) concentrations across climate zones with Zn (+/-Fe) fertilization of bread wheat cultivars. The horizontal bars represent the 95% confidence limits of means. When the 95% CL encompasses the red line (RR = 1), the treatment is not significantly different from the NPK control. Figures in in parentheses in front of treatments represent the total sample sizes, while those on the left sides of the horizontal bars represent the marginal means.

Figure S13

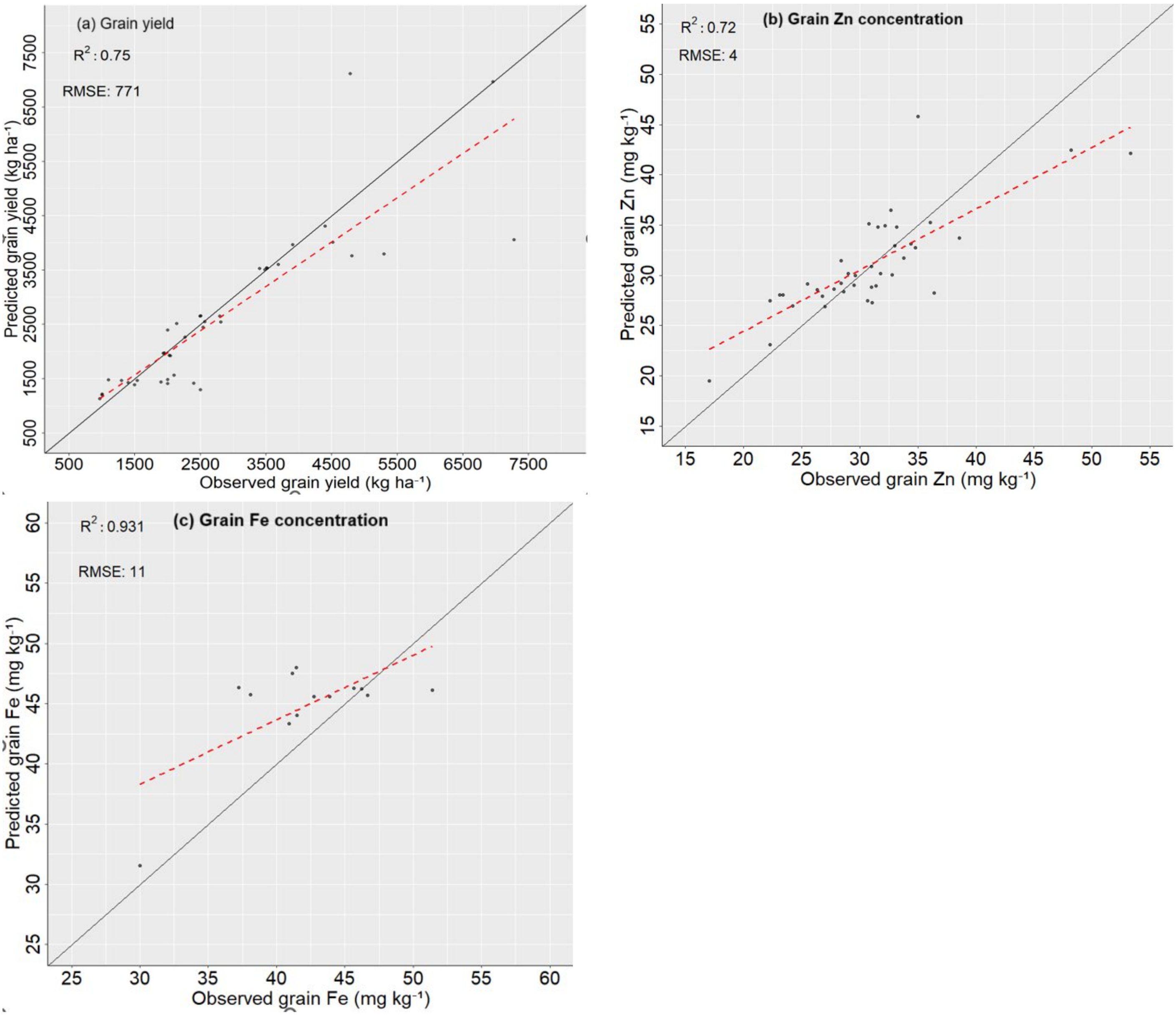

Figure S13. The relationship between the model prediction and observed values for grain yield (a), grain zinc (b) and grain iron concentration (c) from the random forest models. Black dots represent the observed data, red dotted lines represent the fitted lines for the regression of the predicted values on the observed values. Black solid line represents the 1:1 line. The coefficient of determination (R^2) and root mean square of error (RMSE) measure the agreement between the predicted and observed values.

Supplementary Tables

Table S1. List of studies

Table S1. Studies included in the review and meta-analysis

Study #	Complete citation
1	Abbas et. al.(2021) Zn Ferti-fortification of Wheat (Triticum Aestivum L.) Using Zinc Enriched Compost and Biochar in Rainfed Area. Communications in Soil Science and Plant Analysis 52(18). https://doi.org/10.1080/00103624.2021.1921189
2	Abdi et al. (2013) Residual and cumulative effect of fertilizer zinc applied in wheat-cotton production system in an irrigated aridisol. Plant Soil Environ 59
3	Abdoli et. al. (2014) Effects of foliar application of zinc sulfate at different phenological stages on yield formation and grain zinc content of bread wheat (cv. Kohdasht). Azarian Journal of Agriculture 1: 11-17
4	Ahsin et al. 2023 Genetic and agronomic zinc biofortification modify processing and nutritional quality of common wheat. Cereal Chemistry 100 (1). https://doi.org/10.1002/cche.10604
5	Akbar et al. (2023) Mycorrhizal inoculation enhanced tillering in field grown wheat, nutritional enrichment and soil properties. PeerJ http://doi.org/10.7717/peerj.15686
6	Akca & Taban (2024) Optimizing Grain Zinc Biofortification in Bread Wheat: Innovative Fertilization Strategies for Field Conditions. Journal of Soil Science and Plant Nutrition ??? https://doi.org/10.1007/s42729-024-01865-z
7	Akram et. al.(2017) Synergistic use of nitrogen and zinc to bio-fortify zinc in wheat grains. Eurosian Journal of Soil Science 6 (4) 319 – 326. Doi: 10.18393/ejss.306698
8	Ali et al. (2023) Foliar Spraying of Nano- Silicate and Nano-Zinc on Wheat (*Triticum aestivum* L.) Under Salt Stress Conditions Enhances Productivity and Reduces the Optimal Nitrogen Fertilizer Rate. Communications In Soil Science And Plant Analysis 54. https://doi.org/10.1080/00103624.2023.2240817
9	Ali et al. (2024) Enhancing Wheat Yield and Zinc Biofortification through Synergistic Action of Potent Zinc-Solubilizing Bacteria and Zinc Sulfate in Calcareous Soil. Agric Res ??? https://doi.org/10.1007/s40003-024-00750-6
10	Ali et. al.(2018) Micronutrient seed priming improves stand establishment, grain yield and biofortification of bread wheat Crop & Pasture Science, 69, 479–487. doi.org/10.1071/CP18042
11	Amir et al (2018) Estimating genetic variation and genetic parameters for grain iron, zinc and protein concentrations in bread wheat genotypes grown in Iran. Journal of Cereal Science 140: 16e23. https://doi.org/10.1016/j.jcs.2018.01.069
12	Andruszczak et al. (2020) Cultivar and foliar feeding of plants as factors determining the chemical composition of spelt (*Triticum aestivum* ssp. *Spelta* l.) grain. Applied Ecology And Environmental Research 18(2):2949-2958. DOI: http://dx.doi.org/10.15666/aeer/1802_29492958
13	Anwar et al. (2021) Mitigation of Drought Stress and Yield Improvement in Wheat by Zinc Foliar Spray Relates to Enhanced Water Use Efficiency and Zinc Contents. International Journal of Plant Production. https://doi.org/10.1007/s42106-021-00136-6
14	Ayadi et al. (2022) Variation of Grain Yield, Grain Protein Content and Nitrogen Use Efficiency Components under Different Nitrogen Rates in Mediterranean Durum Wheat Genotypes. Agriculture 12(7). https://doi.org/10.3390/agriculture12070916
15	Barut et.al.(2017) The Effect of Different Zinc Application Methods on Yield and Grain Zinc Concentration of Bread Wheat Varieties. Turkish Journal of Agriculture - Food Science and Technology 5(8): 898-907. doi.org/10.24925/turjaf.v5i8.898-907.122
16	Basshi & Nahapetian (1977) Differences in Concentrations and Interrelationships of Phytate, Phosphorus, Magnesium, Calcium, Zinc, and Iron in Wheat Varieties Grown under Dryland and Irrigated Conditions. Journal of Agricultural and Food Chemistry. https://doi.org/10.1021/jf60213a054
17	Bedoussac & Justes (2008) The Efficiency Of Durum Wheat And Winter Pea Intercropping To Increase Wheat Grain Protein Content Depends On Nitrogen Availability And Wheat Cultivar 10th Congress of European Society for Agronomy, 15-19th September 2008, Bologna, Italy. https://oatao.univ-toulouse.fr/4073/

18 Bezabeh et al. (2022) Wheat (Triticum aestivum) production and grain quality resulting from compost application and rotation with faba bean Journal of Agriculture and Food Research. https://doi.org/10.1016/j.jafr.2022.100425

19 Bharti et. al. (2013) Improving nutritional quality of wheat through soil and foliar zinc application. Plant Soil Environ. 59(8): 348–352

20 Biel et al. (2021) Comparison of yield, chemical composition and farinograph properties of common and ancient wheat grains. European Food Research and Technology 247(6). https://doi.org/10.1007/s00217-021-03729-7

21 Bilgrami et al. (2017) Phytic acid, Iron and Zinc content in Wheat Ploidy levels and Amphiploids: the impact of Genotype and Planting seasons. Archives of Agronomy and Soil Science 64(3). http://dx.doi.org/10.1080/03650340.2017.1352085

22 Biswas et. al.(2015) Effect of Soil Zinc and Boron on the Yield and Uptake of Wheat in an Acid Soil of West Bengal, India. International Journal of Plant & Soil Science 6(4).

23 Bloom et al (2021) Wheat grain yield decreased over the past 35 years, but protein content did not change. Journal of Experimental Botany 72: 6811. https://doi.org/10.1093/jxb/erab343

24 Brankovic et al. (2015) Phytic acid, inorganic phosphorus, antioxidants in bread and durum wheat and their associations with agronomic traits. Agricultural and Food Science 24(3). https://doi.org/10.23986/afsci.49729

25 Buczek et al. (2017) Hybrid wheat response to topdressing and foliar application of nitrogen. Journal of Elementology 22. https://doi.org/10.5601/jelem.2016.21.2.1125

26 Buczek et al. (2020) Hybrid wheat yield and quality related to cultivation intensity and weather condition. Journal of Elementology 25. https://doi.org/10.5601/jelem.2019.24.2.1825

27 Bulut (2022) Mineral composition of emmer wheat (triticum turgidum l. var. dicoccum) landraces. Fresenius Environmental Bulletin 31. https://avesis.kayseri.edu.tr/yayin/ec296ed6-707d-411d-8d95-469be4b97dc0/mineral-composition-of-emmer-wheat-triticum-turgidum-l-var-dicoccum-landraces

28 Cakmak et al. (1997) Differential response of rye, triticale, bread and durum wheats to zinc deficiency in calcareous soils Plant and Soil 188. https://link.springer.com/article/10.1023/A:1004247911381

29 Cakmak et.al.(2010) Biofortification and Localization of Zinc in Wheat Grain. J. Agric. Food Chem. 58, 9092–9102. Doi: 10.1021/jf101197h

30 Chattha et. al. (2017) Biofortification of Wheat Cultivars to Combat Zinc Deficiency. Frontiers in Plant Science. 8:281. Doi: 10.3389/fpls.2017.00281

31 Chaudhari et al. (2022) Fe, Zn & Protein content in grain, per se performance, heterosis, combining ability of grain yield in bread wheat (Triticum aestivum) under normal & late sowing condition. Research Square. https://doi.org/10.21203/rs.3.rs-1338914/v1

32 Ciccolini et al. (2017) Biofortification with Iron and Zinc Improves Nutritional and Nutraceutical Properties of Common Wheat Flour and Bread. Journal of Agriculture and Food Chemistry. https://doi.org/10.1021/acs.jafc.7b01176

33 Colecchia et al. (2013). On the relationship between N management and grain protein content in six durum wheat cultivars in Mediterranean environment. Journal of Plant Interactions 8(3). http://dx.doi.org/10.1080/17429145.2012.710656

34 Curtin et. al.(2008) Wheat (Triticum aestivum) response to micronutrients (Mn, Cu, Zn, B) in Canterbury, New Zealand. New Zealand Journal of Crop and Horticultural Science 36: 169-181. dx.doi.org/10.1080/01140670809510233

35 Dang et al. (2010) Absorption, Accumulation and Distribution of Zinc in Highly-Yielding Winter Wheat. Agricultural Sciences in China 9(7). https://doi.org/10.1016/S1671-2927(09)60178-4

36 Dapkekar et al. (2018) Zinc use efficiency is enhanced in wheat through nanofertilization. Scientific Reports. https://doi.org/10.1038/s41598-018-25247-5

37 Das et al. (2021) Effect of long-term fertilization on zinc distribution and its uptake by wheat (Triticum aestivum). Indian Journal of Agricultural Sciences 91. https://doi.org/10.56093/ijas.v91i3.112526

38 Das et. al. (2020) Zinc biofortification in the Grains of Two Wheat (Triticum aestivum L.) Varieties Through Fertilization. Acta Agrobotanica 73. Doi: 0.5586/aa.7312

39 Demyanyuk et al. (2023) Productivity of Winter wheat under cultivation technologies of different intensity. Zemdirbyste-Agriculture 110(2). https://doi.org/10.13080/z-a.2023.110.013

40 Dhaliwal et. al.(2019) Zinc biofortification of bread wheat, triticale, and durum wheat cultivars by foliar zinc fertilization. Journal of Plant Nutrition 42(8). doi.org/10.1080/01904167.2019.1584189

41 Dhaliwal et.al. (2023) Biofortification of wheat (*Triticum aestivum* L.) genotypes with zinc and manganese lead to improve the grain yield and quality in sandy loam soil. Front. Sustain. Food Syst. 7:1164011. doi: 10.3389/fsufs.2023.1164011

42 EL-Bendary et al. (2013) Zinc efficiency of some Egyptian wheat genotypes grown in Zn-deficient soil. International Journal of AgriScience 37(7). https://www.cabidigitallibrary.org/doi/full/10.5555/20133256608

43 El-Dahshouri et al. (2017) Effect of zinc foliar application at different physiological growth stages on yield and quality of wheat under sandy soil conditions. Agricultural Engineering International: CIGR Journal. https://cigrjournal.org/index.php/Ejounral/article/view/4511/2661

44 El-Marakby et al. (2015) Performance and stability of some bread wheat genotypes for grain yield, protein and gluten contents under different environmental conditions. Arab Univ. J. Agric. Sci. 31.

45 Ercoli et al. (2017) Strong increase of durum wheat iron and zinc content by field-inoculation with arbuscular mycorrhizal fungi at different soil nitrogen availabilities. Plant Soil 419. https://doi.org/10.1007/s11104-017-3319-5

46 Esfandiari and Abdoli (2016) Wheat Biofortification Through Zinc Foliar Application and Its Effects on Wheat Quantitative and Qualitative Yields under Zinc Deficient Stress. Araştırma Makalesi 26(4): 529-537. doi.org/10.29133/yyutbd.282759

47 Farahbakhsh & Sirjani (2019) Enrichment of wheat by zinc fertilizer, mycorrhiza and preharvest drought stress. Turkish Journal of Field Crops 24(1). https://doi.org/10.17557/tjfc.562627

48 Feng et al. (2011) Identification of germplasm with enriched micronutrients of wild emmer and progeny of wild emmer × common wheat. Chinese Journal of Eco-Agriculture. https://doi.org/10.3724/SP.J.1011.2011.01205

49 Ficco et al. (2009) Phytate and mineral elements concentration in a collection of Italian durum wheat cultivars. Field Crops Research 111: 235. doi:10.1016/j.fcr.2008.12.010

50 Forster et al. (2018) Type and placement of zinc fertilizer impacts cadmium content of harvested durum wheat grain. Journal of Plant Nutrition 41(11). https://doi.org/10.1080/01904167.2018.1457687

51 Froese et al. (2020) Response of canola, wheat, and pea to foliar phosphorus fertilization at a phosphorus-deficient site in eastern Saskatchewan. Canadian Journal of Plant Science 100. https://doi.org/10.1139/cjps-2019-0276

52 Gao & Grant (2011) Interactive effect of N fertilization and tillage management on Zn biofortification in durum wheat (Triticum durum). Can. J. Plant Sci. 115. doi:10.4141/CJPS2011-050

53 Gao et al. (2012) Grain concentrations of protein, iron and zinc and bread making quality in spring wheat as affected by seeding date and nitrogen fertilizer management. Journal of Geochemical Exploration 121. https://doi.org/10.1016/j.gexplo.2012.02.005

54 Gao et al. (2010) Mycorrhizal colonization and grain Cd concentration of field-grown durum wheat in response to tillage, preceding crop and phosphorus fertilization. Journal of the Science of Food and Agriculture 90(5). https://doi.org/10.1002/jsfa.3878

55 Gao et al. (2011) Grain cadmium and zinc concentrations in wheat as affected by genotypic variation and potassium chloride fertilization. Field Crops Research 122. https://doi.org/10.1016/j.fcr.2011.03.005

56 Garvin et al. (2006) Historical shifts in the seed mineral micronutrient concentration of US hard red winter wheat germplasm. Journal of the Science of Food and Agriculture 86(13). http://dx.doi.org/10.1002/jsfa.2601

57 Gashu et al. (2021) The nutritional quality of cereals varies geospatially in Ethiopia and Malawi. Nature 594: 71–76. https://doi.org/10.1038/s41586-021-03559-3

58 Gautam et al. (2020) Marker-assisted pyramiding of genes/QTL for grain quality and rust resistance in wheat (Triticum aestivum L.). Molecular Breeding 40(49). https://doi.org/10.1007/s11032-020-01125-9

59 Gawalko et al. (2002) Cadmium, copper, iron, manganese, selenium, and zinc in Canadian spring wheat. Communications in Soil Science and Plant Analysis 33: 15-18. https://doi.org/10.1081/CSS-120014510

60 Ghafari and Razmjoo (2015) Response of Durum Wheat to Foliar Application of Varied Sources and Rates of Iron Fertilizers. Journal of Agriculture and Science technology 17: 321-331

61 Ghasemi et al. (2013) The effectiveness of foliar applications of synthesized zinc-amino acid chelates in comparison with zinc sulfate to increase yield and grain nutritional quality of wheat. European Journal of Agronomy. http://dx.doi.org/10.1016/j.eja.2012.10.012

62 Ghehsareh & Kamran (2010) Effects of different amounts of zinc and iron application on quantitative and qualitative yield of wheat (Triticum durum L.) in northern Hormozgan province in Iran. Research on Crops 11(3)

63 Ghiasi et al. (2016) Agronomic and economic efficiency of ground tire rubber and rubber ash used as zinc fertilizer sources for wheat. Journal of Plant Nutrition 39. DOI: 10.1080/01904167.2015.1109124

64 Ghimire et al. (2021) Effects of cultivars and nitrogen management on wheat grain yield and protein. Agronomy Journal 113. DOI: 10.1002/agj2.20836

65 Ghorttapeh et al. (2018) The study of genetic variability in association with zinc uptake efficiency in Triticum aestivum l genotypes. Proceeding of the 3rd International Conference on Bioscience and Biotechnology volume 3. https://doi.org/10.17501/biotech.2018.3104

66 Gomaa et al. (2015) Effect of Some Macro and Micronutrients Application Methods on Productivity and Quality of Wheat (Triticum aestivum, L.). Middle East Journal of Agriculture Research 4(1). https://curresweb.com/mejar/mejar/2015/1-11.pdf

67 Gomaa et al. (2018) Response of Some Egyptian and Iraqi Wheat Cultivars to Mineral and NanFertilization. Egyptian Academic Journal of Biological Sciences 9(1). https://journals.ekb.eg/article_16726_845255933b6012c2a0c74b415c527740.pdf

68 Gomez-Becerra et al. (2010) Grain concentrations of protein and mineral nutrients in a large collection of spelt wheat grown under different environments. Journal of Cereal Science 52(3). https://doi.org/10.1016/j.jcs.2010.05.003

69 Gomez-Coronado et al. (2018) Potential of advanced breeding lines of bread-making wheat to accumulate grain minerals (Ca, Fe, Mg and Zn) and low phytates under Mediterranean conditions. Journal of Agronomy and Crop Science 205(3). https://doi.org/10.1111/jac.12325

70 Gomez-Coronado et. al. (2017) Combined Zinc and Nitrogen Fertilization in Different Bread Wheat Genotypes Grown under Mediterranean Conditions. Cereal Research Communications 45(1). DOI: 10.1556/0806.44.2016.046

71 Gomez-Coronado et. al.(2016) Zinc (Zn) concentration of bread wheat grown under Mediterranean conditions as affected by genotype and soil/foliar Zn application. Plant and Soil DOI 10.1007/s11104-015-2758-0

72 Gondek (2014) Yield and selected indices of grain quality in spring wheat (triticum aestivum l.) depending on fertilization. Journal of Elementology 4. http://dx.doi.org/10.5601/jelem.2013.18.4.532

73 Graham et al. (1992) Selecting zinc-efficient cereal genotypes for soils of low zinc status. Plant and Soil 146. https://link.springer.com/article/10.1007/BF00012018

74 Grela (1996) Nutrient Composition and Content of antinutritional Factors in Spelt (Triticurn spelta L) Cultivars. Journal of the Science of Food and Agriculture 71(3). Doi: 10.1002/(SICI)1097-0010(199607)71

75 Gruter et al. (2017) Green manure and long-term fertilization effects on soil zinc and cadmium availability and uptake by wheat (Triticum aestivum L.) at different growth stages. Science of the Total Environment. http://dx.doi.org/10.1016/j.scitotenv.2017.05.070

76 Habib, M. (2009) Effect of foliar application of Zn and Fe on wheat yield and quality. African Journal of Biotechnology 8 (24): 6795-6798

77 Hafeez et. al. (2021) Application of Zinc and Iron-Based Fertilizers Improves the Growth Attributes, Productivity, and Grain Quality of Two Wheat (Triticum aestivum) Cultivars. Frontiers in Nutrition 8. doi: 10.3389/fnut.2021.779595

78 Hao et al. (2021) Effects of foliar application of micronutrients on concentration and bioavailability of zinc and iron in wheat landraces and cultivars. Scientific Reports 11: 22782. https://doi.org/10.1038/s41598-021-02088-3

79 Hao et al. (2022) Did wheat breeding simultaneously alter grain concentrations of macro and micro-nutrient over the past 80 years of cultivar releasing in China? Frontiers in Plant Science 13. https://doi.org/10.3389/fpls.2022.872781

80 Hassan et al. (2019) Agronomic Biofortification to Improve Productivity and Grain Zn Concentration of Bread Wheat. International Journal of Agriculture & Biology. http://doi.org/10.17957/IJAB/15.0936

81 Hocaoğlu et al (2020) Changes in the Grain Element Contents of Durum Wheat Varieties of Turkey Registered between 1967–2010. Communications in Soil Science and Plant Analysis 51:4, 431-439. 10.1080/00103624.2019.1709487

82 Huertas-Garcia et al. (2024) Genetic variability for grain components related to nutritional quality in spelt and common wheat. J. Agric. Food Chem. 71: 10598−10607. https://doi.org/10.1021/acs.jafc.3c02366

83 Hui et al. (2019) Critical concentration of available soil phosphorus for grain yield and zinc nutrition of Winter wheat in a zinc-deficient calcareous soil. Plant and Soil ??? https://doi.org/10.1007/s11104-019-04273-w

84 Hussain et al (2011) Physiochemical traits, productivity and net return of wheat as affected by phosphorus and zinc requirements under arid climates. Pakistan Journal of Botany 43(2)

85 Hussain et al (2020) Potassium enhanced grain zinc accumulation in wheat grown on a calcareous saline-sodic soil. Pakistan Journal of Botany 52. http://dx.doi.org/10.30848/PJB2020-1

86 Hussain et al. (2010) Mineral Composition of Organically Grown Wheat Genotypes: Contribution to Daily Minerals Intake. International Journal of Environmental Research and Public Health 7(9). https://doi.org/10.3390/ijerph7093442

87 Hussan et. al. (2021) Impact of soil applied humic acid, zinc and boron supplementation on the growth, yield and zinc translocation in wheat. Asian J Agric & Biol ???. DOI: 10.35495/ajab.2021.02.080

88 Ikhtiar & Alam (2007) Nutritional composition of Pakistani wheat varieties. Science Letters 8. https://link.springer.com/article/10.1631/jzus.2007.B0555

89 Ivanovic et al. (2021) Zinc biofortification of bread Winter wheat grain by single zinc foliar application. Cereal Research Communications ??? https://doi.org/10.1007/s42976-021-00144-2

90 Jablonskytė-raščė et al. (2013) Evaluation of productivity and quality of common wheat (*Triticum aestivum* L.) and spelt (*Triticum spelta* L.) in relation to nutrition conditions. Zemdirbyste-Agriculture 100: 45–91.

91 Jalal et al. (2020) Agro-Biofortification of Zinc and Iron in Wheat Grains. Gesunde Pflanzen 72. https://doi.org/10.1007/s10343-020-00505-7

92 Jalal et al. (2023) Integrated use of plant growth promoting bacteria and nanozinc foliar spray is a sustainable approach for wheat biofortification, yield, and zinc use efficiency. Frontiers in Plant Science 14. https://doi.org/10.3389/fpls.2023.1146808

93 Janczak-Pieniazek et al. (2020) Effect of high nitrogen doses on yield, quality and chemical composition grain of winter wheat cultivars. Journal of Elementology ?? DOI: 10.5601/jelem.2020.25.1.1994

94 Janczak-Pieniazek et al. (2022) The course of physiological processes, yielding, and grain quality of hybrid and population wheat as affected by integrated and conventional cropping systems. Agronomy 12(6). https://doi.org/10.3390/agronomy12061345

95 Jat et. al. (2014) Effect of potassium and zinc fertilizer on crop yield, nutrient uptake and distribution of potassium and zinc fractions in Typic Ustipsamment. Indian Journal of Agricultural Sciences 84: 832–8,

96 Johansson et al. (2021) Locally Adapted and Organically Grown Landrace and Ancient Spring Cereals—A Unique Source of Minerals in the Human Diet. Foods 10(2). https://doi.org/10.3390/foods10020393

97 Joshi et. al. (2010) Genotype environment interaction for zinc and iron concentration of wheat grain in eastern Gangetic plains of India. Field Crops Research 116: 268–277. 10.1016/j.fcr.2010.01.004

98 Kandil et al. (2017) Response of some wheat cultivars to nano- , mineral fertilizers and amino acids foliar application. Alexandria Science Exchange Journal 38(1). http://dx.doi.org/10.21608/asejaiqjsae.2017.1877

99 Kandoliya et. al. (2018) Effect of zinc and iron application on leaf chlorophyll, carotenoid, grain yield and quality of wheat in calcareous soil of Saurashtra region. International Journal of Chemical Studies 6(4): 2092-2095. https://www.researchgate.net/publication/334669971

100 Kariithi et al. (2016) Evaluation of grain nutritional quality and resistant starch content in Kenyan bread wheat varieties. African Crop Science Journal 24(4). http://dx.doi.org/10.4314/acsj.v24i4.8

101 Karim et al. (2012) Alleviation of drought stress in Winter wheat by late foliar application of zinc, boron, and manganese. Journal of Plant Nutrition and Soil Science 175(1). https://doi.org/10.1002/jpln.201100141

102 Khalili et. al. (2023) Role of temporal zn fertilization along with zn solubilizing bacteria in enhancing zinc content, uptake, and zinc use efficiency in wheat genotypes and its implications for agronomic biofortification. Agronomy 13: 2677. https://doi.org/10.3390/agronomy13112677

103 Khan et al. (2018) Zinc bioavailability and nitrogen concentration in grains of wheat crop sprayed with zinc sulfate, ammonium sulfate, ammonium chloride, and urea. Journal of Plant Nutrition 41(15). https://doi.org/10.1080/01904167.2018.1484472

104 Khan et al. (2023) Loading of zinc and iron in grains of different wheat genotypes in the calcareous and floodplain soils of Bangladesh. Heliyon ?? https://doi.org/10.1016/j.heliyon.2023.e19039

105 Khattak et. al. (2017) Improving Farmer's Income and Nitrogen Use Efficiency of Dry Land Wheat through Soil and Foliar Application of N-Fertilizer. Sarhad Journal of Agriculture 33: 348. Doi: 10.17582/journal.sja/2017/33.3.344.349

106 Khokhar et. al. (2018) Variation in grain Zn concentration, and the grain ionome, in field-grown Indian wheat. PLoS ONE 13(1). doi:10.1371/journal.pone.0192026

107 Khoshgoftarmanesh et al. (2005) Zinc Efficiency of Wheat Cultivars Grown on a Saline Calcareous Soil. Journal of Plant Nutrition 27(11). http://dx.doi.org/10.1081/PLN-200030068

108 Khoshgoftarmanesh et al. (2012) Classification of wheat genotypes by yield and densities of grain zinc and iron using cluster analysis. Journal of Geochemical Exploration 121. http://dx.doi.org/10.1016/j.gexplo.2012.06.002

109 Kiran et. al. (2021) Concentration and Localization of Fe and Zn in Wheat Grain as Affected by Its Application to Soil and Foliage. Bulletin of Environmental Contamination and Toxicology 106. https://doi.org/10.1007/s00128-021-03183-x

110 Kong et al. (2024) Biofortification: interaction between zinc and selenium regarding their accumulation in wheat. Agronomy 14, 1513. https://doi.org/10.3390/agronomy14071513

111 Krishnappa et al (2022) Genetic dissection of grain iron and zinc, and thousand kernel weight in wheat (*Triticum aestivum* L.) using genome-wide association study. Scientific Reports 12:12444. https://doi.org/10.1038/s41598-022-15992-z

112 Kumar et al. (2018) Agronomic biofortification of zinc in wheat (Triticum aestivum L.). Current Science 115(5). http://dx.doi.org/10.18520/cs/v115/i5/944-948

113 Li et al. (2014) Cereal Research Communications 42. DOI: 10.1556/CRC.2013.0042

114 Liu et. al. (2017) Effects of zinc application rate and zinc distribution relative to root distribution on grain yield and grain Zn concentration in wheat. Plant Soil 411: 167. DOI 10.1007/s11104-016-2953-7

115 Liu et. al. (2019) Zinc uptake, translocation, and remobilization in winter wheat as affected by soil application of zn fertilizer. Frontiers in Plant Science 10: 426. doi: 10.3389/fpls.2019.00426

116 Loes et al. (2020) What should organic farmers grow: heritage or modern spring wheat cultivars? Organic Agriculture 10(1). http://dx.doi.org/10.1007/s13165-020-00301-7

117 Loncaric et al. (2021) Foliar Zinc-Selenium and Nitrogen Fertilization Affects Content of Zn, Fe, Se, P, and Cd in Wheat Grain. Plants 10: 1549. https://doi.org/10.3390/plants10081549

118 Lovegrove et al. (2023) Comparative Compositions of Grain of Bread Wheat, Emmer and Spelt Grown with Different Levels of Nitrogen Fertilisation. Foods 12(4). https://doi.org/10.3390/foods12040843

119 Lu et al. (2024) Cadmium (Cd) Minimization and Zinc (Zn) Biofortification in Wheat (Triticum aestivum L.) Grains by Spraying with the Foliar Zn Fertilizer in Cd-Contaminated Fields. Agronomy 14. https://doi.org/10.3390/agronomy14010018

120 Luis et al. (2021) Zinc Enrichment in Two Contrasting Genotypes of Triticum aestivum L. Grains: Interactions between Edaphic Conditions and Foliar Fertilizers. Plants 10. https://doi.org/10.3390/plants10020204

121 Lungu et al. (2011) Effects of Soil and Foliar Applications of Zinc on Grain Zinc Concentrations of Maize, Sorghum and Wheat in Zambia. International Sorghum and Millet Collaborative Research Support Program. https://digitalcommons.unl.edu/intsormilpubs/43/

122 Ma et al. (2017) Physiological Responses and Yield of Wheat Plants in Zinc-Mediated Alleviation of Drought Stress. Frontiers in Plant Science 8: 863. https://doi.org/10.3389/fpls.2017.00860

123 Ma et al. (2018) Evaluation of Yield, Processing Quality, and Nutritional Quality in Different-Colored Wheat Grains under Nitrogen and Phosphorus Fertilizer Application. Crop Science 58. https://doi.org/10.2135/cropsci2017.03.0152

124 Maamoun & El-Shazly (2014) Effect of Magnetic Iron and Biofertilization Treatments on Wheat (Triticum aestivum L.) Productivity under Salinity Conditions. Egyptian Journal of Agronomy 36(1). https://doi.org/10.21608/AGRO.2014.77

125 Magallanes-Lopez et al. (2017) Variability in iron, zinc and phytic acid content in a worldwide collection of commercial durum wheat cultivars and the effect of reduced irrigation on these traits. Food Chemistry 237. https://doi.org/10.1016/j.foodchem.2017.05.110

126 Manzeke-Kangara et al (2023) Micronutrient Fertilizer Effect on Agro-Fortified Wheat and Teff Grain Nutrient Concentration in Western Amhara. Agronomy 13. https://doi.org/10.3390/agronomy13102598

127 Mao et. al. (2014) Using agronomic biofortification to boost zinc, selenium, and iodine concentrations of food crops grown on the loess plateau in China. Journal of Soil Science and Plant Nutrition 14 (2), 459-470. http://dx.doi.org/10.4067/S0718-95162014005000036

128 McDonald et al. (2008) A simple method to evaluate genetic variation in grain zinc concentration by correcting for differences in grain yield. Plant & Soil 306. https://doi.org/10.1007/s11104-008-9555-y

129 Meena et. al. (2021a) Impact of zinc fortification in bread wheat (Triticum aestivum L.) through soil and foliar application methods. The Pharma Innovation Journal 10(9).

130 Meena et. al. (2021b) Impact of foliar application of different nano-fertilizers on soil microbial properties and yield of Wheat. Journal of Environmental Biology 42. http://doi.org/10.22438/jeb/42/2/MRN-1465

131 Melash et al. (2019) The influence of seeding rate and micronutrients foliar application on grain yield and quality traits and micronutrients of durum wheat. Journal of Cereal Science 85. https://doi.org/10.1016/j.jcs.2018.08.005

132 Melash et al. (2023) Sustainable management practices for durum wheat production: Analyzing specific agronomic interventions on productivity, grain micronutrient content, and quality. Heliyon 9. https://doi.org/10.1016/j.heliyon.2023.e18733

133 Milvojevic et al (2018) Cultivar-specific accumulation of iron, manganese, zinc and copper in Winter wheat grain (Triticum aestivum L.). Journal of Central European Agriculture 19. https://doi.org/10.5513/JCEA01/19.2.2051

134 Montoya et. al. (2020) Zinc–nitrogen interaction effect on wheat biofortification and nutrient use. efficiency. J. Plant Nutr. Soil Sci. 000: 1-11. Doi: 10.1002/jpln.201900339

135 Morgounov et al. (2013) Historical changes in grain yield and quality of spring wheat varieties cultivated in Siberia from 1900 to 2038. Canadian Journal of Plant Science 93. https://doi.org/10.4141/cjps2012-091

136 Mosavian et al. (2021) Do nitrogen and zinc application alleviate the adverse effect of heat stress on wheat (Triticum aestivum L.)? Notulae Botanicae Horti Agrobotanici Cluj-Napoca ??? https://doi.org/10.15835/nbha49212252

137 Murphy et al. (2009) Nutritional and quality characteristics expressed in 31 perennial wheat breeding lines. Renewable Agriculture and Food Systems 24. https://doi.org/10.1017/S1742170509990159

138 Nadeem et. al. (2023) Influence of Zn nutrition on the productivity, grain quality and grain biofortification of wheat under conventional and conservation rice-wheat cropping systems. Archives of Agronomy and Soil Science 66(8). Doi: 10.1080/03650340.2019.1652273

139 Neelam et al. (2012) Evaluation and Identification of Wheat-Aegilops Addition Lines Controlling High Grain Iron and Zinc Concentration and Mugineic Acid Production. Cereal Research Communications 40. http://dx.doi.org/10.1556/CRC.40.2012.1.7

140 Ning et al. (2022) Combined foliar application of zinc sulphate and selenite affects the magnitude of selenium biofortification in wheat (Triticum aestivum L.). Food and Energy Security 11. https://doi.org/10.1002/fes3.342

141 Ning et. al. (2019) Enhancing Zinc Accumulation and Bioavailability in Wheat Grains by Integrated Zinc and Pesticide Application. Agronomy 9: 530. doi:10.3390/agronomy9090530

142 Nitika et al (2008) Physico-chemical characteristics, nutrient composition and consumer acceptability of wheat varieties grown under organic and inorganic farming conditions. International Journal of Food Sciences and Nutrition 59 (3). DOI: 10.1080/09637480701523249

143 Niyigaba et al. (2019) Winter Wheat Grain Quality, Zinc and Iron Concentration Affected by a Combined Foliar Spray of Zinc and Iron Fertilizers. Agronomy 9(5). https://doi.org/10.3390/agronomy9050250

144 Noman et al (2016) Sulphur and Zn management in groundnut *(Arachis hypogaea)*–wheat (*Triticum aestivum*) cropping system: Direct effects on system productivity and residual effects on yield, energetics and Zn biofortification in wheat. Indian Journal of Agricultural Sciences 86 (4). DOI: 10.56093/ijas.v86i4.57434

145 Oury et al. (2006) Genetic variability and stability of grain magnesium, zinc and iron concentrations in bread wheat. European Journal of Agronomy 25(2). https://doi.org/10.1016/j.eja.2006.04.011

146 Ozturk et al. (2009) Localization of iron, zinc, and protein in seeds of spelt (Triticum aestivum ssp. spelta) genotypes with low and high protein concentration. Proceedings of the International Plant Nutrition Colloquium.

147 Pahlavan-Rad & Pessarakli (2009) Response of Wheat Plants to Zinc, Iron, and Manganese Applications and Uptake and Concentration of Zinc, Iron, and Manganese in Wheat Grains. Communications in Soil Science and Plant Analysis 40: 7- 8. https://doi.org/10.1371/journal.pone.0181276

148 Pandey & Kumar 2017 (2017) Effect of sulphur, manganese and zinc on yield, quality and uptake of nutrients by wheat (Triticum aestivum). Annals of Plant and Soil Research 19(4): 403-407

149 Pandey & Rana (2016) Response of wheat to sulphur and zinc nutrition in alluvial soil. Annals of Plant and Soil Research 18(4): 418-422

150 Pant et. al. (2020) Evaluation of biofortified spring wheat genotypes for yield and micronutrient contents. Fundamental and Applied Agriculture 5(1): 78–87. doi: 10.5455/faa.79404

151 Paramesh et al. (2020) Role of Integrated Nutrient Management and Agronomic Fortification of Zinc on Yield, Nutrient Uptake and Quality of Wheat. Sustainability 12(9). https://doi.org/10.3390/su12093513

152 Peck et al. (2008) Zinc nutrition influences the protein composition of flour in bread wheat (Triticum aestivum L.). Journal of Cereal Science 47(2). https://doi.org/10.1016/j.jcs.2007.04.006

153 Peleg et al (2008) Grain zinc, iron and protein concentrations and zinc-efficiency in wild emmer wheat under contrasting irrigation regimes. Plant and Soil 306: 57. DOI 10.1007/s11104-007-9417-z

154 Perilli et al. (2010) Cadmium concentration in durum wheat grain (Triticum turgidum) as influenced by nitrogen rate, seeding date and soil type. Journal of the Science of Food and Agriculture 90(5): https://doi.org/10.1002/jsfa.3889

155 Petrovi´c et al. (2020) Variation in Nutritional Value of Diverse Wheat Genotypes. Agronomy 14, 311. https://doi.org/10.3390/agronomy14020311

156 Porter & Paulsen (1983) Grain Protein Response to Phosphorus Nutrition of Wheat. Agronomy Journal 75. https://doi.org/10.2134/agronj1983.00021962007500020032x

157 Qamari et al. (2023) Response of wheat cultivars to zinc application for seed yield and quality improvement. The Journal of Agricultural Science. https://doi.org/10.1017/S0021859623000473

158 Rachoń and Szumi (2009) Comparison of chemical composition of selected winter wheat species. Journal of Elementology 14: 135-146

159 Rachoń et al. (2015) Nutritional value and mineral composition of grain of selected wheat species depending on the intensity of a production technology. Journal of Elementology 20(3): 705-715. DOI: 10.5601/jelem.2014.19.4.640

160 Ram et. al. (2013) Potentialities in Using Foliar Zinc Sulphate fertilizer with pesticides for enriching Zinc in wheat grains in India. International Plant Nutrition Colloquium ???

161 Ram et. al. (2016) Biofortification of wheat, rice and common bean by applying foliar zinc fertilizer along with pesticides in seven countries. Plant Soil 403(1-2). 10.1007/s11104-016-2815-3

162 Ramzan et. al. (2020) Biofortification with Zinc and Iron Improves the Grain Quality and Yield of Wheat Crop. International Journal of Plant Production 14: 501–510. https://doi.org/10.1007/s42106-020-00100-w

163 Ranchon et al. (2012) Comparison of the chemical composition of spring durum wheat grain (triticum durum)and common wheat grain (triticum aestivum ssp. vulgare). Journal of Elementology 17. https://doi.org/10.5601/jelem.2012.17.1.10

164 Rathan et al (2021) Identification of Genetic Loci and Candidate Genes Related to Grain Zinc and Iron Concentration Using a Zinc-Enriched Wheat 'Zinc-Shakti. Front. Genet. 12:652653. https://doi.org/10.3389/fgene.2021.652653

165 Rashid et al. (2019) Effect of zinc-biofortified seeds on grain yield of wheat, rice, and common bean grown in six countries. Journal of Plant Nutrition and Soil Science 182. https://doi.org/10.1002/jpln.201800578

166 Rathi and Jood (2016) Variability in nutrient and antinutrient composition of Durum wheat varieties. Annals of Agri-bio Research 21: 202-205

167 Rawat et al. (2009) Evaluation and utilization of Aegilops and wild Triticum species for enhancing iron and zinc content in wheat. Genet Resour Crop Evol 56:53. DOI 10.1007/s10722-008-9344-8

168 Rehman et al. (2018) Characterizing Bread Wheat Genotypes of Pakistani Origin for Grain Zinc Biofortification Potential. Journal of the Science of Food and Agriculture ?? https://doi.org/10.1002/jsfa.9010

169 Reynolds-Marzal et al (2021) Combined selenium and zinc biofortification of bread-making wheat under mediterranean conditions. Plants 10. https://doi.org/10.3390/plants10061209

170 Reznick et al. (2021) Nitrogen and cultivars as field strategies to improve the nutritional status of wheat grain and flour. Journal of Cereal Science 102. https://doi.org/10.1016/j.jcs.2021.103290

171 Rodehutscord et al. (2016) Variation in chemical composition and physical characteristics of cereal grains from different genotypes. Archives of Animal Nutrition 70. https://doi.org/10.1080/1745039X.2015.1133111

172 Ryan et al. (2004) Grain mineral concentrations and yield of wheat grown under organic and conventional management. Journal of the Science of Food and Agriculture 84. http://dx.doi.org/10.1002/jsfa.1634

173 Sˇkrbic et al. (2005) Principal Component Analysis of Trace Elements in Serbian Wheat. Journal of Agricultural and Food Chemistry 53. https://doi.org/10.1021/jf0402577

174 Sadhegi et al. (2021) Effect of zinc and magnesium fertilizers on the yield and some characteristics of wheat (Triticum aestivum l.) seeds in two years. International Journal of Agronomy 2021. ??? https://doi.org/10.1155/2021/8857222

175 Saha et al. (2017) Rescheduling zinc fertilization and cultivar choice improve zinc sequestration and its bioavailability in wheat grains and flour. Field Crops Research 200. http://dx.doi.org/10.1016/j.fcr.2016.09.006

176 Saleem et.al. (2015) Improvement of Wheat Grain Zinc and Zinc Daily Intake by Biofortification with Zinc. International Journal of Plant & Soil Science 8(5): 1-6. Doi: 10.9734/IJPSS/2015/19739

177 Schweizer et al. (2018) Impact of organic and conventional farming systems on wheat grain uptake and soil bioavailability of zinc and cadmium. Science of the Total Environment ??? https://doi.org/10.1016/j.scitotenv.2018.05.187

178 Shah et al. (2023) Enhancements in yield, agronomic, and zinc recovery efficiencies of rice-wheat system through bioactive zinc coated urea application in Aridisols. Plos One 18. https://doi.org/10.1371/journal.pone.0282615

179 Shahane et al. (2017) Quantifying the contribution of microbial inoculation and zinc fertilization to growth, yield and economics of wheat (*Triticum aestivum*) in different methods of cultivation. Indian Journal of Agricultural Sciences 87(8).

180 Shariatipour et al. (2020) Foliar applied zinc increases yield, zinc concentration, and germination in wheat genotypes. Agronomy Journal 112. https://doi.org/10.1002/agj2.20117

181 Sher et al. (2020) Grain zinc and iron enrichment through foliar application augments wheat yield under varying nitrogen regimes. Pakistan Journal of Botany 52. http://dx.doi.org/10.30848/PJB2020-1(25)

182 Sher et. al. (2022) Exogenous Application of Zinc Sulphate at Heading Stage of Wheat Improves the Yield and Grain Zinc Biofortification. Agronomy 12(3): 734. doi.org/10.3390/agronomy12030734

183 Shi et al. (2008) Identification of quantitative trait locus of zinc and phosphorus density in wheat (Triticum aestivum L.) grain. Plant & Soil 306. https://doi.org/10.1007/s11104-007-9483-2

184 Shivay et al. (2008) Relative efficiency of zinc oxide and zinc sulphate-enriched urea for spring wheat. Nutrient Cycling in Agroecosystems ?? https://doi.org/10.1007/s10705-008-9186-y

185 Shoormij et al. (2022) Combined foliar application of Zn and Fe increases grain micronutrient concentrations and alleviates water stress across diverse wheat species and ploidal levels. Scientific Reports ??? https://doi.org/10.1038/s41598-022-24868-1

186 Silva et al. (2020) Water stress alters physical and chemical quality in grains of common bean, triticale and wheat. Agricultural Water Management 231. https://doi.org/10.1016/j.agwat.2020.106023

187 Singh & Sandhu (2021) Impact of zinc fertilization on growth, yield and quality of zero till wheat (Triticum aestivum L.). Journal of Environmental Biology 42. http://doi.org/10.22438/jeb/42/2/MRN-1598

188 Singh & Singh (2017) Productivity, quality and nutrients uptake of some rabi crops under zinc nutrition in alluvial soil. Annals of Plant and Soil Research 19(4): 355-359

189 Singh et al. (2021) Engineered zinc oxide nanoparticles: an alternative to conventional zinc sulphate in neutral and alkaline soils for sustainable wheat production. Biotech 11: 322. https://doi.org/10.1007/s13205-021-02861-1

190 Singh et al. (2020) Categorization of diverse wheat genotypes for zinc efficiency based on higher yield and uptake efficiency. Journal of Soil Science and Plant Nutrition ??? https://doi.org/10.1007/s42729-019-00153-5

191 Singh et. al.(2022) Determining the Effect of Zinc Fortification on Growth, Yield and Economics of Wheat (Triticum aestivum L.) Under Irrigated Condition. Asian Journal of Agricultural Extension, Economics & Sociology 40: 10. DOI: 10.9734/AJAEES/2022/v40i1031049

192 Soleimani et al (2012) Cumulative and residual effects of zinc sulfate on grain yield, zinc, iron, and copper concentration in corn and wheat. Journal of Plant Nutrition 35:85. DOI: 10.1080/01904167.2012.631669

193 Souza et al. (2014) Genotypic variation of zinc and selenium concentration in grains of Brazilian wheat lines. Plant Science 224. http://dx.doi.org/10.1016/j.plantsci.2014.03.022

194 Souza et. al.(2019) Zinc and amino acids on wheat-soybean intercropping under no-till management. Journal of Plant Nutrition 42. DOI: 10.1080/01904167.2019.1648665

195 Srinivasa et al. (2014) Accessing spelt gene pool to develop well adapted zinc- and iron-rich bread wheat. Crop Science 54. https://doi.org/10.2135/cropsci2013.12.0801

196 Stefanović et al. (2008) Undesirable metals content in wheat of different wheat varieties. Acta Periodica Technologica 39. https://doi.org/10.2298/APT0839069S

197 Stepien & Wojtkowiak (2016) Effect of foliar application of Cu, Zn, and Mn on yield and quality indicators of Winter wheat grain. Chilean Journal of Agricultural Research 76. https://doi.org/10.4067/S0718-58392016000200012

198 Stępień et al. (2016) Nutritional and technological characteristics of common and spelt wheats are affected by mineral fertilizer and organic stimulator Nano-Gro**®**. Acta Sci. Pol. Agricultura 15: 49-64.

199 Suchowilska et al. (2020) Do Triticum aestivum L. and Triticum spelta L. hybrids constitute a promising source material for quality breeding of new wheat varieties? Agronomy 10: 43

200 Sulek et al. (2023) Effect of Production Technology Intensity on the Grain Yield, Protein Content and Amino Acid Profile in Common and Durum Wheat Grain. Plants 12: https://doi.org/10.3390/plants12020364

201 Tabekhia & Donnelly (1982) Phytic acid in durum wheat and its milled products. Cereal Chem 64: ???

202 Tadesse et al. (2023) Single- and multi-trait genomic prediction and genome-wide association analysis of grain yield and micronutrient-related traits in ICARDA wheat under drought environment. Molecular Genetics and Genomics 298. https://doi.org/10.1007/s00438-023-02074-6

203 Tahir et al. (2021) Grain micronutrient evaluation of wheat (Triticum aestivum) germplasm and molecular characterisation via genic and random SSR markers. Crop & Pasture Science 73. https://doi.org/10.1071/CP21116

204 Taskin and Gunes(2022) Iron biofortifcation of wheat grains by foliar application of nano zero-valent iron (nZVI) and other iron sources with urea. Journal of Soil Science and Plant Nutrition 22(9): 1-11. 10.1007/s42729-022-00946-1

205 Thapa et. al. (2022) Variation in grain zinc and iron concentrations, grain yield and associated traits of biofortified bread wheat genotypes in Nepal. Frontiers in Plant Science 13: 881965. doi: 10.3389/fpls.2022.881965

206 Vaziri et al. (2023) Enhancing grain yield and zinc content in bread wheat using zinc and nitrogen application under supplementary irrigation treatments. Romanian Agricultural Research 40: ????

207 Vazquez et al. (2018) Grain mineral density of bread and durum wheat landraces from geochemically diverse native soils. Crop & Pasture Science 69. https://doi.org/10.1071/CP17306

208 Velu et al. (2017a) QTL mapping for grain zinc and iron concentrations and zinc efficiency in a tetraploid and hexaploid wheat mapping populations. Plant & Soil 411. https://doi.org/10.1007/s11104-016-3025-8

209 Velu et al. (2017b) Characterization of grain protein content gene (GPC-B1) introgression lines and its potential use in breeding for enhanced grain zinc and iron concentration in spring wheat. Acta Physiologiae Plantarum 39. https://doi.org/10.1007/s11738-017-2509-3

210 Velu et al. (2017c) Genetic impact of Rht dwarfing genes on grain micronutrients concentration in wheat. Field Crops Research 214. http://dx.doi.org/10.1016/j.fcr.2017.09.030

211 Velu et al. (2019) Assessing genetic diversity to breed competitive biofortified wheat with enhanced grain zn and fe concentrations. Frontiers in Plant Science 9. 10.3389/fpls.2018.01971

212 Wan et al. (2022) Localisation of iron and zinc in grain of biofortified wheat Journal of Cereal Science 105: 103470. https://doi.org/10.1016/j.jcs.2022.103470

213 Wang et al. (2017a) Genotypic variability in growth and yield of spring wheat varieties in long-term soil fertility regimes. XVIII International Plant Nutrition Colloquium 19-24 August 2017. Copenhagen, Denmark. https://www.cabidigitallibrary.org/doi/pdf/10.5555/20209903475

214 Wang et al. (2015) An effective strategy to improve grain zinc concentration of winter wheat, Aphids prevention and farmers' income. Field Crops Research 184. https://doi.org/10.1016/j.fcr.2015.08.015

215 Wang et al. (2015) Effects of increasing rates of zinc fertilization on phytic acid and phytic acid/zinc molar ratio in zinc bio-fortified wheat. Field Crops Research ???? http://dx.doi.org/10.1016/j.fcr.2015.09.007

216 Wang et al. (2017b) Effects of Zn, macronutrients, and their interactions through foliar applications on winter wheat grain nutritional quality. Plos One 12. https://doi.org/10.1371/journal.pone.0181276

217 Wang et al. (2021) Source–Sink Manipulation Affects Accumulation of Zinc and Other Nutrient Elements in Wheat Grains. Plants 10. https://doi.org/10.3390/plants10051032

218 Wang et. al. (2012) Different increases in maize and wheat grain zinc concentrations caused by soil and foliar applications of zinc in Loess Plateau, China. Field Crops Research 135 (2012) 89–96. https://doi.org/10.1016/j.fcr.2012.07.010

219 White et al. (1981) Variation in Nitrogen, Sulfur, Selenium, Cobalt, Manganese, Copper and Zinc Contents of Grain from Wheat and Two Lupin Species Grown in a Range of Mediterranean Environments. Australian Journal of Agricultural Research 32. https://doi.org/10.1071/AR9810047

220 Wojtkowiak and Stępień (2015) Nutritive value of spelt (*Triticum aestivum* spp. *spelta* L.) as influenced by the foliar application of copper, zinc and manganese. Zemdirbyste-Agriculture 102; 389–396. DOI 10.13080/z-a.2015.102.049

221 Xia et al. (2018) Rational Application of Fertilizer Nitrogen to Soil in Combination With Foliar Zn Spraying Improved Zn Nutritional Quality of Wheat Grains. Frontiers in Plant Science 9. https://doi.org/10.3389/fpls.2018.00677

222 Xia et al. (2020) Methods of selenium application differentially modulate plant growth, selenium accumulation and speciation, protein, anthocyanins and concentrations of mineral elements in purple-grained wheat. Frontiers in Plant Science 11. doi: 10.3389/fpls.2020.01114

223 Xu et al. (2022) Spraying high concentrations of chelated zinc enhances zinc biofortification in wheat grain. Journal of the Science of Food and Agriculture 102: 20. https://doi.org/10.1002/jsfa.11705

224 Yadav et. al. (2020) Enhancing nutrient translocation, yields and water productivity of wheat under rice–wheat cropping system through zinc nutrition and residual effect of green manuring. Journal of Plant Nutrition 43. DOI: 10.1080/01904167.2020.1798997

225 Yang et al. (2023) High-Zn wheat alleviates P-Zn antagonism by improving Zn activation,acquisition, and translocation at key growth stages. Field Crops Research 304. https://doi.org/10.1016/j.fcr.2023.109149

226 Yilmaz et. al. (1997) Effect of different zinc application methods on grain yield and zinc concentration in wheat cultivars grown on zinc-deficient calcareous soils. Journal of Plant Nutrition 20:4-5. https://doi.org/10.1080/01904169709365267

227 Yu et al. (2021) Foliar Zinc Application to Wheat May Lessen the Zinc Deficiency Burden in Rural Quzhou, China. Frontiers in Nutrition ???? https://doi.org/10.3389/fnut.2021.697817

228 Zarea & Karimi (2023) Grain yield and quality of wheat are improved through postflowering foliar application of zinc and 6- benzylaminopurine under water deficit condition. Frontiers in Plant Science ??? https://doi.org/10.3389/fpls.2022.1068649

229 Zhang et al. (2010a) Iron and zinc concentrations in grain and flour of winter wheat as affected by foliar application. Journal of Agricultural and Food Chemistry 58. https://doi.org/10.1021/jf103039k

230 Zhang et al. (2010b) Mineral element concentrations in grains of Chinese wheat cultivars. Euphytica 174. https://doi.org/10.1007/s10681-009-0082-6

231 Zhang et al. (2012) The reduction in zinc concentration of wheat grain upon increased phosphorus-fertilization and its mitigation by foliar zinc application. Plant and Soil ??? https://doi.org/10.1007/s11104-012-1238-z

232 Zhang et al. (2017) Overuse of Phosphorus Fertilizer Reduces the Grain and Flour Protein and Zinc Bioavailability of Winter Wheat (Triticum aestivum L.). Journal of Agricultural and Food Chemistry ??? http://doi.org/10.1021/acs.jafc.6b04778

233 Zhang et. al. (2011) Zinc biofortification of wheat through fertilizer applications in different locations of China. Field Crops Research 125. 10.1016/j.fcr.2011.08.003

234 Zhao et al. (2009) Variation in mineral micronutrient concentrations in grain of wheat lines of diverse origin. Journal of Cereal Science 49. https://doi.org/10.1016/j.jcs.2008.11.007

235 Zhao et al. (2014) Comparison of soil and foliar zinc application for enhancing grain zinc content of wheat when grown on potentially zinc-deficient calcareous soils. Journal of the Science of Food and Agriculture 94. https://doi.org/10.1002/jsfa.6518

236 Zhao et al. (2016) Effect of nitrogen fertilizers on zinc absorption and translocation in Winter wheat. Journal of Plant Nutrition 39. https://doi.org/10.1080/01904167.2015.1106560

237 Zhao et al. (2018) Effects of ZnSO4 and Zn-EDTA broadcast or banded to soil on Zn bioavailability in wheat (Triticum aestivum L.) and Zn fractions in soil. Chemosphere 205. https://doi.org/10.1016/j.chemosphere.2018.04.115

238 Zhao et. al. (2020) Combined soil and foliar ZnSO4 application improves wheat grain Zn concentration and Zn fractions in a calcareous soil. Eur J Soil Sci. 71. https://doi.org/10.1111/ejss.12903

239 Zia et. al. (2020) Site-Specific Factors Influence the Field Performance of a Zn-Biofortified Wheat Variety. Frontiers in Sustainable Food Systems 4: 135. https://doi.org/10.3389/fsufs.2020.00135

240 Zou et al. (2012) Biofortification of wheat with zinc through zinc fertilization in seven countries. Plant and Soil ?? https://doi.org/10.1007/s11104-012-1369-2

241 Zou et. al. (2019) Simultaneous biofortification of wheat with zinc, iodine, selenium, and iron through foliar treatment of a micronutrient cocktail in six countries. Journal of Agricultural and Food Chemistry 67: 8096. https://doi.org/10.1021/acs.jafc.9b01829

242 Żuk-Gołaszewska et al. (2022) Nutritional properties of organic spelt wheats in different growth stages and the resulting flours. Journal of Elementology 27(3): 645-662. DOI: 10.5601/jelem.2022.27.1.2267

243 Zulfiqar et. al. (2020) Iron Nutrition Improves Productivity, Profitability, and Biofortification of Bread Wheat under Conventional and Conservation Tillage Systems. Journal of Soil Science and Plant Nutrition 20: 1298–1310. https://doi.org/10.1007/s42729-020-00213-2

Table S2. Summary table of grain Zn, Fe, protein and phytate concentrations

Table S2. The number of studies and total number of observations (n) used for estimating grain zinc (Zn), iron (Fe), protein and phytate concentrations and the probability (ϕ in %) of exceeding the target concentrations of Zn (38 mg kg^{-1}), Fe (59 mg/kg) and protein (12%) in different species of cultivated wheat.

A. Analysis by wheat species

	Variable	Studies (n) †	Median (95% CI)‡	Q1	Q3	CV (%)	ϕ > target
Zinc (mg kg^{-1})	Bread	197 (7753)	34.4 (34.2, 34.8)	27.0	43.4	40.0	38.9
	Durum	33 (997)	35.7 (35.1, 36.6)	30.5	53.1	35.3	42.7
	Spelt	17 (443)	41.8 (41.0, 42.7)	37.6	46.5	18.3	80.3
	Emmer	5 (61)	92.0 (88.0, 101.0)	53.9	108.5	51.5	72.0
Iron (mg kg^{-1})	Bread	103 (5678)	37.9 (37.7, 38.2)	32.7	43.1	37.6	7.7
	Durum	26 (863)	36.9 (36.0, 37.4)	33.0	43.0	32.1	6.6
	Spelt	17 (443)	45.3 (44.6, 45.9)	41.4	50.0	19.2	59.0
	Emmer	5 (61)	62.0 (60.0, 68.0)	47.8	72.0	44.7	4.7
Protein (%)	Bread	95 (8782)	12.5 (12.5, 12.6)	11.4	13.6	14.6	63.8
	Durum	24 (672)	13.3 (13.2, 13.5)	12.3	14.9	21.1	58.6
	Spelt	12 (91)	15.7 (15.2, 16.6)	13.4	19.1	21.8	97.8
	Emmer	1 (47)	23.1 (21.4, 24.7)	20.4	25.8	19.2	94.5
Phytate (%)	Bread	33 (972)	0.86 (0.85, 0.88)	0.74	1.05	51.1	NA
	Durum	7 (150)	0.75 (0.73, 0.83)	0.63	1.09	47.7	NA
	Spelt	1 (359)	1.13 (1.12, 1.15)	1.05	1.20	10.1	NA
	Emmer	0	NA	NA	NA	NA	NA

B. Analysis by Zn fertilization treatment of bread wheat

	Zn fertilizer	Studies (n) †	Median (95% CI)‡	Q1	Q3	CV (%)	ϕ > target
Zinc (mg kg^{-1})	Without Zn	185 (5899)	33.2 (32.9, 33.6)	26.0	41.2	38.3	33.5
	With Zn	117 (2024)	40.0 (39.5, 41.0)	31.5	50.7	38.6	56.4
Iron (mg kg^{-1})	Without Zn	101 (5096)	38.2 (38.0, 38.6)	32.9	43.5	47.5	7.1
	With Zn	38 (765)	36.7 (36.1, 37.5)	32.2	42.8	38.2	7.7
Protein (%)	Without Zn	94 (8244)	12.6 (12.6, 12.6)	11.5	13.6	12.3	NA
	With Zn	43 (611)	12.3 (12.2, 12.5)	10.2	13.8	19.7	NA
Phytate (%)	Without Zn	32 (464)	0.89 (0.87, 0.92)	0.74	1.08	49.6	NA
	With Zn	25 (508)	0.84 (0.83, 0.86)	0.73	1.01	52.4	NA

C. Analysis by Zn fertilization and genotypes of bread wheat

Genotype	Zn fertilizer	Number of genotypes	Median (95% CI)‡	Q1	Q3	CV (%)	Proportion with Zn >38 mg kg^{-1}
Landraces	Without Zn	39	47.3 (43.1, 52.9)	39.2	53.4	17.8	82.1
	With Zn	28	58.5 (53.8, 62.8)	50.5	65.5	15.6	100.0
Varieties	Without Zn	673	31.7 (30.7, 32.6)	24.9	39.3	34.7	28.5
	With Zn	283	44.0 (42.5, 45.8)	35.7	52.2	29.8	66.4
Landraces	All	67	51.8 (49.5, 54.5)	45.5	58.5	20.4	89.6
Varieties	All	956	34.8 (33.8, 35.9)	26.9	43.4	36.8	39.7

† Figures in parentheses represent the total number of observations (n) available for analysis

‡ Figures in parentheses are 95% confidence intervals (CIs) of median values. Two medians are deemed significantly different if their 95% CIs do not overlap.

Table S3. G×E interactions

Table S3. The proportion of variation explained (in %) by genotype, environment and the genotype by environment interactions (G×E) in grain yield, gran Zn, Fe and protein concentrations in bread wheat cultivars

Variable	Region/country [G, E]†	Genotype	Environment	GxE	Reference
Grain yield	Ethiopia [11; 4]	8.7*	82.0*	9.3*	Alemayehu et al. (2025)
	Ethiopia [15; 12]	11.3*	52.3*	22.9*	Alemu et al. (2021)
	Ethiopia [180; 2]	27.5*	55.2*	17.4	Atsbeha et al. (2023)
	Ethiopia [30; 7]	2.6*	46.6*	25.4*	Bacha (2015)
	Ethiopia [12; 9]	5.0*	76.5*	18.5*	Bayissa et al. (2023)
	Ethiopia [19; 7]	7.1*	78.3*	14.7*	Mehari et al. (2015)
	Ethiopia [22; 6]	9.0*	52.0*	20.7*	Temesgen et al. (2015)
	Ethiopia [15; 6]	33.5*	35.3*	31.5*	Wardofa et al. (2019)
	India-normal [20; 6]	21.8*	15.4*	30.2*	Khare et al. (2024)
	India-heat stress [20: 6]	19.5*	9.7*	39.8*	Khare et al. (2024)
	India [20; 14]	11.7*	45.1*	28.2*	Joshi et al. (2010)
	India [100; 8]	26.4*	70.2*	3.4*	Gupta et al. (2022)
	India, Pakistan, Mexico [39, 9]	6.1*	85.1*	8.8*	Velu et al. (2012)
	Iran [24; 9]	1.0	83.5	6.5	Roostaei et al. (2022)
	Nepal [20; 3]	5.6*	72.7*	21.8*	Bhandari and Poudel (2023)
	USA [18, 3]	NA*	NA*	NA*	Murphy et al. (2011)
	Uzbekistan [30; 7]	9.5*	63.1*	15.0*	Khazratkulova et al. (2015)
	Global [50, 233]	NA*	NA*	NA*	Velu et al. (2022)
Grain Zn	France [51; 3]	32.4*	44.3*	23.2*	Oury et al. (2006)
	India [309; 3]	16.9ns	57.7*	23.2*	Gopalareddy et al. (2015)
	India [20; 10]	0.50	85.5*	5.8	Joshi et al. (2010)
	India, Mexico [330, 5]	6.0*	67.4*	12.8*	Velu et al. (2016)
	India, Pakistan, Mexico [39, 9]	10.7*	50.9*	20.4*	Velu et al. (2012)
	Kazakhstan-SW [25; 5]	8.7	83.9	7.4	Morgounov et al. (2006)
	Kazakhstan-WWa [5; 2]	8.6	42.7	48.8	Morgounov et al. (2006)
	Kazakhstan-WWb [5; 2]	4.7	61.7	33.6	Morgounov et al. (2006)
	Kyrgyzstan-WW [10; 3]	32.6	42.2	25.2	Morgounov et al. (2006)
	Tajikistan-WW [22; 3]	35.1	31.8	33.1	Morgounov et al. (2006)
	USA [18, 3]	NA*	NA*	NA*	Murphy et al. (2011)
Grain Fe	France [51; 3]	30.7	24.9	44.4*	Oury et al. (2006)
	France [11; 3]	39.3	24.2	36.5*	Oury et al. (2006)
	India [309; 3]	27.1*	37.4*	29.5*	Gopalareddy et al. (2015)
	India [20; 10]	3.3	37.2*	32.6	Joshi et al. (2010)
	India [140; 4]	40.3*	13.4*	20.6*	Tiwari et al. (2016)
	India, Mexico [330; 5]	0.2	99.3*	0.2	Velu et al. (2016)
	India, Pakistan, Mexico [39, 9]	20.9*	41.5*	22.1*	Velu et al. (2012)
	Kazakhstan-SW [25; 4]	50.5	31.6	17.9	Morgounov et al. (2006)
	Kazakhstan-WWa [5; 2]	51.3	9.9	38.8	Morgounov et al. (2006)
	Kazakhstan-WWb [5; 2]	22.8	28.5	48.6	Morgounov et al. (2006)
	Kyrgyzstan-WW [10; 3]	22.2	50.6	27.2	Morgounov et al. (2006)
	Tajikistan-WW [22; 3]	22.7	42.1	35.2	Morgounov et al. (2006)
	USA [18, 3]	NA*	NA*	NA*	Murphy et al. (2011)
Protein	France [51; 3]	20.1	70.1	NA	Oury et al. (2006)
	India [140; 4]	25.3*	43.6*	16.3*	Tiwari et al. (2016)
	Uzbekistan [30; 7]	9.0*	45.0*	33.7*	Khazratkulova et al. (2015)

† C and E represent the number of cultivars and environments in the study; * represents significant effect ($P < 0.05$) of genotype, environment or the GxE interaction

NA = estimate and significance level not available; NA* = significance level reported without estimate

Table S4. Heritability of traits

Table S4. Broad-sense heritability of grain yield, gran Zn, Fe and protein concentrations of bread wheat cultivars reported by various authors

Variable	Region/country [G, E][†]	Wheat	Heritability	Reference
Grain yield	Ethiopia [180; 2]	Diverse	0.29	Atsbeha et al. (2023)
	Ethiopia [30; 2]	Diverse	0.52	Bayisa et al. (2020)
	Iran [80, 2]	Diverse	0.57-0.66	Amiri et al. (2018)
	Nepal [50; 20]	Diverse	0.20-0.57	Thapa et al. (2022)
	Nepal [50; 1]	Biofortified	0.92	Pant et al. (2020)
	Siberia [135; 1]	Diverse	0.82	Shepelev et al. (2022)
	Global [50, 233]	Diverse	0.62-0.83	Velu et al. (2022)
Grain Zn	Kazakhstan and Russia [18; 6]	Spring wheat	0.44	Morgounov et al. (2022)
	China [166; 4]	Diverse	0.71	Tong et al. (2022)
	India [309; 3]	Diverse	0.71	Gopalareddy et al. (2015)
	India [20; 10]	Diverse	0.25	Joshi et al. (2010)
	India, Mexico [330, 5]	Diverse	0.47	Velu et al. (2016)
	India, Pakistan, Mexico [39, 9]	Diverse	0.78	Velu et al. (2012)
	India [140; 4]	Diverse	0.53	Tiwari et al. (2016)
	India [34; 6]	Biofortified	0.41-0.99	Khokhar et al. (2018)
	India [280; 5]	Diverse	0.33-0.85	Krishnappa et al. (2022)
	India [184; 3]	Diverse	0.50–0.88	Rathan et al. (2022)
	Iran [30, 1]	Spring wheat	0.61-0.92	Khodadadi et al. (2014)
	Iran [80, 2]	Diverse	0.31-0.50	Amiri et al. (2018)
	Nepal [50; 20]	Diverse	0.52-0.81	Thapa et al. (2022)
	Nepal [50; 1]	Biofortified	0.58	Pant et al. (2020)
	Siberia [135; 1]	Spring wheat	0.27	Shepelev et al. (2022)
	Global [20, 14]	Diverse	0.74-0.88	Velu et al. (2022)
Grain Fe	Kazakhstan and Russia [18; 6]	Spring wheat	0.38	Morgounov et al. (2022)
	China [166; 4]	Diverse	0.72	Tong et al. (2022)
	India [309; 3]	Diverse	0.81	Gopalareddy et al. (2015)
	India [20; 10]	Diverse	0.37	Joshi et al. (2010)
	India, Mexico [330; 5]	Diverse	0.67	Velu et al. (2016)
	India, Pakistan, Mexico [39, 9]	Diverse	0.88	Velu et al. (2012)
	India [140; 4]	Diverse	0.72	Tiwari et al. (2016)
	India [34; 6]	Biofortified	0.22-0.99	Khokhar et al. (2018)
	India [280; 5]	Diverse	0.45-0.90	Krishnappa et al. (2022)
	India [184; 3]	Diverse	0.40–0.80	Rathan et al. (2022)
	Iran [30, 1]	Spring wheat	0.74-0.85	Khodadadi et al. (2014)
	Iran [80, 2]	Diverse	0.44-0.53	Amiri et al. (2018)
	Nepal [50; 20]	Diverse	0.15-0.79	Thapa et al. (2022)
	Nepal [50; 1]	Biofortified	0.55	Pant et al. (2020)
	Siberia [135; 1]	Spring wheat	0.58	Shepelev et al. (2022)
	Global [20, 14]	Diverse	0.69-0.92	Velu et al. (2022)
Protein	India [140; 4]	Diverse	0.78	Tiwari et al. (2016)
	Iran [80, 2]	Diverse	0.76-0.80	Amiri et al. (2018)
	Siberia [135; 1]	Spring wheat	0.86	Shepelev et al. (2022)
	Germany [369;]	Diverse	0.80	Alomari et al. (2023)
	Germany [255;]	Winter wheat	0.68–0.79	Kartseva et al. (2023)
	France & Germany [372; 8]	Diverse	0.91	Muqaddasi et al. (2020)
	India [184; 3]	Diverse	0.56–0.82	Rathan et al. (2022)
	Kazakhstan and Serbia [42, 9]	Spring wheat	0.59-0.87	Gómez-Becerra et al. (2010)

[†] C and E represent the number of cultivars and environments in the study; * represents significant effect ($P < 0.05$) of genotype, environment or the GxE interaction

NA = estimate and significance level not available; NA* = significance level reported without estimate

SW = spring wheat; WW = winter wheat

Table S5. Correlation between traits

Table S5. Pearson correlation coefficients between wheat grain yield, grain zinc, iron, protein and phytate concentrations (top) and the percentage of studies (bottom) from which significantly positive, negative and non-significant correlation coefficients were found among those focussing on germplasm evaluation and fertilization

Pearson correlation coefficients (top)

		Grain yield	Zinc	Iron	Protein
Bread	Zinc	-0.003			
	Iron	-0.014	0.533***		
	Protein	-0.080***	0.140***	0.298***	
	Phytate	0.150***	-0.124**	-0.226 ***	0.060
Durum	Zinc	0.278***			
	Iron	0.073	0.087*		
	Protein	0.208***	-0.097*	0.248***	
	Phytate	0.320**	-0.237**	-0.602***	0.290**
Spelt	Zinc	-0.431			
	Iron	0.489*	0.098*		
	Protein	0.081	0.800***	0.519***	
	Phytate	NA	0.404***	0.092	NA

Percentage of studies (bottom)

Study type	Correlations	Number of studies	Significantly positive (in %)	Significantly negative (in %)	Non-significant (in %) correlations
Germplasm	Grain vs Zn	19	10.5	31.6	57.9
	Grain vs Fe	13	0.0	30.8	69.2
	Grain vs protein	13	23.1	61.5	15.4
	Zn vs Fe	30	73.3	0.0	26.7
	Zn vs protein	11	72.7	0.0	27.3
	Zn vs phytate	6	50.0	16.7	33.3
	Fe vs protein	11	54.5	0.0	45.5
	Fe vs phytate	5	20.0	0.0	80.0
Fertilization	Grain vs Zn	59	40.7	28.8	30.5
	Grain vs Fe	22	22.7	18.2	59.1
	Grain vs protein	31	38.7	25.8	35.5
	Zn vs Fe	22	50.0	9.1	40.9
	Zn vs protein	26	65.4	3.8	30.8
	Zn vs phytate	12	0.0	25.0	75.0
	Fe vs protein	18	22.2	11.1	66.7

Statistical significance : * $\alpha = 0.05$; ** $\alpha = 0.001$; *** $\alpha = 0.0001$

NA = Not available

Table S6. Number of studies included in meta-analysis

Table S6. The total number of studies (N) and total number of observations (n) for treatments with and without Zn and Fe application and all others inputs. All have NP or NPK as the basic treatment.

Treatment	Study (n)	Treatment	Study (n)
**With Zinc (Zn) or iron (Fe)**		**Other treatments continued**	
Zn foliar	69 (815)	Mn foliar	1 (3)
Zn soil	66 (647)	Mn soil	1 (2)
Zn seed coating	7 (45)	N alone	5 (44)
Zn soil + foliar	28 (341)	N+ K	1 (2)
Zn + Fe foliar	9 (83)	B	1 (4)
Zn + Fe soil	6 (116)	lime	1 (9)
Zn + Fe soil + foliar	2 (13)	lime +OM	1 (6)
Fe foliar	15 (166)	mycorrhiza	1 (4)
Fe soil	4 (12)	OM	3 (14)
Fe seed coating	1 (8)	Si	1 (10)
**Other treatments**		NPK high	14 (78)8
No input control	15 (75)	NPK low	12 (53)
Biochar	1 (1)	P alone	2 (26)
B soil	1 (4)	P + K	3 (22)
Compost	1 (1)	Se foliar	2 (6)
Cu foliar	1 (1)	Se soil	1 (4)
I foliar	1 (27)	S soil	1 (5)
Mg soil	1 (3)		

NPK is a shorthand for the recommended nitrogen, phosphorus and potassium fertilizer
In other treatments, B, Cu, I, Mg, Mn, Se and S represent boron, copper, iodine, magnesium, manganese, selenium and sulphur, respectively.

Table S7. Treatment effect on response ratios

Table 7. Variations in the response ratios of grain yield, grain zinc (Zn), iron (Fe), protein, and phytate concentrations across treatments in bread wheat cultivars

	Response ratios				
Treatments	Grain yield	Grain Zn	Grain Fe	Grain protein	Grain phytate
No-input	0.81 (0.76–0.86)	1.02 (0.96–1.07)	1.04 (0.97–1.12)	0.84 (0.76–0.92)	--
NPK+Others[†]	1.03 (1.01–1.06)	1.06 (1.04–1.07)	1.06 (1.01–1.11)	1.01 (0.98–1.04)	0.92 (0.84–1.00)
NPK+Zn foliar	1.09 (1.08–1.11)	1.54 (1.50–1.57)	1.14 (1.10–1.17)	1.10 (1.05–1.15)	0.96 (0.94–0.98)
NPK+Zn soil	1.15 (1.13–1.16)	1.32 (1.29–1.35)	1.05 (1.03–1.07)	1.21 (1.17–1.24)	0.95 (0.93–0.96)
NPK+Zn seed	1.19 (1.11–1.27)	1.26 (1.12–1.41)	NA	NA	NA
NPK+Zn soil+Zn foliar	1.09 (1.07–1.11)	1.65 (1.59–1.71)	0.99 (0.96–1.02)	1.19 (1.14–1.24)	0.95 (0.91–0.98)
NPK+Zn foliar+Fe foliar	1.08 (1.04–1.12)	1.29 (1.22–1.37)	1.17 (1.13–1.21)	1.19 (1.10–1.28)	NA
NPK+Zn soil+Fe soil	1.26 (1.22–1.30)	1.27 (1.23–1.31)	1.41 (1.37–1.45)	1.25 (1.13–1.37)	NA
NPK+Fe foliar	1.07 (1.04–1.10)	1.17 (1.06–1.30)	1.13 (1.09–1.17)	1.18 (1.11–1.26)	0.98 (0.96–1.00)
NPK+Fe soil	1.13 (1.08–1.18)	1.12 (1.08–1.15)	1.48 (1.44–1.52)	1.23 (1.08–1.38)	NA

[†] "Others" represents all treatments other than Zn and Fe inputs (see Supplementary Table S6 for details)

NA indicates that effect sizes could not be estimated due to small sample sizes (see Supplementary Table S6). Data are presented as mean values and 95% confidence intervals (CIs). Figures in parentheses are CIs of means. Effect sizes are deemed significantly larger than those of NPK fertilizers if their CIs fall above 1, but not significantly different from those of NPK control if the CIs encompass 1.

Table S8. Correlation between response ratios

Table 8. Pearson correlation coefficients between response ratios of grain yield, grain Zn, iron, protein and phytate concentrations in bread wheat with application rates of N, P, Zn and Fe and select soil properties

	Response ratios of				
Variable	Grain yield (1941)	Zn (2123)	Fe (906)	Protein (734)	Phytate (463)
**Nutrient application rates**					
N rate (2361)	0.136***	0.069**	0.207***	0.293***	-0.048
P rate (2278)	0.105***	0.075**	0.097**	0.260**	-0.108*
K rate (1669)	-0.025	-0.101**	-0.120**	0.235***	-0.075
Zn rate (881)	0.020	0.174***	-0.035	0.201***	0.254***
Fe rate (237)	0.368***	-0.507***	0.380***	0.094	NA
**Soil variables**					
Sand (1960)	0.045	0.140***	-0.093	0.103	0.139**
Clay (1960)	-0.010	0.025	0.049	-0.168**	-0.148**
Soil pH (2024)	0.067*	0.007	-0.042	0.038	0.095
SOC (2004)	-0.073	0.008	0.101*	-0.092	0.143**
Total N (1061)	0.124***	0.133***	0.168***	0.092	-0.136*
Olsen P (2039)	0.099***	0.024	0.057	-0.027	0.045
Available K (836)	-0.074	0.170***	0.251***	-0.208**	0.014
Soil Zn (1640)	-0.040	0.148***	0.059	-0.060	0.463***
Soil Fe (751)	-0.045	-0.066	0.047	0.078	0.502***

Figures in parentheses are the total numbers of observations available for each variable

Statistical significance: * $\alpha = 0.05$; ** $\alpha = 0.001$; *** $\alpha = 0.0001$

NA = Not available

Table S9 Comparison of irrigated with rainfed wheat

Table S9. Variations in response ratios of grain yield, grain Zn, Fe, protein and phytate concentrations with the interactive effect of irrigation with treatments in bread wheat

		Response ratios (95% CI)†				
Treatments	Irrigation	Grain yield	Zinc	Iron	Protein	Phytate
Overall	Irrigated	1.11 (1.10–1.12)	1.33 (1.31–1.35)	1.11 (1.09–1.13)	1.21 (1.18–1.23)	0.96 (0.94–0.97)
	Rainfed	1.06 (1.05–1.07)	1.43 (1.40–1.46)	1.16 (1.13–1.19)	1.06 (1.03–1.08)	0.95 (0.93–0.97)
Zn alone	Irrigated	1.12 (1.11–1.14	1.37 (1.35–1.40)	1.08 (1.06–1.10)	1.21 (1.18–1.24)	0.95 (0.92–0.97)
	Rainfed	1.10 (1.08–1.12)	1.20 (1.19–1.22)	1.07 (1.05–1.09)	1.14 (1.10–1.17)	0.95 (0.94–0.97)
Fe alone	Irrigated	1.09 (1.06–1.12)	1.12 (1.05–1.19)	1.22 (1.18–1.26)	1.30 (1.20–1.39)	0.98 (0.96–1.00)
	Rainfed	1.05 (1.01–1.08)	1.23 (0.99–1.49)	1.28 (1.19–1.36)	1.09 (1.04–1.15)	NA
Zn + Fe	Irrigated	1.11 (1.07–1.15)	1.27 (1.20–1.35)	1.21 (1.16–1.26)	1.20 (1.13–1.28)	NA
	Rainfed	1.08 (1.02–1.16)	1.28 (1.24–1.32)	1.37 (1.33–1.41)	NA	NA
Others	Irrigated	1.08 (1.03–1.12)	1.09 (1.06–1.13)	1.02 (1.00–1.04)	1.16 (1.08–1.24)	0.85 (0.76–0.94)
	Rainfed	1.00 (0.98–1.02)	1.03 (1.02–1.05)	1.07 (1.01–1.14)	0.96 (0.94–0.99)	1.08 (0.98–1.18)

† Figures in parentheses are 95% confidence intervals (CIs) of means. Effect sizes of two or more treatments do not significantly differ if their CIs are overlapping.

NA represents that effect sizes could not be estimated due to small sample sizes or lack of relevant studies.

Supplementary Methods for G×E interactions, heritability and trait correlations

To gain insights into the genetic and environmental control of grain Zn, Fe and protein concentrations in wheat, we reviewed studies that have quantified G×E interactions and heritability of these traits in wheat. From those studies, we compiled estimates of the variance explained by the genotype, environment and the G×E interactions and the broad-sense (H^2) and narrow-sense (h^2) heritability values. The broad-sense heritability is defined as the proportion of phenotypic variance that is attributable to the overall variance for the genotype, thus including additive, dominance, and epistatic variance (Covarrubias-Pazaran, 2019). The narrow-sense heritability is defined as the proportion of phenotypic variance due to additive genetic variation (Evans et al., 2018). To facilitate inferences, heritability was considered low if H^2 <0.40, but medium if H^2 is 0.40-0.59 and high if H^2 > 0.60 following. However, a low heritability does not necessarily mean that the genetic variance is small. Low heritability can indicate that the error variance is large, which can be caused by high environmental influence (Covarrubias-Pazaran, 2019). The size of H^2 depends on genetic variance in a population, the influence of the environment and on the accuracy of observations such as inaccurate phenotype recording or modelling errors. The genetic variance in one population may be (somewhat) different from that in another population. Heritability within a population can also change over time. The heritability estimate is also specific to the population and environment in which the analysis is performed. The studies used in the review of the G×E interactions and heritability have been listed at the end of the methods.

To gain insights into the potential for simultaneous improvement of grain Fe, Zn and protein with grain yield through either agronomic practices or traditional breeding techniques, we examined the correlations between grain yield, grain Zn, Fe, protein and phytate concentrations. Grain yield and grain protein concentration have been two major targets in wheat breeding programmes as the former determines crop productivity and the latter is a key determinant of the end-use value and

baking quality wheat (Bogard et al., 2010). However, grain yield and grain protein concentration have been difficult to improve simultaneously due to the negative genetic relationship between them. It is not yet clear how this negative correlation affects grain Zn and Fe concentrations when all four variables are considered together. Therefore, we performed correlation analyses between grain yield, grain Zn, Fe and protein concentrations using the data compiled for the meta-analysis. We limited the correlation analysis to studies that have reported two target variables concurrently and where the reported number of means was at least 10 to correctly estimate the Pearson correlation coefficient. We performed the correlation analysis separately for studies that evaluated wheat germplasm and those studies that evaluated fertilization treatments. Then we calculated the proportion (in%) of studies that resulted in significantly positive, significantly negative and non-significant correlation coefficients. We judged the significance of the correlation coefficients using both the P values and the 95% confidence limits. A correlation coefficient was deemed significant if $P<0.05$ and the 95% confidence interval does not encompass 0. The type of study, the variables correlated, the data source or the specific study, country (and the total number of observations), the estimated correlation coefficients, the two-tailed P value and the statistical significance of each correlation have been presented in a table just before additional references.

Studies used in the review of G × E interactions and heritability

Alemayehu L, Kebede M, Wada E (2025) AMMI analysis of elite bread wheat (*Triticum aestivum* L.) selections for genotype by environment interaction and stability of grain yield in Southern Ethiopia. PLoS ONE 20(1): e0318559. DOI: 10.1371/journal.pone.0318559

Alemu G, Dabi A, Geleta N, Duga R, Solomon T, Zegaye H, Getamesay A, Delesa A, Asnake D, Asefa B, Shewaye Y, Abeyo GB, Badebo A (2021) Genotype × Environment Interaction and Selection of High Yielding Wheat Genotypes for Different Wheat-growing Areas of Ethiopia. American Journal of BioScience 9(2): 63-71. DOI: 10.11648/j.ajbio.20210902.15.

Alomari DZ, Schierenbeck M, Alqudah AM, Alqahtani MD, Wagner S, Rolletschek H, Borisjuk L, Röder MS. Wheat Grains as a Sustainable Source of Protein for Health. *Nutrients*. 2023; 15(20):4398. DOI: 10.3390/nu15204398

Amiri R, Bahraminejad S, Cheghamirza K (2018) Estimating genetic variation and genetic parameters for grain iron, zinc and protein concentrations in bread wheat genotypes grown in Iran. Journal of Cereal Science 80: 16e23. DOI: 10.1016/j.jcs.2018.01.009

Atsbeha G, Mekonnen T, Kebede M, Haileselassie T, Tesfaye K (2023) Multivariate analyses, heritability and genotype environment interaction of bread wheat genotypes in Ethiopia. Ecological Genetics and Genomics 29: 100209. DOI: 10.1016/j.egg.2023.100209.

Bayisa T, Habtamu Tefera, Tesfaye Letta. Genetic Variability, Heritability and Genetic Advance Among Bread Wheat Genotypes at Southeastern Ethiopia. Agriculture, Forestry and Fisheries 9: 128-134. doi: 10.11648/j.aff.20200904.15.

Bacha T (2015) Genotype X Environment Interaction and Yield Stabliity of Breade Wheat (Triticum Eastivum L.) Genotype in Ethiopia using the Ammi Analysis. Journal of Biology, Agriculture and Healthcare 5(11): 129-139.

Bayissa T, Mengistu G, Gerema G, Balcha U, Feyisa H, Kedir A, Legese Z, Asegid A, Leta T, Jobe T (2023) Genotype × environment interaction of lowland bread wheat varieties for irrigation in different areas of Oromia. Plant-Environment Interactions 4:2–10. DOI: 10.1002/pei3.10097

Bhandari R, Poudel MR (2023) Genotype × environment interaction and selection parameters for high yielding wheat genotypes under irrigated and heat stress environment. J Sustain Agric Environ. 2024;3:e12098. DOI: 10.1002/sae2.12098.

Breiman, L., 2001. Random forests. Mach. Learn. 45, 5–32.

Breiman, L., Cutler, A., 2012. Breiman and Cutler’s random forests for classification and regression. Packag. “randomForest” 29. https://doi.org/10.5244/C.22.54

Gómez-Becerra HF, Abugalieva A, Morgounov A, Abdullayev K, Bekenova L, Yessimbekova M et al. (2010) Phenotypic correlations, G *3* E interactions and broad sense heritability analysis of grain and flour quality characteristics in high latitude spring bread wheats from Kazakhstan and Siberia. Euphytica 171: 23–38. DOI 10.1007/s10681-009-9984-6

Gopalareddy, K., Singh, A.M., Ahlawat, A.K., Singh, G.P., and Jaiswal, J.P. (2015) Genotype-environment interaction for grain iron and zinc concentration in recombinant inbred lines of a bread wheat (*Triticum aestivum* L.) cross. *Indian J. Genet.*, 75(3): 307-313. DOI: 10.5958/0975-6906.2015.00048.6.

Gupta, V.; Kumar, M.; Singh, V.; Chaudhary, L.; Yashveer, S.; Sheoran, R.; Dalal, M.S.; Nain, A.; Lamba, K.; Gangadharaiah, N.; et al. (2022) Genotype by environment interaction analysis for grain yield of wheat (Triticum aestivum (L.) em.Thell) Genotypes. Agriculture 12, 1002. DOI: 10.3390/agriculture12071002

Joshi A.K., Crossa J, Arun B, Chand R, Trethowan R, Vargas M, Ortiz-Monasterio, I (2010) Genotype x environment interaction for zinc and iron concentration of wheat grain in eastern Gangetic plains of India. Field Crops Research 116: 268–277. doi:10.1016/j.fcr.2010.01.004.

Kartseva, T.; Alqudah, A.M.; Aleksandrov, V.; Alomari, D.Z.; Doneva, D.; Arif, M.A.R.; Börner, A.; Misheva, S. (2023) Nutritional genomic approach for improving grain protein content in wheat. Foods 12, 1399. DOI: 10.3390/foods12071399

Khodadadi, M., Hamid, D., Mohammad, H.F., 2014. Heritability and genetic diversity of iron, zinc and some morphological and physiological traits in some spring wheat genotypes (Triticum aestivum L.). Int. J. Biosci. 4, 1e9.

Khokhar JS, Sareen S, Tyagi BS, Singh G, Wilson L, King IP, et al. (2018) Variation in grain Zn concentration, and the grain ionome, in field-grown Indian wheat. PLoS ONE 13(1): e0192026. DOI: 10.1371/journal.pone.0192026.

Khare V, Shukla RS, Pandey S, Singh SK, Singh C (2024) Exploring the genotype-environment interaction of bread wheat in ambient and high-temperature planting conditions: a rigorous investigation. Scientific Reports 14: 2402. DOI: 10.1038/s41598-024-53052-w

Khazratkulova S, Sharma Rc, Amanov A, Ziyadullaev Z, Amanov O, Alikulov S, Ziyaev Z, Muzafarova D (2015) Genotype × environment interaction and stability of grain yield and selected quality traits in winter wheat in Central Asia. Turkish Journal of Agriculture and Forestry 39: 920-929. doi:10.3906/tar-1501-24.

Krishnappa G, Khan H, Krishna H, Kumar S, Mishra CN, Parkash O, Devate NB, Nepolean T et al (2022) Genetic dissection of grain iron and zinc, and thousand kernel weight in wheat (*Triticum aestivum* L.) using genome-wide association study. Scientific Reports 12:12444. DOI: 10.1038/s41598-022-15992-z

Mehari M, Tesfay M, Yirga H, Mesele A, Abebe T, Workineh A, Amare B (2015) GGE biplot analysis of genotype-by-environment interaction and grain yield stability of bread wheat genotypes in South Tigray, Ethiopia. Communications In Biometry And Crop Science 10: 17–26.

Morgounov, A., Gómez-Becerra, H.F., Abugalieva, A, Dzhunusova M, Yessimbekova M, Muminjanov H, Zelenskiy Y, Ozturk L, Cakmak I. (2007) Iron and zinc grain density in common wheat grown in Central Asia. *Euphytica* **155**, 193–203. DOI: 10.1007/s10681-006-9321-2.

Morgounov A, Savin T, Flis P, Babkenov A, Chudinov V, Kazak A, Koksel H, Likhenko I, Sharma R, Shelaeva T, et al. (2022) Effects of environments and cultivars on grain ionome of spring wheat grown in Kazakhstan and Russia. Crop and Pasture Science 73: 515–527. doi:10.1071/CP21493.

Muhder, N., Gessese, M. K. and Sorsa, Z., 2020. Assessment of genetic variability among agronomic traits and grain protein content of elite bread wheat (Triticum aestivum L.) genotypes in the central highlands of Ethiopia. Asian J. Agric. Res., 14: 1-12.

Muqaddasi, Q.H.; Brassac, J.; Ebmeyer, E.; Kollers, S.; Korzun, V.; Argillier, O.; Stiewe, G.; Plieske, J.; Ganal, M.W.; Röder, M.S. (2020**)** Prospects of GWAS and predictive breeding for european winter wheat's grain protein content, grain starch content, and grain hardness. Scientific Reports 10: 12541. DOI: 10.1038/s41598-020-69381-5.

Murphy KM, Hoagland, LA, Yan, L, Colley, M, Jones, SJ (2011) Genotype × environment interactions for mineral concentration in grain of organically grown spring wheat. Agronomy Journal 103: 1734–1741. doi:10.2134/agronj2011.0097.

Oury, FX, Leenhardt, F., Rémésy, C, Chanliaud, E, Duperrier B, Balfourier F, Charmet G (2006) Genetic variability and stability of grain magnesium, zinc and iron concentrations in bread wheat. *European Journal of Agronomy* 25 (2), pp.177-185. DOI: 10.1016/j.eja.2006.04.011.

Pant KR, Ojha BR, Thapa DB, Kharel R, Gautam NJ, Shrestha J. (2020) Evaluation of biofortified spring wheat genotypes for yield and micronutrient contents. Fundamental and Applied Agriculture 5(1): 78–87. DOI: 10.5455/faa.79404.

Rathan, N.D.; Krishna, H.; Ellur, R.K.; Sehgal, D.; Govindan, V.; Ahlawat, A.K.; Krishnappa, G.; Jaiswal, J.P.; Singh, J.B.; Sv, S.; et al. (2022) Genome-wide association study identifies loci and candidate genes for grain micronutrients and quality traits in wheat (Triticum aestivum L.). Sci. Rep. 12, 7037. DOI: 10.1038/s41598-022-10618-w

Rawat, N., Tiwari, V.K., Singh, N., Randhawa, G.S., Singh, K., Chhuneja, P., Dhaliwal, H.S., 2009. Evaluation and utilization of Aegilops and wild Triticum species for enhancing iron and zinc content in wheat. Genet. Resour. Crop Evol. 56, 53e64.

Roostaei M, Jafarzadeh J, Roohi E, et al. Genotype × environment interaction and stability analyses of grain yield in rainfed winter bread wheat. *Experimental Agriculture*. 2022;58:e37. doi:10.1017/S0014479722000345.

Seyoum EG, Sisay A. (2021) Genetic variability, heritability and genetic advance study in bread wheat genotypes (*Triticum aestivum* L.). Advances in Bioscience and Bioengineering 9: 81-86. DO: 10.11648/j.abb.20210903.13

Shepelev, S., Morgounov, A.; Flis, P.; Koksel, H., Li, H.; Savin, T.; Sharma, R.; Wang, J.; Shamanin, V. (2022) Variation of Macro- and Microelements, and Trace Metals in Spring Wheat Genetic Resources in Siberia. Plants 11: 149. DOI: 10.3390/plants11020149.

Temesgen M, Alamerew S, Eticha F, Mehari M (2015) Genotype X Environment Interaction and Yield Stability of Bread Wheat Genotypes in South East Ethiopia. World Journal of Agricultural Sciences 11: 121-127. DOI: 10.5829/idosi.wjas.2015.11.3.1837

Thapa DB, Subedi M, Yadav RP, Joshi BP, Adhikari BN, Shrestha KP, Magar PB, Pant KR, Gurung SB, Ghimire S, Gautam NR, Acharya NR, Sapkota M, Mishra VK, Joshi AK, Singh RP and Govindan V (2022) Variation in Grain Zinc and Iron Concentrations, Grain Yield and Associated Traits of Biofortified Bread Wheat Genotypes in Nepal. Front. Plant Sci. 13:881965. doi: 10.3389/fpls.2022.881965

Tiwari, C., Wallwork, H., Arun, B., Mishra, V.K., Velu, G., Stangoulis, J., Kumar, U., Joshi, A.K., (2016) Molecular mapping of quantitative trait loci for zinc, iron and protein content in the grains of hexaploid wheat. Euphytica 207, 563e570.

Tong J, Zhao C, Sun M, Fu L, Song J, Liu D, Zhang Y, Zheng J, Pu Z, Liu L, Rasheed A, Li M, Xia X, He Z and Hao Y (2022) High resolution genome wide association studies reveal rich genetic architectures of grain zinc and iron in common wheat (*Triticum aestivum* L.). Front. Plant Sci. 13:840614. doi: 10.3389/fpls.2022.840614.

Velu, G., Singh, R. P., Huerta-Espino, J., Peña, R. J., Arun, B., Mahendru-Singh, A., Mujahid, M. Y., Sohu, V. S., Mavi, G. S., Crossa, J., Alvarado, G., Joshi, A. K.,& Pfeiffer, W. H. (2012). Performance of biofortified spring wheat genotypes in target environments for grain zinc and iron concentrations. Field Crops Research, 137: 261–267. DOI: 10.1016/j.fcr.2012.07.018.

Velu G, Atanda S, Singh RP, Huerta-Espino J, Crespo-Herrera LA, Juliana P, Mondal S, Joshi AK, Bentley AR (2022) Breeding increases grain yield, zinc, and iron, supporting enhanced wheat biofortification. *Crop Science* 62: 1912–1925. DOI: 10.1002/csc2.20759.

Wardofa GA, Mohammed H, Asnake D, Alemu T (2019) Genotype x environment interaction and yield stability of bread wheat genotypes in central Ethiopia. J. Plant Breed. Genet. 07: 87-94. DOI: 10.33687/pbg.007.02.2847.

Studies included in the correlation analysis

The type of study, the variables correlated, the data source, country (number of observations), the Pearson correlation coefficient, the t**wo-tailed** P value and statistical significance of each correlation.

Study type	Variables	Data source (study)	Country (N)	Corelation coefficient	P-value	Significance
Germplasm	Fe vs phytate	Petrovi´c et al. (2020)	Croatia (88)	0.308	0.0018	**
Germplasm	Fe vs phytate	Rehman et al. (2018)	Pakistan (112)	0.116	0.2216	ns
Germplasm	Fe vs phytate	Kariithi et al. (2016)	Kenya (27)	-0.109	0.6011	ns
Germplasm	Fe vs phytate	Bilgrami et al. (2017)	Iran (17)	-0.240	0.3461	ns
Germplasm	Fe vs phytate	Huertas-Garcia et al. (2023)	Spain (36)	-0.340	0.0272	*
Germplasm	Fe vs protein	Gómez-Becerra et al. (2010)	Kazakhstan/Serbia [42]	0.640	0.0000	***
Germplasm	Fe vs protein	Feng et al. (2011)	China (32)	0.524	0.0001	**
Germplasm	Fe vs protein	Velu et al. (2019)	Mexico (85)	0.393	0.0000	***
Germplasm	Fe vs protein	Zhang et al. (2010b)	China (38)	0.357	0.0154	*
Germplasm	Fe vs protein	Khan et al. (2023)	Bangladesh (288)	0.353	0.0000	***
Germplasm	Fe vs protein	Chaudhari et al. (2022)	India (110)	0.266	0.0031	**
Germplasm	Fe vs protein	Johansson et al. (2021)	Sweden (33)	0.255	0.1354	ns
Germplasm	Fe vs protein	Rehman et al. (2018)	Pakistan (112)	0.139	0.1392	ns
Germplasm	Fe vs protein	Vazquez et al. (2018)	Spain (203)	0.102	0.1453	ns
Germplasm	Fe vs protein	Petrovi´c et al. (2020)	Croatia (88)	0.059	0.5973	ns
Germplasm	Fe vs protein	Gomez-Coronado et al. (2018)	Portugal (36)	0.010	0.9583	ns
Germplasm	Grain vs protein	Basshi & Nahapetian (1977)	Iran (20)	0.524	0.0030	**
Germplasm	Grain vs protein	Gautam et al. (2020)	India (123)	0.227	0.0087	**
Germplasm	Grain vs protein	Rehman et al. (2018)	Pakistan (112)	0.205	0.0253	*
Germplasm	Grain vs protein	Bloom et al (2021)	USA (6508)	-0.144	0.0000	***
Germplasm	Grain vs protein	Johansson et al. (2021)	Sweden (33)	-0.178	0.3193	ns
Germplasm	Grain vs protein	Feng et al. (2011)	China (32)	-0.236	0.1793	ns
Germplasm	Grain vs protein	Vazquez et al. (2018)	Spain (203)	-0.304	0.0000	***
Germplasm	Grain vs protein	Gómez-Becerra et al. (2010)	Kazakhstan/Serbia [42, 9]	-0.410	0.0021	**
Germplasm	Grain vs protein	Velu et al. (2019)	Mexico (85)	-0.486	0.0000	***
Germplasm	Grain vs protein	Gomez-Coronado et al. (2018)	Portugal (36)	-0.770	0.0000	***
Germplasm	Grain vs protein	Loes et al. (2020)	Norway (48)	-0.838	0.0000	***
Germplasm	Grain vs protein	Morgounov et al. (2013)	Siberia (36)	-0.855	0.0000	***
Germplasm	Grain vs protein	Jablonskytė-Raščė et al. (2013)	Lithuania (18)	-0.928	0.0000	***
Germplasm	Grain vs Fe	Velu et al. (2019)	Mexico (85)	0.095	0.3922	ns
Germplasm	Grain vs Fe	Gomez-Coronado et al. (2018)	Portugal (36)	0.010	0.9583	ns
Germplasm	Grain vs Fe	Feng et al. (2011)	China (32)	-0.011	0.9570	ns
Germplasm	Grain vs Fe	Rehman et al. (2018)	Pakistan (112)	-0.034	0.7354	ns
Germplasm	Grain vs Fe	Joshi et al. (2010)	India (202)	-0.047	0.5168	ns
Germplasm	Grain vs Fe	Tadesse et al. (2023)	Morocco (242)	-0.049	0.4564	ns
Germplasm	Grain vs Fe	Vazquez et al. (2018)	Spain (203)	-0.060	0.4016	ns
Germplasm	Grain vs Fe	Khokhar et al. (2018)	India (628)	-0.092	0.0202	*
Germplasm	Grain vs Fe	Johansson et al. (2021)	Sweden (33)	-0.348	0.0299	*
Germplasm	Grain vs Fe	Basshi & Nahapetian (1977)	Iran (20)	-0.351	0.0986	ns

Germplasm	Grain vs Fe	Pant et. al. (2020)	Nepal (50)	-0.424	0.0004	***
Germplasm	Grain vs Fe	Gómez-Becerra et al. (2010)	Kazakhstan/Serbia [42, 9]	-0.490	0.0001	***
Germplasm	Grain vs Fe	Thapa et al. (2022)	Nepal (215)	-0.607	0.0000	***
Germplasm	Grain vs Zn	EL-Bendary et al. (2013)	Egypt (18)	0.853	0.0000	***
Germplasm	Grain vs Zn	Rawat et al. (2009)	India (12)	0.421	0.1248	ns
Germplasm	Grain vs Zn	Khazratkulova et al. (2015)	Uzbekistan (30)	0.314	0.0699	ns
Germplasm	Grain vs Zn	Joshi et al. (2010)	India (202)	0.192	0.0050	**
Germplasm	Grain vs Zn	Singh et al. (2020)	India (48)	0.108	0.4728	ns
Germplasm	Grain vs Zn	Rehman et al. (2018)	Pakistan (112)	0.102	0.2856	ns
Germplasm	Grain vs Zn	Khokhar et al. (2018)	India (628)	0.063	0.1137	ns
Germplasm	Grain vs Zn	Thapa et al. (2022)	Nepal (215)	0.053	0.4475	ns
Germplasm	Grain vs Zn	Feng et al. (2011)	China (32)	0.049	0.8035	ns
Germplasm	Grain vs Zn	Tadesse et al. (2023)	Morocco (242)	-0.010	0.8862	ns
Germplasm	Grain vs Zn	Ghorttapeh et al. (2018)	Iran (26)	-0.147	0.4806	ns
Germplasm	Grain vs Zn	Velu et al. (2019)	Mexico (85)	-0.221	0.0351	*
Germplasm	Grain vs Zn	Basshi & Nahapetian (1977)	Iran (20)	-0.232	0.3166	ns
Germplasm	Grain vs Zn	Gao et al. (2011)	Canada (60)	-0.385	0.0007	***
Germplasm	Grain vs Zn	Vazquez et al. (2018)	Spain (203)	-0.391	0.0000	***
Germplasm	Grain vs Zn	Johansson et al. (2021)	Sweden (33)	-0.407	0.0076	*
Germplasm	Grain vs Zn	Pant et. al. (2020)	Nepal (50)	-0.421	0.0005	***
Germplasm	Grain vs Zn	Gómez-Becerra et al. (2010)	Kazakhstan/Serbia [42, 9]	-0.620	0.0000	***
Germplasm	Grain vs Zn	Gomez-Coronado et al. (2018)	Portugal (36)	-0.640	0.0000	***
Germplasm	Zn vs Fe	Gómez-Becerra et al. (2010)	Kazakhstan/Serbia [42]	0.780	0.0000	***
Germplasm	Zn vs Fe	Zhao et al. (2009)	Global (26)	0.721	0.0000	***
Germplasm	Zn vs Fe	Velu et al. (2017c)	Mexico (20)	0.706	0.0000	***
Germplasm	Zn vs Fe	Khoshgoftarmanesh et al. (2012)	Iran (120)	0.702	0.0000	***
Germplasm	Zn vs Fe	Murphy et al. (2011)	USA (108)	0.697	0.0000	***
Germplasm	Zn vs Fe	Johansson et al. (2021)	Sweden (33)	0.640	0.0000	***
Germplasm	Zn vs Fe	Feng et al. (2011)	China (32)	0.596	0.0000	**
Germplasm	Zn vs Fe	Garvin et al. (2006)	USA (28)	0.574	0.0000	**
Germplasm	Zn vs Fe	Pant et. al. (2020)	Nepal (50)	0.568	0.0000	***
Germplasm	Zn vs Fe	Neelam et al. (2012)	India (27)	0.495	0.0014	***
Germplasm	Zn vs Fe	Amir et al (2018)	Iran (160)	0.488	0.0000	***
Germplasm	Zn vs Fe	Thapa et al. (2022)	Nepal (215)	0.488	0.0000	***
Germplasm	Zn vs Fe	Petrovi´c et al. (2020)	Croatia (88)	0.472	0.0000	***
Germplasm	Zn vs Fe	Khan et al. (2023)	Bangladesh (288)	0.460	0.0000	***
Germplasm	Zn vs Fe	Tahir et al. (2021)	India (28)	0.451	0.0047	**
Germplasm	Zn vs Fe	Khokhar et al. (2018)	India (628)	0.442	0.0000	***
Germplasm	Zn vs Fe	Krishnappa et al. (2022)	India (1400)	0.280	0.0000	***
Germplasm	Zn vs Fe	Velu et al. (2019)	Mexico (85)	0.279	0.0062	**
Germplasm	Zn vs Fe	Chaudhari et al. (2022)	India (110)	0.277	0.0020	**
Germplasm	Zn vs Fe	Huertas-Garcia et al. (2023)	Spain (36)	0.264	0.1029	ns
Germplasm	Zn vs Fe	Vazquez et al. (2018)	Spain (203)	0.220	0.0011	**
Germplasm	Zn vs Fe	Zhang et al. (2010b)	China (38)	0.215	0.1834	ns
Germplasm	Zn vs Fe	Tadesse et al. (2023)	Morocco (242)	0.212	0.0006	***
Germplasm	Zn vs Fe	Joshi et al. (2010)	India (202)	0.183	0.0076	**
Germplasm	Zn vs Fe	Gashu et al. (2021)	Ethiopia (327)	0.128	0.0190	*
Germplasm	Zn vs Fe	Rodehutscord et al. (2016)	Germany (29)	0.096	0.6340	ns
Germplasm	Zn vs Fe	Bilgrami et al. (2017)	Iran (17)	0.090	0.7472	ns
Germplasm	Zn vs Fe	Rehman et al. (2018)	Pakistan (112)	0.038	0.7043	ns

Germplasm	Zn vs Fe	Gomez-Coronado et al. (2018)	Portugal (36)	0.010	0.9583	ns
Germplasm	Zn vs Fe	Kariithi et al. (2016)	Kenya (27)	-0.114	0.5834	ns
Germplasm	Zn vs phytate	Gomez-Coronado et al. (2018)	Portugal (36)	0.860	0.0000	***
Germplasm	Zn vs phytate	Bilgrami et al. (2017)	Iran (17)	0.635	0.0001	***
Germplasm	Zn vs phytate	Huertas-Garcia et al. (2023)	Spain (36)	0.513	0.0001	***
Germplasm	Zn vs phytate	Petrovi´c et al. (2020)	Croatia (88)	0.195	0.0612	ns
Germplasm	Zn vs phytate	Kariithi et al. (2016)	Kenya (27)	0.191	0.3367	ns
Germplasm	Zn vs phytate	Rehman et al. (2018)	Pakistan (112)	-0.211	0.0210	*
Germplasm	Zn vs protein	Gomez-Coronado et al. (2018)	Portugal (36)	0.860	0.0000	***
Germplasm	Zn vs protein	Johansson et al. (2021)	Sweden (33)	0.689	0.0000	***
Germplasm	Zn vs protein	Gómez-Becerra et al. (2010)	Kazakhstan/Serbia [42, 9]	0.520	0.0000	***
Germplasm	Zn vs protein	Feng et al. (2011)	China (32)	0.509	0.0002	***
Germplasm	Zn vs protein	Rehman et al. (2018)	Pakistan (112)	0.500	0.0000	***
Germplasm	Zn vs protein	Velu et al. (2019)	Mexico (85)	0.448	0.0000	***
Germplasm	Zn vs protein	Vazquez et al. (2018)	Spain (203)	0.420	0.0000	***
Germplasm	Zn vs protein	Khan et al. (2023)	Bangladesh (288)	0.410	0.0000	***
Germplasm	Zn vs protein	Chaudhari et al. (2022)	India (110)	0.151	0.1098	ns
Germplasm	Zn vs protein	Petrovi´c et al. (2020)	Croatia (88)	-0.030	0.7942	ns
Germplasm	Zn vs protein	Zhang et al. (2010b)	China (38)	-0.057	0.7481	ns
Fertilization	Fe vs protein	Montoya et. al. (2020)	Spain (15)	0.846	0.0000	***
Fertilization	Fe vs protein	Xia et al. (2020)	China (12)	0.697	0.0001	***
Fertilization	Fe vs protein	Cakmak et.al.(2010)	Turkey (24)	0.643	0.0000	***
Fertilization	Fe vs protein	Ghasemi et al. (2013)	Iran (20)	0.578	0.0004	***
Fertilization	Fe vs protein	Kiran et. al.(2021)	Pakistan (12)	0.385	0.1760	ns
Fertilization	Fe vs protein	Lu et al. (2024)	China (10)	0.309	0.3724	ns
Fertilization	Fe vs protein	Lu et al. (2024)	China (10)	0.149	0.7000	ns
Fertilization	Fe vs protein	Ghasemi et al. (2013)	Iran (20)	0.107	0.6684	ns
Fertilization	Fe vs protein	Qamari et al. (2023)	Iran (42)	0.063	0.7060	ns
Fertilization	Fe vs protein	Niyigaba et al. (2019)	China (38)	-0.008	0.9657	ns
Fertilization	Fe vs protein	El-Dahshouri et al. (2017)	Egypt (16)	-0.013	0.9661	ns
Fertilization	Fe vs protein	Kandil et al. (2017)	Egypt (36)	-0.123	0.4826	ns
Fertilization	Fe vs protein	Hao et al. (2021)	China (279)	-0.123	0.0377	*
Fertilization	Fe vs protein	Gao et al. (2012)	Canada (156)	-0.370	0.0000	**
Fertilization	Fe vs protein	Ramzan et al. (2020)	Pakistan (14)	-0.442	0.0681	ns
Fertilization	Fe vs protein	Souza et al. (2019)	Brazil (10)	-0.453	0.1317	ns
Fertilization	Fe vs protein	Hafeez et al.(2021)	Pakistan (16)	-0.605	0.0006	***
Fertilization	Fe vs protein	Reznick et al. (2021)	Brazil (12)	-0.610	0.0036	**
Fertilization	Grain vs protein	Singh & Sandhu (2021)	India (13)	0.980	0.0000	***
Fertilization	Grain vs protein	Hussan et al. (2021)	Pakistan (18)	0.955	0.0000	***
Fertilization	Grain vs protein	Akram et. al.(2017)	Pakistan	0.896	0.0000	***
Fertilization	Grain vs protein	Maamoun & El-Shazly (2014)	Egypt (32)	0.854	0.0000	***
Fertilization	Grain vs protein	El-Dahshouri et al. (2017)	Egypt (16)	0.851	0.0000	*
Fertilization	Grain vs protein	Biswas et. al.(2015)	India (20)	0.779	0.0000	***
Fertilization	Grain vs protein	Kandil et al. (2017)	Egypt (36)	0.726	0.0000	***
Fertilization	Grain vs protein	Sadhegi et al. (2021)	Iran (16)	0.663	0.0000	**
Fertilization	Grain vs protein	Montoya et. al. (2020)	Spain (15)	0.523	0.0126	*
Fertilization	Grain vs protein	Kiran et. al.(2021)	Pakistan (12)	0.517	0.0340	*
Fertilization	Grain vs protein	Nadeem et. al. (2023)	Pakistan (16)	0.438	0.0503	ns
Fertilization	Grain vs protein	Ma et al. (2018)	China (48)	0.373	0.0037	**
Fertilization	Grain vs protein	Xia et al. (2020)	China (12)	0.362	0.2131	ns
Fertilization	Grain vs protein	Anwar et al. 2021	Pakistan (20)	0.317	0.1471	ns
Fertilization	Grain vs protein	Gomaa et al. (2018)	Egypt (38)	0.302	0.0489	ns
Fertilization	Grain vs protein	Barut et.al.(2017)	Turkey (72)	0.210	0.0676	ns
Fertilization	Grain vs protein	Mosavian et al. (2021)	Iran (36)	0.201	0.2311	ns
Fertilization	Grain vs protein	Souza et al. (2019)	Brazil (10)	0.132	0.7354	ns
Fertilization	Grain vs protein	Hafeez et al.(2021)	Pakistan (16)	-0.129	0.6491	ns
Fertilization	Grain vs protein	Gomez-Coronado et. al.(2017)	Portugal (30)	-0.133	0.4915	ns
Fertilization	Grain vs protein	Cakmak et.al.(2010)	Turkey (24)	-0.199	0.3479	ns
Fertilization	Grain vs protein	Ramzan et al. (2020)	Pakistan (14)	-0.370	0.1556	ns
Fertilization	Grain vs protein	Gao et al. (2012)	Canada (156)	-0.373	0.0000	***
Fertilization	Grain vs protein	Qamari et al. (2023)	Iran (42)	-0.401	0.0029	**
Fertilization	Grain vs protein	Reznick et al. (2021)	Brazil (12)	-0.472	0.0681	ns

Fertilization	Grain vs protein	Niyigaba et al. (2019)	China (38)	-0.515	0.0000	***
Fertilization	Grain vs protein	Taskin and Gunes (2022)	Turkey (14)	-0.523	0.0168	ns
Fertilization	Grain vs protein	Ghimire et al. (2021)	USA (42)	-0.664	0.0000	***
Fertilization	Grain vs protein	Ning et. al. (2019)	China (18)	-0.693	0.0000	***
Fertilization	Grain vs protein	Lu et al. (2024)	China (10)	-0.789	0.0000	***
Fertilization	Grain vs protein	Zhang et al. (2017)	China (12)	-0.801	0.0000	***
Fertilization	Grain vs Fe	Kiran et. al.(2021)	Pakistan (12)	0.934	0.0000	***
Fertilization	Grain vs Fe	Zia et al. (2020)	Pakistan (48)	0.642	0.0000	***
Fertilization	Grain vs Fe	Reznick et al. (2021)	Brazil (12)	0.630	0.0018	**
Fertilization	Grain vs Fe	Ramzan et al. (2020)	Pakistan (14)	0.580	0.0038	**
Fertilization	Grain vs Fe	Hafeez et al.(2021)	Pakistan (16)	0.450	0.0416	*
Fertilization	Grain vs Fe	Anwar et al. 2021	Pakistan (20)	0.384	0.0629	ns
Fertilization	Grain vs Fe	Gao et al. (2012)	Canada (156)	0.367	0.0000	**
Fertilization	Grain vs Fe	Xia et al. (2020)	China (12)	0.275	0.3786	ns
Fertilization	Grain vs Fe	Montoya et. al. (2020)	Spain (15)	0.208	0.4603	ns
Fertilization	Grain vs Fe	Niyigaba et al. (2019)	China (38)	0.144	0.3912	ns
Fertilization	Grain vs Fe	Qamari et al. (2023)	Iran (42)	0.112	0.4884	ns
Fertilization	Grain vs Fe	El-Dahshouri et al. (2017)	Egypt (16)	-0.022	0.9422	ns
Fertilization	Grain vs Fe	Hao et al. (2021)	China (279)	-0.032	0.6069	ns
Fertilization	Grain vs Fe	Taskin and Gunes (2022)	Turkey (14)	-0.048	0.8825	ns
Fertilization	Grain vs Fe	Sher et al. (2020)	Pakistan (27)	-0.093	0.6588	ns
Fertilization	Grain vs Fe	Zou et al. (2019)	Global (108)	-0.134	0.1627	ns
Fertilization	Grain vs Fe	Souza et al. (2019)	Brazil (10)	-0.267	0.4556	ns
Fertilization	Grain vs Fe	Lu et al. (2024)	China (10)	-0.362	0.2737	ns
Fertilization	Grain vs Fe	Kandil et al. (2017)	Egypt (36)	-0.451	0.0012	**
Fertilization	Grain vs Fe	Milvojevic et al. (2018)	Serbia (30)	-0.465	0.0021	**
Fertilization	Grain vs Fe	Zhang et al. (2010)	China (15)	-0.567	0.0038	**
Fertilization	Grain vs Fe	Cakmak et.al.(2010)	Turkey (24)	-0.677	0.0000	***
Fertilization	Grain vs Zn	Singh & Sandhu (2021)	India (13)	0.973	0.0000	***
Fertilization	Grain vs Zn	Akca & Taban (2024)	Turkey (28)	0.959	0.0000	***
Fertilization	Grain vs Zn	Khalili et al. (2023)	Pakistan (12)	0.950	0.0000	***
Fertilization	Grain vs Zn	Hussan et al. (2021)	Pakistan (18)	0.868	0.0000	***
Fertilization	Grain vs Zn	Abdi et al. (2013)	Pakistan (20)	0.852	0.0000	***
Fertilization	Grain vs Zn	El-Dahshouri et al. (2017)	Egypt (16)	0.847	0.0000	***
Fertilization	Grain vs Zn	Chattha et. al. (2017)	Pakistan (30)	0.841	0.0000	***
Fertilization	Grain vs Zn	Kiran et. al.(2021)	Pakistan (12)	0.841	0.0000	***
Fertilization	Grain vs Zn	Akram et. al.(2017)	Pakistan	0.755	0.0000	***
Fertilization	Grain vs Zn	Ali et al. (2024)	Pakistan (23)	0.741	0.0000	***
Fertilization	Grain vs Zn	Mosavian et al. (2021)	Iran (36)	0.681	0.0000	***
Fertilization	Grain vs Zn	Ramzan et al. (2020)	Pakistan (14)	0.662	0.0001	**
Fertilization	Grain vs Zn	Khoshgoftarmanesh et al. (2005)	Iran (19)	0.628	0.0000	***
Fertilization	Grain vs Zn	Nadeem et. al. (2023)	Pakistan (16)	0.596	0.0009	***
Fertilization	Grain vs Zn	Vaziri et al. (2023)	Iran (36)	0.539	0.0000	***
Fertilization	Grain vs Zn	Liu et. al.(2017)	China (30)	0.530	0.0001	**
Fertilization	Grain vs Zn	Shah et al. (2023)	Pakistan (16)	0.495	0.0180	*
Fertilization	Grain vs Zn	Ivanovic et al. (2021)	Serbia (32)	0.460	0.0017	***
Fertilization	Grain vs Zn	Kandil et al. (2017)	Egypt (36)	0.460	0.0008	***
Fertilization	Grain vs Zn	Rashid et al. (2019)	Global (57)	0.454	0.0000	***
Fertilization	Grain vs Zn	Hafeez et al.(2021)	Pakistan (16)	0.449	0.0422	*
Fertilization	Grain vs Zn	Yilmaz et al. (1997)	Turkey (18)	0.441	0.0337	*
Fertilization	Grain vs Zn	Sher et al. (2020)	Pakistan (27)	0.390	0.0240	*
Fertilization	Grain vs Zn	Shariatipour et al. (2020)	Iran (48)	0.385	0.0025	**
Fertilization	Grain vs Zn	Souza et al. (2019)	Brazil (10)	0.374	0.2527	ns
Fertilization	Grain vs Zn	Xia et al. (2020)	China (12)	0.330	0.2698	ns
Fertilization	Grain vs Zn	Dhaliwal et. al.(2019)	India (15)	0.321	0.2169	***
Fertilization	Grain vs Zn	Sadhegi et al. (2021)	Iran (16)	0.306	0.2256	ns
Fertilization	Grain vs Zn	Biswas et. al.(2015)	India (20)	0.285	0.2024	ns
Fertilization	Grain vs Zn	Montoya et. al. (2020)	Spain (15)	0.281	0.2945	ns
Fertilization	Grain vs Zn	Qamari et al. (2023)	Iran (42)	0.141	0.3753	ns
Fertilization	Grain vs Zn	Ram et. al. (2013)	India (16)	0.089	0.7592	ns
Fertilization	Grain vs Zn	Zou et al. (2019)	Global (108)	0.037	0.7174	ns
Fertilization	Grain vs Zn	Zhang et al. (2010)	China (15)	0.001	0.9972	ns
Fertilization	Grain vs Zn	Barut et.al.(2017)	Turkey (72)	-0.017	0.8962	ns
Fertilization	Grain vs Zn	Anwar et al. 2021	Pakistan (20)	-0.051	0.8440	ns
Fertilization	Grain vs Zn	Dhaliwal et.al. (2023)	India (16)	-0.053	0.8584	ns
Fertilization	Grain vs Zn	Meena et. al.(2021b)	India (23)	-0.074	0.7513	ns
Fertilization	Grain vs Zn	Gomez-Coronado et. al.(2017)	Portugal (30)	-0.088	0.6579	ns
Fertilization	Grain vs Zn	Ram et. al. (2016)	Global (72)	-0.112	0.3518	ns
Fertilization	Grain vs Zn	Luis et al. (2021)	Portugal (36)	-0.121	0.4903	ns

Fertilization	Grain vs Zn	Hao et al. (2021)	China (279)	-0.176	0.0026	**
Fertilization	Grain vs Zn	Zou et al. (2012)	Global (91)	-0.223	0.0275	*
Fertilization	Grain vs Zn	Ma et al. (2018)	China (48)	-0.280	0.0412	*
Fertilization	Grain vs Zn	Ning et. al. (2019)	China (18)	-0.283	0.2358	ns
Fertilization	Grain vs Zn	Niyigaba et al. (2019)	China (38)	-0.295	0.0555	ns
Fertilization	Grain vs Zn	Peck et al. (2008)	Australia (21)	-0.353	0.0868	ns
Fertilization	Grain vs Zn	Cakmak et.al.(2010)	Turkey (24)	-0.357	0.0604	ns
Fertilization	Grain vs Zn	Xu et al. (2022)	China (24)	-0.402	0.0278	*
Fertilization	Grain vs Zn	Gomez-Coronado et. al.(2016)	Portugal (104)	-0.451	0.0000	***
Fertilization	Grain vs Zn	Zia et al. (2020)	Pakistan (48)	-0.580	0.0000	***
Fertilization	Grain vs Zn	Lu et al. (2024)	China (10)	-0.591	0.0162	*
Fertilization	Grain vs Zn	Porter & Paulsen (1983)	USA (90)	-0.623	0.0000	***
Fertilization	Grain vs Zn	Gao et al. (2012)	Canada (156)	-0.640	0.0000	***
Fertilization	Grain vs Zn	Milvojevic et al. (2018)	Serbia (30)	-0.640	0.0000	***
Fertilization	Grain vs Zn	Reznick et al. (2021)	Brazil (12)	-0.641	0.0011	**
Fertilization	Grain vs Zn	Hui et al. (2019)	China (15)	-0.783	0.0000	***
Fertilization	Grain vs Zn	Curtin et. al.(2008)	Ne Zealand (12)	-0.852	0.0000	***
Fertilization	Grain vs Zn	Zhang et al. (2017)	China (12)	-0.911	0.0000	***
Fertilization	Zn vs Fe	Kiran et. al.(2021)	Pakistan (12)	0.857	0.0000	***
Fertilization	Zn vs Fe	Ghasemi et al. (2013)	Iran (20)	0.786	0.0000	***
Fertilization	Zn vs Fe	Lu et al. (2024)	China (10)	0.778	0.0000	***
Fertilization	Zn vs Fe	Xia et al. (2020)	China (12)	0.676	0.0002	***
Fertilization	Zn vs Fe	Cakmak et.al.(2010)	Turkey (24)	0.675	0.0000	***
Fertilization	Zn vs Fe	Montoya et. al. (2020)	Spain (15)	0.654	0.0001	***
Fertilization	Zn vs Fe	Hafeez et al.(2021)	Pakistan (16)	0.566	0.0027	**
Fertilization	Zn vs Fe	Zou et al. (2019)	Global (108)	0.498	0.0000	***
Fertilization	Zn vs Fe	Qamari et al. (2023)	Iran (42)	0.482	0.0001	***
Fertilization	Zn vs Fe	Milvojevic et al. (2018)	Serbia (30)	0.469	0.0018	**
Fertilization	Zn vs Fe	Souza et al. (2019)	Brazil (10)	0.461	0.1214	ns
Fertilization	Zn vs Fe	Hao et al. (2021)	China (279)	0.435	0.0000	***
Fertilization	Zn vs Fe	El-Dahshouri et al. (2017)	Egypt (16)	0.329	0.1845	ns
Fertilization	Zn vs Fe	Kandil et al. (2017)	Egypt (36)	0.298	0.0599	ns
Fertilization	Zn vs Fe	Zhang et al. (2010)	China (15)	0.283	0.2904	ns
Fertilization	Zn vs Fe	Ramzan et al. (2020)	Pakistan (14)	0.240	0.4056	ns
Fertilization	Zn vs Fe	Sadhegi et al. (2021)	Iran (16)	0.225	0.3999	ns
Fertilization	Zn vs Fe	Sher et al. (2020)	Pakistan (27)	-0.055	0.7992	ns
Fertilization	Zn vs Fe	Niyigaba et al. (2019)	China (38)	-0.099	0.5657	ns
Fertilization	Zn vs Fe	Zia et al. (2020)	Pakistan (48)	-0.132	0.3738	ns
Fertilization	Zn vs Fe	Gao et al. (2012)	Canada (156)	-0.218	0.0047	ns
Fertilization	Zn vs Fe	Reznick et al. (2021)	Brazil (12)	-0.501	0.0444	*
Fertilization	Zn vs phytate	Ning et. al. (2019)	China (18)	0.400	0.0647	ns
Fertilization	Zn vs phytate	Lu et al. (2024)	China (10)	0.151	0.6958	ns
Fertilization	Zn vs phytate	Gomez-Coronado et. al.(2016)	Portugal (104)	0.138	0.1580	ns
Fertilization	Zn vs phytate	Ghasemi et al. (2013)	Iran (20)	0.096	0.7027	ns
Fertilization	Zn vs phytate	Akca & Taban (2024)	Turkey (28)	0.074	0.7230	ns
Fertilization	Zn vs phytate	Hao et al. (2021)	China (279)	0.044	0.4732	ns
Fertilization	Zn vs phytate	Xia et al. (2018)	China (36)	0.017	0.9287	ns
Fertilization	Zn vs phytate	Gomez-Coronado et. al.(2017)	Portugal (30)	-0.215	0.2440	ns
Fertilization	Zn vs phytate	Bharti et. al. (2013)	India (60)	-0.523	0.0000	***
Fertilization	Zn vs phytate	Khalili et al. (2023)	Pakistan (12)	-0.718	0.0000	**
Fertilization	Zn vs phytate	Zhang et al. (2017)	China (12)	-0.853	0.0000	***
Fertilization	Zn vs phytate	Chattha et. al. (2017)	Pakistan (30)	-0.945	0.0000	***
Fertilization	Zn vs protein	Singh & Sandhu (2021)	India (13)	0.992	0.0000	***
Fertilization	Zn vs protein	Xia et al. (2020)	China (12)	0.954	0.0000	***
Fertilization	Zn vs protein	Hussan et al. (2021)	Pakistan (18)	0.927	0.0000	***
Fertilization	Zn vs protein	Akram et. al.(2017)	Pakistan (27)	0.904	0.0000	***
Fertilization	Zn vs protein	Reznick et al. (2021)	Brazil (12)	0.895	0.0000	***
Fertilization	Zn vs protein	Zhang et al. (2017)	China (12)	0.892	0.0000	***
Fertilization	Zn vs protein	El-Dahshouri et al. (2017)	Egypt (16)	0.797	0.0000	***
Fertilization	Zn vs protein	Ning et. al. (2019)	China (18)	0.758	0.0000	***
Fertilization	Zn vs protein	Ghasemi et al. (2013)	Iran (20)	0.754	0.0000	***
Fertilization	Zn vs protein	Montoya et. al. (2020)	Spain (15)	0.615	0.0006	***
Fertilization	Zn vs protein	Mosavian et al. (2021)	Iran (36)	0.612	0.0000	***
Fertilization	Zn vs protein	Gao et al. (2012)	Canada (156)	0.587	0.0000	***
Fertilization	Zn vs protein	Biswas et. al.(2015)	India (20)	0.488	0.0083	**
Fertilization	Zn vs protein	Kandil et al. (2017)	Egypt (36)	0.453	0.0011	**
Fertilization	Zn vs protein	Cakmak et.al.(2010)	Turkey (24)	0.441	0.0121	*
Fertilization	Zn vs protein	Nadeem et. al. (2023)	Pakistan (16)	0.417	0.0684	ns
Fertilization	Zn vs protein	Niyigaba et al. (2019)	China (38)	0.402	0.0046	*
Fertilization	Zn vs protein	Gomez-Coronado et. al.(2017)	Portugal (30)	0.378	0.0218	*

Fertilization	Zn vs protein	Kiran et. al.(2021)	Pakistan (12)	0.342	0.2479	ns
Fertilization	Zn vs protein	Lu et al. (2024)	China (10)	0.279	0.4315	ns
Fertilization	Zn vs protein	Ma et al. (2018)	China (48)	0.248	0.0759	ns
Fertilization	Zn vs protein	Souza et al. (2019)	Brazil (10)	0.198	0.5977	ns
Fertilization	Zn vs protein	Ramzan et al. (2020)	Pakistan (14)	0.091	0.7736	ns
Fertilization	Zn vs protein	Qamari et al. (2023)	Iran (42)	0.089	0.5873	ns
Fertilization	Zn vs protein	Barut et.al.(2017)	Turkey (72)	-0.098	0.4188	ns
Fertilization	Zn vs protein	Hafeez et al.(2021)	Pakistan (16)	-0.780	0.0000	***

References

Bogard M, Allard V, Brancourt-Hulmel M, Heumez E, Machet J-M, Jeuffroy MH, Gate P, Martre P, Le Gouis J (2010) Deviation from the grain protein concentration–grain yield negative relationship is highly correlated to post-anthesis N uptake in winter wheat. Journal of Experimental Botany 61: 4303–4312. doi:10.1093/jxb/erq238.

Covarrubias-Pazaran GE (2019) Heritability: meaning and computation. A manual, Excellence in Breeding. https://excellenceinbreeding.org/sites/default/files/manual/EiB-M2_Heritability_18-02-20.pdf. Accessed: 23/02/2025

Evans LM, Tahmasbi R, Jones M, Vrieze SI, Abecasis GR, Das S, Bjelland DW, de Candia TR, Yang J, Goddard ME, Visscher PM, Keller MC; Haplotype Reference Consortium (2018). Narrow-sense heritability estimation of complex traits using identity-by-descent information. Heredity 121(6): 616-630. doi: 10.1038/s41437-018-0067-0.

Zheng, J.; Zhang, S. (2025) Assessing the impact of climate change on winter wheat production in the North China Plain from 1980 to 2020. Agriculture 15, 449. https://doi.org/10.3390/agriculture15050449

Supplementary_Information_Code_used

SAS code for running linear mixed effects models

The following codes were used for the various analyses but models were adjusted based on the goodness of fit statistics, i.e., AIC, BIC generated for any given combination of explanatory variables Xi. The final model was the one that minimized the AIC and BIC.

Proc sort; By variable; */*requests sorting the analysis by the response variables, i.e., lnRR of grain yield, Zn, Fe, protein, phytate*/*
proc mixed data= Wheat method = REML covtest; */*requests a linear mixed effects procedure for the dataset named "Wheat" using a restricted maximum likelihood and display the covariance test results*/*
Class X1 X2 X3 Xn Study; */Specifies X1, X2, X3, X4 and Study as the classification variables*/*
model lnRR = X1, X2, X3, X4/solution ddfm = kr; */*specifies independent variables X1 to X4 as the fixed effects and computation of the denominator degrees of freedom using the Kenward Roger method*/*
Random Study; */*specifies study as the random effect*/*
LSmeans X1/diff adjust = Tukey; */*requests marginal means for lnRR and 95% CI for the different classes of the independent variable X1, for example soil pH and Tukey-Kramer adjusted P value*/*
By variable; */*requests display of analysis for each*

R codes for Random Forest model

```
rm(list=ls(all=TRUE))
setwd("C:\\........\\Data set")
Data <- read.csv("Data.csv", header=T)
Data <- Data[, c(x,x1,x2,……..)]
library(randomForest)
set.seed(1234567)
rownames(Data)<-1:nrow(Data)
rows <- sample(x=1:nrow(Data),size=0.8 *  nrow(Data))
train <- sample(nrow(Data), 0.8*nrow(Data), replace = FALSE)
TrainSet <- Data [train,]
ValidSet <- Data [-train,]
library(randomForest)
set.seed(1234567)
selected<-c(as.character(imp[1:25, 1]),'variable')
model1<-randomForest(variable ~.,data=TrainSet[,,],replace=T,ntree=1000, type=regression,
na.action=na.omit)
model1
par(mfrow=c(1,2))
varImpPlot(model1,main='Variable Importance Plot: Final Model',pch=16,col='blue')
plot(model0, main='Error vs No. of trees plot: Final Model',col='blue')
pred<-predict(object=model1,newdata=ValidSet)
actual<-ValidSet$variable
result<-data.frame(actual=actual,predicted=pred)
paste('Mean Squared error: ',mean(model1$mse))
paste('Root Mean Squared error: ',mean(sqrt(model1$mse)))
write.csv(result, "rf.csv")
mf <- read.csv("rf.csv", header=T)
ls(mf)
RF.rmse<-round(sqrt(mean( (mf$actual-mf$predicted)^2 , na.rm = TRUE )),0)
print(RF.rmse)
RF.r2<-round(summary(lm(actual~predicted, mf))$r.squared,3)
print(RF.r2)
#plotting predicted Vs observed
library(ggplot2)
```

```
plot1<-ggplot(result)+
  geom_point(aes(x=actual,y=predicted),alpha=0.6)+
  ggtitle('Grain yield') +
  scale_x_continuous("Observed grain yield (kg ha⁻¹)",
                    limits=c(500,8000),
                    breaks=seq(x, x1, x2))+
  scale_y_continuous("Predicted grain yield (kg ha⁻¹)",
                    limits=c(500,8000),
                    breaks=seq(500, 8000, 1000)) +
  theme(axis.line = element_line(colour = "black"),
        axis.text.y=element_text(size=14,angle = 90, hjust = 0.5, vjust=1),
        axis.text.x = element_text(size=14)) +
  geom_abline(intercept=0,size = 0.5)+
  geom_smooth(aes(x=actual,y=predicted), method = "lm", se = FALSE, colour="red",linetype = 2,size =
0.75)+
  annotate("text", x=.., y=…, label= paste("RMSE:",RF.rmse), frontface= "italic", colour="black", size=6)+
  annotate("text", x=…, y=…, label= paste("R^2: " ,RF.r2), frontface= "italic", colour="black", size=6, parse=T)
plot1
plot2<-plot1+
  theme(
    axis.title.x = element_text(colour="black", size=..),
    axis.text.x = element_text(colour="black", size=..),
    axis.title.y = element_text(colour="black", size=20, angle = 90),
    axis.text.y = element_text(colour="black", size=20),
    plot.title = element_text(colour="black", size=18, face="bold"))
plot1+theme(
  legend.background = element_rect(fill="grey85", colour="red", size=1),
  legend.title = element_text(colour="blue", face="bold", size=18),
  legend.text = element_text(colour="red", size=…),
  legend.key = element_rect(colour="blue", size=…))
plot2
paste('Function Call: ', model1$call)
```
